# Spin state switching in iron coordination compounds

**DOI:** 10.3762/bjoc.9.39

**Published:** 2013-02-15

**Authors:** Philipp Gütlich, Ana B Gaspar, Yann Garcia

**Affiliations:** 1 Institut für Anorganische Chemie und Analytische Chemie, Johannes Gutenberg-Universität, Staudingerweg 9, 55099 Mainz, Germany; 2 Institut de Ciència Molecular (ICMOL)/Departament de Química Inorgànica, Universitat de València, Edifici de Instituts de Paterna, Apartat de Correus 22085, 46071 València, Spain; 3 Institute of Condensed Matter and Nanosciences, MOST – Inorganic Chemistry, Université Catholique de Louvain, Place L. Pasteur 1, 1348 Louvain la Neuve, Belgium

**Keywords:** cages, iron(II) coordination compounds, physical techniques, polyfunctional materials, spin crossover

## Abstract

The article deals with coordination compounds of iron(II) that may exhibit thermally induced spin transition, known as spin crossover, depending on the nature of the coordinating ligand sphere. Spin transition in such compounds also occurs under pressure and irradiation with light. The spin states involved have different magnetic and optical properties suitable for their detection and characterization. Spin crossover compounds, though known for more than eight decades, have become most attractive in recent years and are extensively studied by chemists and physicists. The switching properties make such materials potential candidates for practical applications in thermal and pressure sensors as well as optical devices.

The article begins with a brief description of the principle of molecular spin state switching using simple concepts of ligand field theory. Conditions to be fulfilled in order to observe spin crossover will be explained and general remarks regarding the chemical nature that is important for the occurrence of spin crossover will be made. A subsequent section describes the molecular consequences of spin crossover and the variety of physical techniques usually applied for their characterization. The effects of light irradiation (LIESST) and application of pressure are subjects of two separate sections. The major part of this account concentrates on selected spin crossover compounds of iron(II), with particular emphasis on the chemical and physical influences on the spin crossover behavior. The vast variety of compounds exhibiting this fascinating switching phenomenon encompasses mono-, oligo- and polynuclear iron(II) complexes and cages, polymeric 1D, 2D and 3D systems, nanomaterials, and polyfunctional materials that combine spin crossover with another physical or chemical property.

## Introduction

Coordination compounds of transition-metal ions may, under certain conditions, exhibit a switching phenomenon, whereby the central metal ion changes the spin state upon a change of temperature, application of pressure, irradiation with light, or in a magnetic field. This phenomenon is known as *spin transition* (ST) or *spin crossover* (SCO). The change of spin state is accompanied by a change of electronic structure of the central ion and the complex molecule on the whole, which changes markedly the physical and chemical properties of the substance. Most spectacular is the change of magnetic behavior and color, which has made such SCO substances very attractive because of their potential for practical applications, e.g., as switching devices and sensors.

A thermally induced change of spin state (spin transition, spin crossover) was first reported some eighty years ago by Cambi and co-workers. They prepared a great variety of dithiocarbamato complexes of iron(III), by varying the substituents at the dithiocarbamate ligands, and investigated their magnetic properties. From magnetic susceptibility measurements at room temperature they found that some of the samples showed magnetic moments corresponding to five unpaired electrons (later on denoted as *high-spin state,* HS) and others with different substituents showed magnetic moments corresponding to only one unpaired electron (*low-spin state,* LS). A third class of such complexes exhibited very unusual magnetic properties: they showed HS behavior at room temperature, but changed more or less gradually to LS behavior on cooling [1–6]. Many more SCO complex compounds of iron(III) have been synthesized thereafter and extensively investigated [7–13].

More than thirty years after the discovery of thermal ST by Cambi et al. the first iron(II) coordination compound, viz. [Fe(phen)_2_(NCS)_2_] (phen = 1,10-phenanthroline), was observed to show also thermally induced ST between HS and LS states: the spin transition takes place very abruptly near to 175 K [14–15]. Since then, many further examples of iron(II) SCO compounds have been published [7–13,16–28], and other coordination compounds of 3d transition elements such as cobalt(II) [29–30], and to a much lesser extent cobalt(III), chromium(II), manganese(II), manganese(III), and nickel were found to exhibit thermal ST phenomena [31–33]. But practically no example of thermal ST with coordination compounds of the 4d and 5d transition metal series has been reported up to now, which is well understood on the basis of ligand field theory [34–35].

The purpose of this article is to introduce researchers, mainly from the vast field of organic chemistry, to the fascinating SCO switching phenomenon occurring in inorganic coordination compounds of transition-metal ions. In the first part we shall describe the principle of thermal spin crossover and methods of physical characterization, and demonstrate with a selection of typical examples, mainly SCO compounds of iron(II), the variety of chemical and physical influences on the spin transition behavior. The second part will be devoted to a brief overview of selected SCO compounds of iron(II) including mono-, di-, oligonuclear and higher nuclearity complexes, polymeric 1D, 2D and 3D systems, 1D chain compounds and 2D and 3D networks, SCO in nanomaterials, and soft matter, such as metallomesogens, as examples for the main current objectives in SCO research, viz. synthesizing so-called multifunctional materials, which combine the SCO switching phenomenon with other functionalities. The limited scope of the present account has not enabled us to present a rigorous quantitative review aimed at researchers actively working in this field. We extend our sincere apologies to all those who have contributed and published excellent SCO work, which, because of limited space, we were not able to include here.

The article concludes with an outlook, emphasizing on the one hand the current activities in SCO research towards arriving at a better understanding of the molecular processes and cooperative interactions during the spin transition, which is of utmost importance for eventual practical applications of SCO materials; and on the other hand, the necessity and usefulness of close cooperation between organic and inorganic chemists will be pointed out in view of the nature and rich variety of SCO compounds, where (often sophisticated) organic ligand molecules are coordinated to transition-metal ions stimulating and controlling the electronic switching phenomenon.

## Review

### Occurrence of spin transition

**Figure 1:**
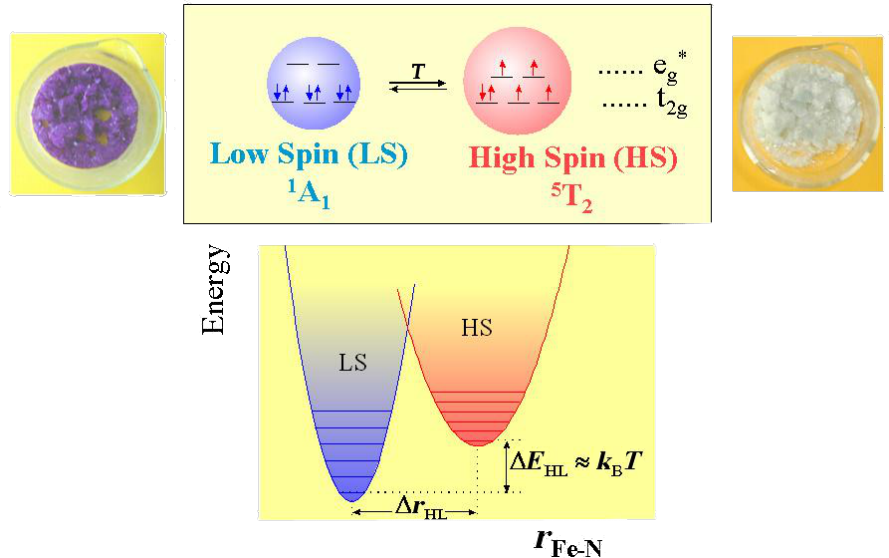
Change of electron distribution between HS and LS states of an octahedral iron(II) coordination compound exhibiting thermal spin crossover. The orbitals e_g_^*^ and t_2g_ arise from splitting of the 3d orbitals in an octahedral ligand field. Depletion of charge in the antibonding e_g_^*^ orbitals during HS to LS transition shortens the metal-to-ligand bond distances and reduces the molecular volume. SCO is mostly accompanied by a color change, e.g., in the present case, from white in the HS state to violet in the LS state. The condition to fulfil in order to observe thermal spin crossover is Δ*E*_HL_* ≈ k*_B_*T.* Increasing the temperature favors the HS state, decreasing it favors the LS state [23]. (Reproduced with permission from [36]. Copyright 2012 Wiley-VCH Verlag GmbH & Co. KGaA).

The occurrence of ST in coordination compounds of transition-metal ions is governed by the relationship between the strength of the ligand field (the electrostatic field acting at the central metal ion) and the mean spin-pairing energy [34–35]. Octahedral complexes of d^4–7^ ions may be either HS or LS, depending on whether the ligand field strength is weaker or stronger, respectively, than the spin pairing energy. In order for thermally induced ST to occur the difference in the Gibb’s free energies for the two spin states involved must be on the order of thermal energy, *k*_B_*T* [23]. An increase in temperature favors the HS state, while lowering the temperature favors the LS state. The condition for thermal ST to occur and the consequences of ST are depicted in Figure 1 [23].

Thermal spin transition occurs nearly exclusively with coordination complexes of 3d metal ions. This is not expected for 4d and 5d transition element compounds on the basis of ligand field theory, because the strength of the ligand field increases notably (by ca. 50% from 3d to 4d and also from 4d to 5d) relative to analogous 3d compounds and is generally much greater than the spin pairing energy; hence virtually all 4d and 5d transition metal complexes show LS behavior.

Spin transition seems to occur predominantly for six-coordinate iron(II) complexes with the following change of electron configurations and ligand field states:

[1]
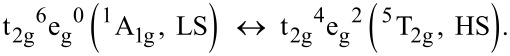


SCO complexes of iron(II) have been most extensively characterized and explored; the field has been reviewed many times [7–13,16–28]. Most of the iron(II) SCO compounds known possess an [N_6_] donor atom set, i.e., an [FeN_6_] chromophor with N-coordinated ligands of variable denticity. A few examples with other donor-atom sets, e.g., N_4_O_2_ [37–40], P_4_Cl_2_, have also been reported [41]. [FeN_6_] systems of iron(II) involve, for instance, [Fe(diimine)_2_(NCS)_2_] complexes, among them the *classical* complexes with 1,10-phenanthroline (phen) and 2,2'-bipyridine (bipy) as bisdentate diimine ligands, which were among the first SCO compounds of iron(II) reported in the literature [14–15]. An example of [FeN_6_] complexes with trisdentate N-donor ligands is bis[hydro-tris(pyrazolyl-borato)]iron(II) [42–43] and related systems.

Thermal SCO has been observed with [Fe(diimine)_2_(NCS)_2_] complexes employing a large variety of diimine ligands and also with iron(II) complexes containing derivatives of unidentate pyridine [44], bridging diimine and bis(unidentate) ligand components. The anionic groups X in [Fe(diimine)_2_X_2_] complexes, where X is directly coordinated to the central metal ion, can be varied too, and SCO has been reported for [Fe(diimine)_2_X_2_] systems with X^–^ = NCSe^–^ [14–15], [NCBH_3_]^–^ [45], TCNQ^–^ [46], [N(CN)_2_]^–^ [47], and 2X^–^ = [WS_4_]^2−^ [48].

Many [FeN_6_]^2+^ systems are based on the cationic LS [Fe(2,2”-bipyridine)_3_]^2+^ or [Fe(2,2":2',6'-terpyridine)_2_]^2+^ ions, where the critical field strength to reach the crossover condition is fulfilled by introducing sterically hindering groups adjacent to the donor atoms or by replacing the pyridine rings by five-membered heterocycles [25–26]. Examples are the SCO systems tris(6-methyl-2,2'-bipyridine)iron(II) and bis(2,4-bis(pyridin-2-yl)thiazole)iron(II) ions [49]. The [FeN_6_] system can also exhibit SCO behavior when six monodentate N-donating ligand molecules are involved. The best known examples are the [Fe(N-alkyl-tetrazole)_6_]X_2_ complexes, which are nearly regular octahedral [50]. Another possibility to fulfill the condition for thermal spin crossover to occur is the coordination of a hexadentate system, such as the mixed aliphatic/heterocyclic tetrakis(2-pyridylmethyl)ethylenediamine) [51] or the completely saturated, encapsulating hexadentate donor system as described by Martin et al. [52].

**Figure 2:**
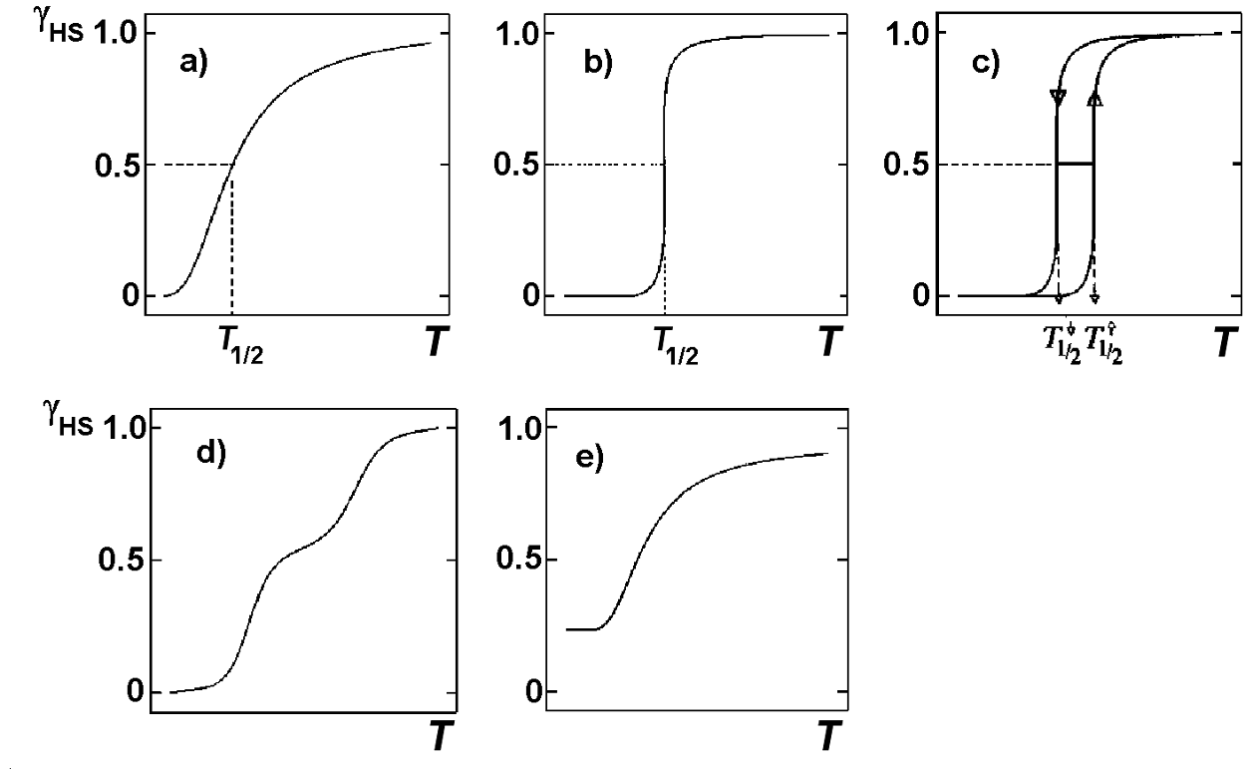
Types of spin transition curves in terms of the molar fraction of HS molecules, *γ*_HS_(*T*), as a function of temperature [23]. (Reproduced with permission from [27]. Copyright 2003 Wiley-VCH Verlag GmbH & Co. KGaA).

Through extensive synthetic work one has learned that *cooperative interactions* between the spin-state-changing complex molecules are of utmost importance for the ST behavior in solid SCO compounds. There are essentially three synthetic strategies that have been developed to create and strengthen cooperativity, i.e., (i) incorporation of a hydrogen-bonded network; (ii) incorporation of π-stacking moieties; and (iii) coordination of bridging ligands. The thermal ST behavior is commonly expressed in terms of the molar fraction of HS molecules, *γ*_HS_(*T*), as a function of temperature denoted as the ST function. Spin transitions have been categorized into the types depicted in Figure 2, i.e., gradual (a), abrupt (b), with hysteresis (c), step-wise (d) and incomplete (e). Gradual spin transitions (type a) appear in solution, where practically no cooperative interactions exist and the ST curve follows a simple Boltzmann distribution over all energy levels involved. In solid material, however, the electronic and structural changes accompanying the ST propagate throughout the solid through short and long range interactions and influence markedly the ST function *γ*_HS_(*T*).

### Thermal spin transition in iron(II) compounds: Consequences and physical characterization

LS↔HS conversions are accompanied by profound changes in all properties that depend on the distribution of the 3d valence electrons, predominantly optical, vibrational, magnetic and structural properties. These molecular changes enable one to detect the occurrence of thermal SCO and monitor the temperature dependence of a ST, as sketched in Figure 2, employing various physical methods, primarily magnetic susceptibility measurements, optical and vibrational spectroscopy, Mössbauer spectroscopy, crystal-structure determination and heat-capacity measurements [23,27–28]. In the following we shall briefly discuss the main molecular changes that accompany LS↔HS conversions in iron(II) compounds and their detection with suitable physical methods. As will be shown in many examples, Mössbauer spectroscopy has proven to be a particularly elegant microscopic tool for the characterization of iron-containing SCO systems. It has been invaluable in detailed studies.

#### Magnetic susceptibility measurements

From the very beginning of experimental SCO research, measuring the magnetic susceptibility of a sample as a function of temperature, *χ*(*T*), and, if required, deriving the magnetic moment thereof has always been the main characterization method. The different number of unpaired electrons in the HS and LS states, e.g., in the case of iron(II) with four unpaired electrons in the strongly paramagnetic HS state and no unpaired electrons in the diamagnetic LS state, is readily signalized by a drastic change in the magnetic susceptibility. *χ*(*T*) can then be calculated by using Equation 2:

[2]
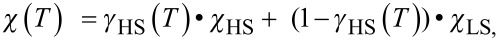


where *γ*_HS_ is the molar fraction of HS molecules, and *χ*_HS_ and *χ*_LS_ refer to the magnetic susceptibilities of the sample in the pure HS and LS states, which can be measured at sufficiently high and low temperatures, respectively, in the case of a complete spin state transition. The ST curve *γ*_HS_(*T*) can be readily obtained with these quantities. One can also derive the effective magnetic moment *μ_eff_* = 2.83 √*χT* [34,53–54] and plot it as a function of temperature. However, because of certain complications, e.g., orbital contribution and zero-field splitting effects, it has become common practice to study the ST behavior by plotting the *γ*_HS_(*T*) function rather than the magnetic moment *μ_eff_*(*T*).

Techniques for magnetochemical studies of solid material (Faraday balance, Foner type magnetometer, SQUID and AC devices) as well as solutions (Evans’ NMR method) are described in [55–56].

#### Optical spectroscopy

**Figure 3:**
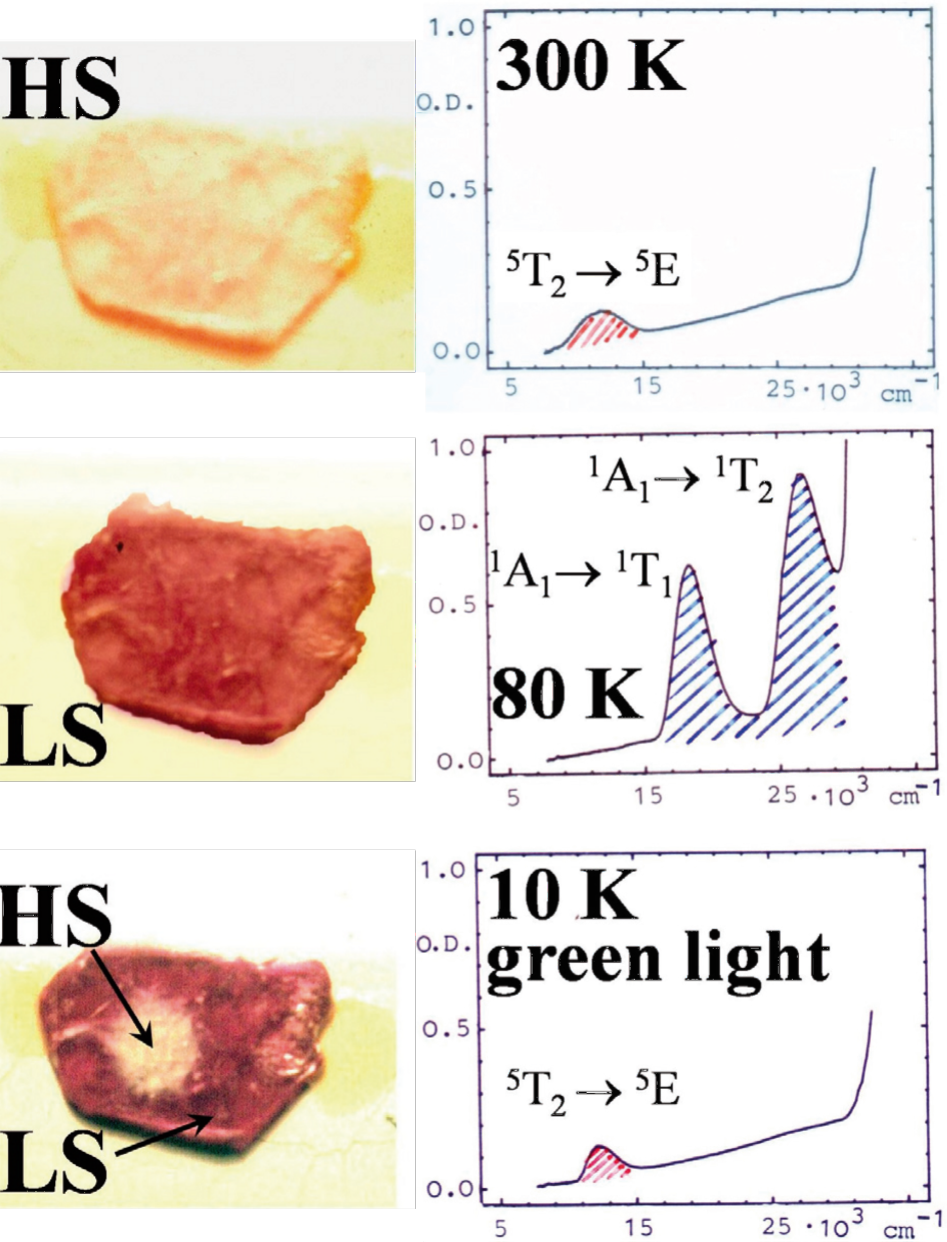
Single crystal UV–vis spectra of the spin crossover compound [Fe(ptz)_6_](BF_4_)_2_ (ptz = 1-propyltetrazole). At 300 K, the crystal is white and in the HS state. The spectrum shows a weak ^5^T_2_→^5^E band at ca. 12000 cm^−1^. At ca. 135 K, a spin transition takes place to the deeply colored LS state. At 80 K the crystal is red–purple, and the spectrum shows two spin-allowed d–d bands, ^1^A_1_→^1^T_1_ and ^1^A_1_→^1^T_2_ above 20000 cm^−1^. The LS state can be switched back to the long-lived metastable HS state by irradiation with green light. The optical spectrum recorded at 10 K shows the typical HS band again. This observation, known as the LIESST effect, is described in more detail below. (Reproduced with permission from [36]. Copyright 2012 Wiley-VCH Verlag GmbH & Co. KGaA).

Thermochromism is a typical feature that goes along with thermal ST in nearly all SCO compounds. The color change can be easily monitored by temperature-dependent optical spectroscopy in the UV–vis region. The electric dipole (E1) ligand field transitions (d–d transitions) involved are parity-forbidden but spin-allowed and can give rise to rather intense coloration, particularly in the LS state. For example, SCO complexes of iron(II) with tetrazole and triazole ligands are generally weakly colored or nearly white in the HS state but purple in the LS state. If such ligand field transitions are well resolved and not hidden by more intense parity- and spin-allowed charge-transfer bands, the optical spectrum recorded in the UV–visible region distinguishes well the two spin states involved and can therefore be employed to follow the ST phenomenon qualitatively and quantitatively. From the temperature-dependent area fractions of the absorption bands one can construct the ST curve *γ*_HS_(*T*). An example is displayed in Figure 3, where optical spectra (UV–vis) of a single crystal of the SCO compound [Fe(ptz)_6_](BF_4_)_2_ (ptz = 1-propyltetrazole), recorded at 300 K and 80 K, are shown [57–60].

#### Vibrational spectroscopy

As discussed above and shown in Figure 1 for the case of SCO in iron(II) compounds, thermal spin state conversion from HS to LS states is connected with depletion of charge in the antibonding e_g_ orbitals and simultaneous increase of charge in the lower lying slightly bonding t_2g_ orbitals on lowering of the temperature. This results in strengthening of the metal–donor-atom bonds and can be observed in the vibrational spectrum recorded in the far-infrared (FIR) region between ~250 and ~500 cm^−1^, where the central metal–donor-atom stretching frequencies of transition metal compounds generally appear [61–63]. Detailed and often tedious band assignment is not necessary. With temperature-dependent FIR or Raman spectroscopy, one can readily recognize the vibrational bands characteristic of the HS and the LS species as those with decreasing and increasing intensity, respectively, on cooling of the sample [64–70]. Although not often practiced, one can also derive the spin state conversion curve *γ*_HS_(*T*) (Figure 2) by plotting the normalized area fractions of characteristic HS or LS bands as a function of temperature.

A new method of studying vibrational properties has been developed based on *nuclear resonance scattering (NRS) with synchrotron radiation,* also denoted as *Mössbauer spectroscopy in the time domain* in comparison to classical *Mössbauer spectroscopy in the energy domain* [71–73]. NRS experiments are carried out in two ways: nuclear inelastic scattering (NIS) and nuclear forward scattering (NFS) of synchrotron radiation. The NIS technique has proven to be an excellent tool to identify typical HS and LS vibrational bands. One recognizes them as bands with temperature intensity arising in the near neighborhood of Mössbauer-active nuclides (e.g., ^57^Fe). They give information on the local densities of HS and LS vibrational states [74–76].

Spin state conversion can also affect certain ligand vibrations and can therefore be used to trace the spin state conversion curve *γ_HS_*(*T*). Examples are the N-coordinated ligands NCS^−^ and NCSe^−^, for instance in the “classical” systems [Fe(phen)_2_(NCS)_2_] and [Fe(phen)_2_(NCSe)_2_] [61–63]. The C–N stretching bands of NCS^−^ and NCSe^−^ are seen as a strong doublet near 2060–2070 cm^−1^ in the HS state. Upon cooling of [Fe(phen)_2_(NCS)_2_] to below the transition temperature (175 K), this doublet decreases in intensity, favoring the appearance of a new doublet at 2100–2110 cm^−1^, which obviously stems from the LS state [61–63]. One has also found that certain vibrational modes of lattice constituents, such as non-coordinating anions or solvent molecules, interconnecting the spin state changing metal complexes via hydrogen bonds, van der Waals or other interactions, can “feel” spin state changes at the metal centers and can therefore be made use of to study ST phenomena. As an example, one has found for the SCO compound [Fe(ptz)_6_](BF_4_)_2_ that certain B–F vibrations of the tetrafluoroborato anion show temperature-dependent intensity in agreement with results from magnetic measurements [59].

#### Mössbauer spectroscopy

**Figure 4:**
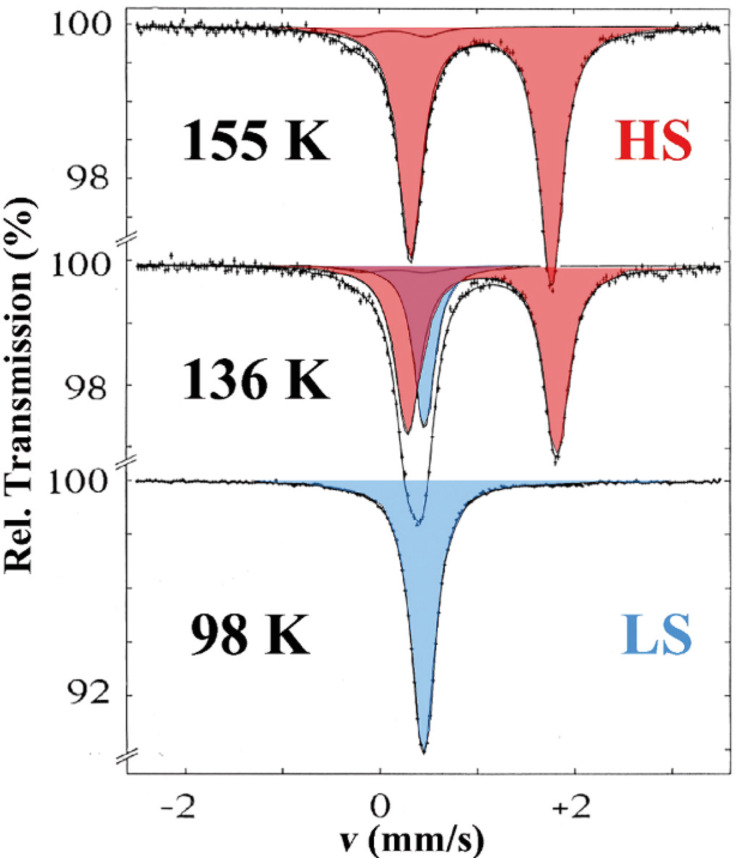
Thermal spin crossover in [Fe(ptz)_6_](BF_4_)_2_ (ptz = 1-propyltetrazole) recorded at three different temperatures. The spin transition temperature *T*_1/2_ is ca. 135 K. The ^57^Fe Mössbauer spectra demonstrate the transition between the HS phase (quadrupole doublet shown in red) and the LS phase (singlet shown in blue) at the spin transition temperature *T*_1/2_ of ca. 135 K [59] (Reproduced with permission from [36]. Copyright 2012 Wiley-VCH Verlag GmbH & Co. KGaA).

**Figure 5:**
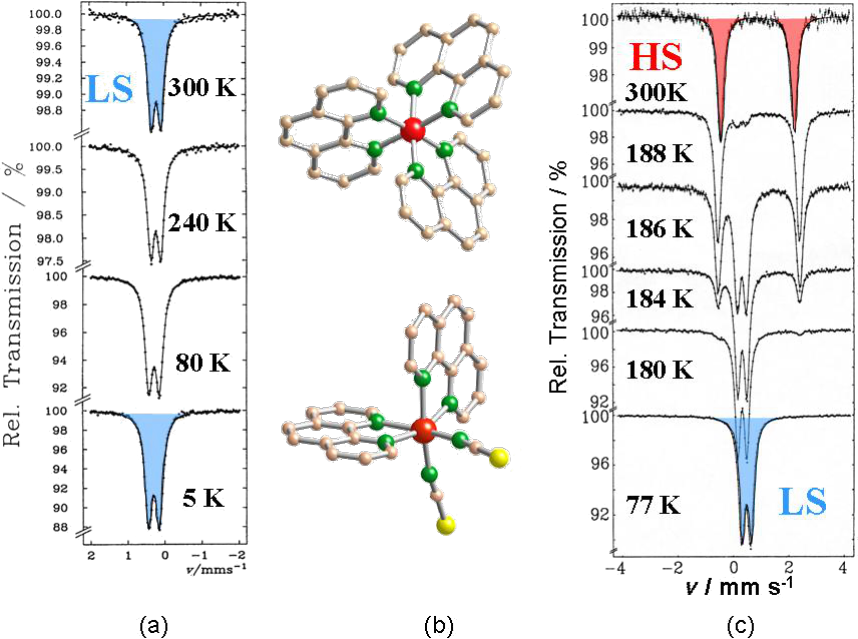
(a) Mössbauer spectra of the LS compound [Fe(phen)_3_]X_2_ recorded over the temperature range 300–5 K. (b) Perspective crystal structures of [Fe(phen)_3_]^2+^ (top) and [Fe(phen)_2_(NCS)_2_] (bottom). (c) Temperature-dependent Mössbauer spectra of [Fe(phen)_2_(NCS)_2_] indicating the thermally induced HS↔LS spin transition around 175 K [79] (Reproduced with permission from [36]. Copyright 2012 Wiley-VCH Verlag GmbH & Co. KGaA).

^57^Fe Mössbauer spectroscopy [36,77–81] has proved to be a most powerful tool in probing the oxidation and spin states of iron in coordination compounds. Two of the most important parameters derived from a Mössbauer spectrum, i.e., the *isomer shift* δ and the *quadrupole splitting* Δ*E_Q_*, vary significantly between iron(II)-HS and iron(II)-LS. The area fractions of the resonance signals are proportional to the concentrations of the coexisting spin states, and in most cases the ST curve *γ*_HS_(*T*) can be obtained by plotting the area fraction of one of the spin states, usually the HS state, as a function of temperature. Examples are displayed in Figure 4 and Figure 5. ^57^Fe Mössbauer spectra of the SCO compound [Fe(ptz)_6_](BF_4_)_2_ recorded at three different temperatures are displayed in Figure 4. The quadrupole doublet shown in red is characteristic of the HS state, being the only one present in the region above the critical ST temperature *T*_1/2_ at ca. 135 K; *T*_1/2_ is the temperature where both spin states HS and LS are present at 50% each. Below *T*_1/2_, the compound is in the LS state, and the resonance signal (blue) has changed dramatically. Details about the different isomer shift values and the origin and size of the quadrupole splitting of the two spin states of iron(II) are explained elsewhere [36,77–81].

Another instructive example is the study of the classical iron(II) SCO compound [Fe(phen)_2_(NCS)_2_] with ^57^Fe Mössbauer spectroscopy, which was first reported by Dézsi et al. [82]. This compound is derived from [Fe(phen)_3_]X_2_, which displays LS behavior over the entire temperature range from 300 to 5 K as confirmed by temperature-dependent Mössbauer spectroscopy (Figure 5a).

On replacement of one phen ligand by two weaker isothiocyanato anions the strength of the average ligand field acting at the central iron atom is weakened such that the condition for thermal SCO to occur is now fulfilled. This is corroborated by the temperature-dependent Mössbauer spectra presented in Figure 5c. The quadrupole doublet (marked in red) observed at room temperature, shows a large splitting of ca. 3 mm s^−1^, which is typical for HS Fe(II) compounds [79,82]. Upon cooling, a very abrupt spin state conversion (within a few Kelvin) to the LS state takes place near the spin transition temperature of ca. 175 K, signalized by the disappearance of the HS doublet in favor of a new doublet (blue) with considerably smaller quadrupole splitting energy of ca. 0.5 mm s^−1^, which is typical for LS Fe(II) compounds. The very sharp ST agrees well with data from magnetic susceptibility measurements [83].

It is worth noting that for iron(II) SCO compounds the HS↔LS fluctuation rate is on the order of ≤10^7^ s^−1^. This estimate is derived from the time window of ^57^Fe Mössbauer spectroscopy, which is determined by the mean life time *τ_N_* of ca. 140 ns of the first excited nuclear state of ^57^Fe. Since the subspectra arising from HS and LS states appear as sharp signals, their life times must be longer than *τ_N_*, and thus, the spin state fluctuation rate is 1/*τ_N_* ≤ 10^7^ s^−1^.

#### Calorimetry

Studies of SCO compounds often require a knowledge of thermodynamic quanitities such as the changes of enthalpy Δ*H* = *H*_HS_ − *H*_LS_ and entropy Δ*S* = S_HS_ − S_LS_ because of their valuable information regarding the ST properties, in particular cooperative interactions and the order of the ST transition. Enthalpy changes Δ*H* = *H*_HS_ − *H*_LS_ are typically 10 to 20 kJ mol^−1^, and entropy changes Δ*S* = *S*_HS_ − *S*_LS_ are on the order of 50 to 80 J mol^−1^ K^−1^ [22]. The thermally induced ST is an entropy-driven process because the degree of freedom is much higher in the HS state than in the LS state, which is schematically shown in Figure 1 by the narrower distances between the vibrational levels in the HS state as compared to the LS state. Approximately 25% of the total entropy gain on going from LS to HS is of magnetic origin, viz., it arises from the change in spin multiplicity, Δ*S*_mag_ = *R*[ln(2*S* + 1)_HS_ − ln(2*S* + 1)_LS_], ≈ 13 cal mol^−1^ K^−1^, and the major part originates from changes in the intramolecular, and to a much lesser extent, intermolecular vibrations [84–85].

The first precise heat capacity measurements on a SCO compound were performed by Sorai and Seki on the classical iron(II) SCO compounds [Fe(phen)_2_(NCX)_2_] with X = S, Se [84–85]. Other SCO compounds of Fe(II) [86–87], Fe(III) [88] and Mn(III) [89–90] have been studied down to liquid-helium temperature. Such measurements are very tedious. For qualitative information on enthalpy change Δ*H*, entropy change Δ*S*, transition temperature *T*_1/2_, and occurrence of hysteresis, DSC measurements down to liquid-nitrogen temperatures are sufficient in most cases [91–94].

#### Diffraction Methods

The rearrangement of the valence electrons among the antibonding e_g _*orbitals* and the slightly bonding t_2g_ orbitals (in the case of octahedral symmetry) during thermal ST is always associated with more or less strong changes in the crystal lattice. Continuous spin transitions only ever show changes in metal–ligand bond angles and lengths (*displacive* transitions), but do not display changes in the space groups. Discontinuous spin transitions with hysteresis in the ST curve *γ_HS_*(*T*) curves typically occur alongside a change in the space group, i.e., a crystallographic phase change of first order with a major reorganization of the lattice *(reconstructive* transitions). Thus, it is obvious that the determination of crystal structure at various temperatures around the ST temperature *T*_1/2_ is highly desirable in studies of ST phenomena in solids. Even temperature-dependent X-ray powder diffraction data from temperature-dependent studies can be extremely useful in the characterization of the type of ST (continuous or discontinuous) and for determining changes of the lattice parameters [95].

Extensive structural investigations have been carried out on many SCO compounds, mostly by X-ray diffraction [18,22,96–99]. As expected, one has found that in all cases the metal–ligand bond length is longer in the HS state than in the LS state due to the above-described charge reorganization. The change of metal–ligand bond lengths is particularly large for SCO compounds of iron(II), viz. ca. 10% (Δ*r*_HL_ = *r*_HS_ – *r*_LS_ ~ 200–220 pm). This generally causes a change of 3–4% in elementary cell volumes [22,99–100]. For comparison, bond-length changes in octahedral iron(III) SCO compounds, also with Δ*S* = 2 transitions, are less dramatic with only Δ*r*_HL_ = 10–13 pm, because the LS state contains one electron hole in the t_2g_ orbitals as can be seen from the electron configurations t_2g_^5^e_g_^0^ (^2^T_2g_, LS)↔t_2g_^3^e_g_^2^ (^6^A_1g_, HS). Δ*r*_HL_ is even less pronounced in cobalt(II) SCO compounds (Δ*r*_HL_ ≤ 10 pm) with Δ*S =* 1 transitions between the electron configurations t_2g_^6^e_g_^1^ (^2^E_g_, LS)↔t_2g_^5^e_g_^2^ (^4^T_1g_, HS), because only one electron is transferred from antibonding e_g_^*^ to t_2g_ orbitals. The size of Δ*r*_HL_ is important for the build-up of cooperative interactions.

Structural investigations of SCO compounds at variable temperatures are of outmost importance for the development of theoretical approaches [23–24,101–102] to understand the detailed steps in ST processes in solids. For this purpose it is extremely helpful that with single-crystal structure studies one can unravel the structures of existing networks of hydrogen bonding or specific orderings of π-stacking. Although they are not a prerequisite for thermal ST to take place, such features take part or at least aid in the cooperative interactions involved in the spin transition. This has been demonstrated experimentally, e.g., by examining the effect of ^1^H/D or ^14^N/^15^N isotope exchange on the properties of the *γ*_HS_(*T*) conversion curves [103].

#### Other physical methods for spin crossover studies

The techniques described in the above sections are commonly employed in most SCO studies. Other techniques are used in special cases, e.g., where further valuable information about a SCO phenomenon can be obtained, which is otherwise not accessible, or where certain material properties (size, susceptibility) require special instrumentation or sample handling. A brief survey of such methods employed less frequently is given in the following.

***X-ray absorption spectroscopy**** (XAS*) can help in obtaining structural information in cases where the material under study is highly dispersed and no single crystal is available. It is usually carried out in two variants: X-ray absorption near edge structure (XANES) and extended X-ray absorption fine structure (EXAFS). XANES provides information essentially about molecular geometry and oxidation states. EXAFS yields information about very local electronic and molecular properties, such as coordination number, ligand denticity, and isomerism [104]. In addition, structures have been derived from EXAFS spectroscopy, e.g., for iron(II)-1,2,4-triazole polymeric chain compounds [105–109], which, however, is possible in some cases only by comparative studies of analogous Cu compounds.

***Positron annihilation spectroscopy*** (PAS) was first applied to investigate the SCO compound [Fe(phen)_2_(NCS)_2_] [110] and later on to study the SCO behavior of single crystals of [Fe(ptz)_6_](BF_4_)_2_ and zinc-diluted mixed crystals thereof [111]. Although one has observed changes of specific PAS parameters (Doppler broadening of the annihilation peak, lifetime of the ortho-positronium) in different spin states, the technique will probably not attain the status of a widely employed method in SCO research.

***Magnetic resonance spectroscopy**** (NMR, EPR).* Proton NMR measurements (Evans method) have been carried out to follow a temperature-dependent ST phenomenon in solution [82,112–117]. The paramagnetic peak changes position with varying temperature relative to a standard reference signal, which in favorable cases can be part of the SCO compound (e.g., the anion) [113–118], and this reflects the ST process. In studies of the solid state, however, the NMR spectrum of SCO compounds has been of little value. The various different protons typically present in the ligand molecules give rise to broad lines, which are difficult or impossible to analyze.

EPR spectroscopy has been more successfully employed in SCO research than has NMR. SCO compounds of iron(III), iron(II), and cobalt(II), which are the 3d transition metal elements most actively studied with EPR, typically reveal characteristic spectra that are sufficiently well resolved in both HS and LS states. There is no spin–orbit coupling in SCO compounds of iron(III) in the HS (*^6^*S) state and, hence, the relaxation times are long. EPR signals appear at characteristic *g* values and provide information about characteristic parameters of the zero-field-splitting (ZFS), *D* for axial and *E* for rhombic distortions. In the LS state of iron(III) (^2^T_2_) spin–orbit coupling does occur, but at low temperatures the vibrations are slowed down and electron–phonon coupling becomes weak and therefore relaxation times are long, and hence, the EPR spectrum of the LS state of iron(III) exhibits a single line near to *g* ~ 2, typical for the presence of one unpaired electron, for a polycrystalline sample. In a single-crystal study, anisotropy effects usually play an important role and can be observed through the *g*-value components *g_x_*, *g_y_*, *g_z_*. EPR spectroscopy is a highly valuable tool to decipher the structural information of a SCO system, which is otherwise barely or not at all accessible.

EPR spectroscopy of paramagnetic iron(II) is impossible at higher temperatures, because spin–orbit coupling within the ^5^T_2_ state produces short spin-lattice relaxation times. EPR spectra can only be measured at 20 K or lower, at which the relaxation times are longer due to a slowing down of the vibrations. The resolution of EPR signals can be improved significantly on doping of the Fe(II) SCO complex with appropriate EPR probes, such as Mn(II) or Cu(II), as was reported first by McGarvey et al. [119] with [Fe(phen)_2_(NCS)_2_] and [Fe(2-pic)_3_]Cl_2_·EtOH (2-pic = 2-picolylamine) doped with 1% Mn(II) and later by Haasnoot et al. [120] with [Fe(btr)_2_(NCS)_2_]·H_2_O (btr = 4,4’-bis-1,2,4-triazole) doped with ca. 10% Cu(II). Employing this technique, McGarvey et al. also measured excellent quality EPR spectra of single crystals of [Fe(ptz)_6_](BF_4_)_2_ doped with 1% Mn(II) [121]. They determined the *D* and *E* values for both spin states and verified the existence of two structurally different LS phases, which are formed by fast or slow cooling. This method of doping SCO systems with Cu(II) and Mn(II) as EPR probes has proven to be very informative and easy to apply to spin crossover studies [122–126].

***Muon spin relaxation**** (μSR)* has only recently been applied to the study of SCO in iron(II) complexes [127–140], while this technique is widely used for the study of organic radicals [141–142]. Spin-polarized positive muons *μ^+^* implanted into a sample act as probes of local magnetization. Information about magnetic fluctuations is obtained in a time window covering the range of 10^−9^–10^−5^ s, which complements the time windows of other physical techniques for studying dynamic phenomena [143–148]. The thermally induced SCO in [Fe(ptz)_6_](ClO_4_)_2_ was followed by recording the temperature dependence of the initial asymmetry in zero field, *a_0_* [129–130]. This measurement reflected the fraction of muons interacting with unpaired spins and, thus, the fraction of HS molecules at any temperature [135–136]. The possibility of investigating spin fluctuation rates was established with the classical iron(II) complex [Fe(phen)_2_(NCS)_2_], polymorph I [135–136]. In another case, fast dynamics have been revealed in the HS regime of the ST coordination polymer [Fe(NH_2_trz)_3_](NO_3_)_2_ (NH_2_trz = 4-amino-1,2,4-triazole) [138–139].

***Nuclear resonance scattering of synchrotron radiation**** (NRS)* began with the pioneering work of Gerdau et al. in 1985 [71–73] who proposed an unconventional Mössbauer spectroscopy technique based on the possibility of using synchrotron radiation for nuclear resonance experiments in two ways, i.e., *nuclear forward scattering (NFS) and nuclear inelastic scattering (NIS)* of synchrotron radiation. Whereas conventional Mössbauer spectroscopy can be considered as “spectroscopy in the energy domain”, NFS has been successfully employed as a time-differential technique. NFS allows one to study hyperfine interactions to obtain the Mössbauer parameters isomer shift, electric quadrupole and magnetic dipole splitting, as can be obtained by conventional Mössbauer spectroscopy, albeit with much smaller samples and shorter measuring times. Of even greater importance, Mössbauer isotopes that have not been accessible with conventional Mössbauer spectroscopy can be used. NIS allows the investigation of vibrational modes and the partial density of states (PDOS) locally, i.e., in the close neighborhood of the Mössbauer probe nucleus. Compared, for instance, to Raman spectroscopy, NIS allows measurements with higher resolution without perturbation from surrounding vibrations.

### Light-induced spin transition in iron(II) compounds

An unusual photophysical phenomenon was discovered with the SCO compound [Fe(ptz)_6_](BF_4_)_2_ by the Mainz group [57] in 1984. [Fe(ptz)_6_](BF_4_)_2_ undergoes thermal ST as clearly demonstrated by the temperature-dependent Mössbauer spectra shown in Figure 4 as well as the color change of a single crystal and the optical spectra depicted in Figure 3. The phase change occurs at a ST temperature *T*_1/2_ of ca. 135 K with hysteresis of ca. 7 K width [58–59]. In a temperature-dependent study of the optical spectra of this compound it was observed for the first time that a LS to HS conversion in a solid SCO compound can also be induced by irradiating the crystals with (preferentially green) light, leading to a metastable long-lived HS state, which can have very long lifetimes on the order of days at temperatures below ca. 20 K [57,59]. This photophysical phenomenon has been termed *Light-Induced Excited Spin State Trapping* (LIESST).

**Figure 6:**
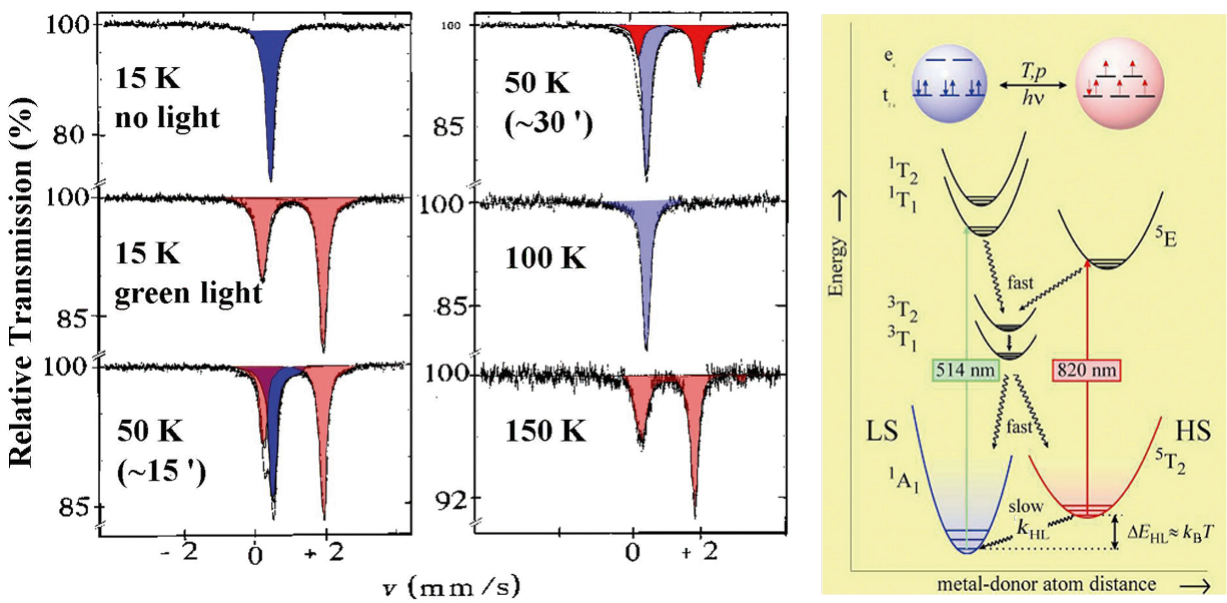
(left) Demonstration of light-induced spin state trapping (LIESST) in [Fe(ptz)_6_]BF_4_)_2_ with ^57^Fe Mössbauer spectroscopy (for details see text). (right) Jablonski diagram showing the photophysical processes of the LIESST and reverse-LIESST effects [23]; details are described in the text (Reproduced with permission from [36]. Copyright 2012 Wiley-VCH Verlag GmbH & Co. KGaA).

Figure 6 recapitulates the discovery of the LIESST phenomenon [60]. A single crystal of [Fe(ptz)_6_](BF_4_)_2_ (size ca. 3 × 3 cm^2^) was mounted in a cryostat for optical spectroscopy at variable temperatures down to that of liquid helium. The crystal is white in the HS phase and displays only a weak absorption band at 12000 cm^−1^ of the spin-allowed but parity-forbidden ^5^T_2_→^5^E transition. At 80 K, the crystal changed to the LS state by thermal SCO and the ^5^T_2_→^5^E transition is no longer observable. In the LS state the crystal is red and absorbs (more strongly than in the HS phase due to stronger oscillator strength) at 18000 and 26000 cm^−1^ arising from the spin-allowed LS absorption bands ^1^A_1_→^1^T_1_ and ^1^A_1_→^1^T_2_. Irradiation of the crystal at ca. 10 K with green light (514 nm from Ar^+^ laser) leads to convertion of the LS state to the metastable HS state. The optical spectrum of the white spot (ca. 1 mm in diameter) is virtually identical to that measured at 300 K.

Mössbauer spectroscopy is ideally suited to follow the light-induced spin state conversion, as demonstrated for [Fe(ptz)_6_]BF_4_)_2_ in Figure 6. The advantage of using Mössbauer spectroscopy is that the sample under study may be polycrystalline material; it does not require a single crystal as with optical spectroscopy. The ^57^Fe Mössbauer spectra shown in Figure 6 were recorded with a polycrystalline sample of [Fe(ptz)_6_](BF_4_)_2_, which was first cooled to 15 K, at which the sample is in the LS state (upper left). Irradiating the sample with green light (ca. 500 nm from an argon ion laser) at 15 K converts the LS state (resonance signal shown in blue) quantitatively to the long-lived metastable HS state (middle left, quadrupole doublet shown in red). Thermal relaxation occurs at 50 K on a 15 minute timescale; the HS doublet disappears in favor of the reappearance of the thermodynamically stable LS state (lower left and upper right: the sample was heated for 15 minutes at 50 K and then cooled down quickly to the measuring temperature of 15 K in two runs). As indicated by the spectrum shown in Figure 6 (middle right), thermal relaxation of the metastable HS (LIESST) state to the stable LS state is complete at 100 K. Further heating to 150 K converts the sample thermally at ca. 135 K from the LS to the HS state.

The LIESST phenomenon is well understood on the basis of ligand field theory [23,149]. The photophysical processes involved in the LIESST phenomenon are depicted in the Jablonski diagram in Figure 6 (right). Note that the two potential wells for the LS and HS ground states are displaced due to the fact that the metal-ion–donor-atom distance in the HS state is weaker and therefore longer compared to the stronger and therefore shorter bond distance in the LS state (Figure 1). Green light (514 nm from an Argon ion laser) excites the LS state (^1^A_1_) by the spin-allowed but parity-forbidden transition to the ^1^T_1_ and ^1^T_2_ ligand field states. These decay fast in two consecutive intersystem crossing processes, to the spin triplet states ^3^T_1,2_ and finally to the ^5^T_2_ state. Two factors are favorable for the occurrence of LIESST: (i) The spin triplet states ^3^T_1,2_ are placed energetically lower than the ^1^T_1_ and ^1^T_2_ ligand field states; (ii) The *double intersystem crossing* decay path is favored by spin–orbit coupling and therefore faster than the direct decay path back to ^1^A_1_. The decay of the ^5^T_2_ state to the ^1^A_1_ state is highly spin- and parity-forbidden; the long-lived metastable HS state is trapped until radiationless thermal relaxation (heat is transferred to lattice vibrations) sets in by nonadiabatic multiphonon processes [23,149]. Light-induced back conversion (reverse-LIESST) of the metastable LIESST state is effected by irradiating the sample with red light (820 nm from a Krypton laser), whereby the metastable ^5^T_2_ state (“LIESST state”) is excited to the ^5^E_g_ state, which decays fast – again favored by spin–orbit coupling – by double intersystem crossing via ^3^T_1,2_ states to the LS (^1^A_1_) state [149]. The LIESST effect has mostly been observed with SCO compounds of iron(II), but a few examples of SCO compounds of iron(III) exhibiting LIESST have recently been reported [150].

The HS→LS relaxation kinetics of decaying metastable LIESST states was examined experimentally by Hauser [151]. He interpreted the results on the basis of a nonadiabatic multiphonon relaxation model proposed earlier by Buhks, Jortner et al. [152]. As an essential feature, it was found that the lifetime *t*_HL_^0^, i.e., the low temperature tunneling rate *k*_HL_^0^ = (*t*_HL_^0^)^−1^ of the LIESST state, is governed by the energy difference Δ*E*_HL_^0^ between the lowest vibronic energy levels of the HS and LS states involved; this has become known as *inverse energy gap law*. The energy gap Δ*E*_HL_^0^ increases with increasing ligand field strength. This means that, at comparable temperatures, the metastable LIESST state lives longer the weaker the ligand field strength is [149,151]. Typical lifetimes of photoexcited LIESST states in SCO complexes have been found to be on the order of minutes to hours, or even days, below ca. 20 K. In the case of LS complexes with considerably stronger ligand field strength, LIESST states decay much faster (within micro- to nanoseconds). However, an exception was reported for which the authors observed that in the case of the system [Fe_0.02_Mn_0.98_(terpy)_2_](ClO_4_)_2_, where 2% of ^57^Fe enriched [^57^Fe(terpy)_2_]^2+^ complex molecules embedded in the host matrix of the corresponding Mn compound are in the LS state even in the room-temperature region, the inverse-energy-gap law was not obeyed. In this case mean lifetimes of the LIESST state in photo-excited [^57^Fe(terpy)_2_]^2+^ complex molecules with LS behavior were expected to be roughly on the order of microseconds at 20 K or below [153]. Surprisingly, the measurements yielded lifetimes on the order of several days. The authors have named this phenomenon the *strong-field LIESST* effect.

Later it was found that photo-induced excited spin state trapping similar to LIESST is also possible with hard X-rays as the excitation source [154]. Moreover, *light-induced perturbation of thermal hysteresis* (LIPTH) was observed as a possible consequence of LIESST in a SCO compound of iron(II) [155].

Zarembowitch and Boillot et al. [156–157] have proposed and successfully verified another strategy, denoted as *ligand-driven light-induced spin change* (LD-LISC), to induce spin state switching in iron(II) complexes. The primary step of LD-LISC is a photochemical reaction on the ligand molecules, for instance a photo-induced *cis*/*trans*-isomerization on 4-styrylpyridine (stpy) as a ligand, e.g., in [Fe(II)(stpy)_4_(NCS)_2_], which modulates the ligand field strength at the metal center and eventually leads to spin state switching. The occurrence of the LD-LISC effect was observed in several iron(II) and iron(III) complexes. On changing the composition of the sample, the working temperature and excitation wavelengths are modulated so that the effect may be observed at room temperature upon irradiation of the sample with visible light. Experiments so far were performed on compounds either in solution or embedded in polymeric matrices.

### Effect of pressure on thermal spin crossover

**Figure 7:**
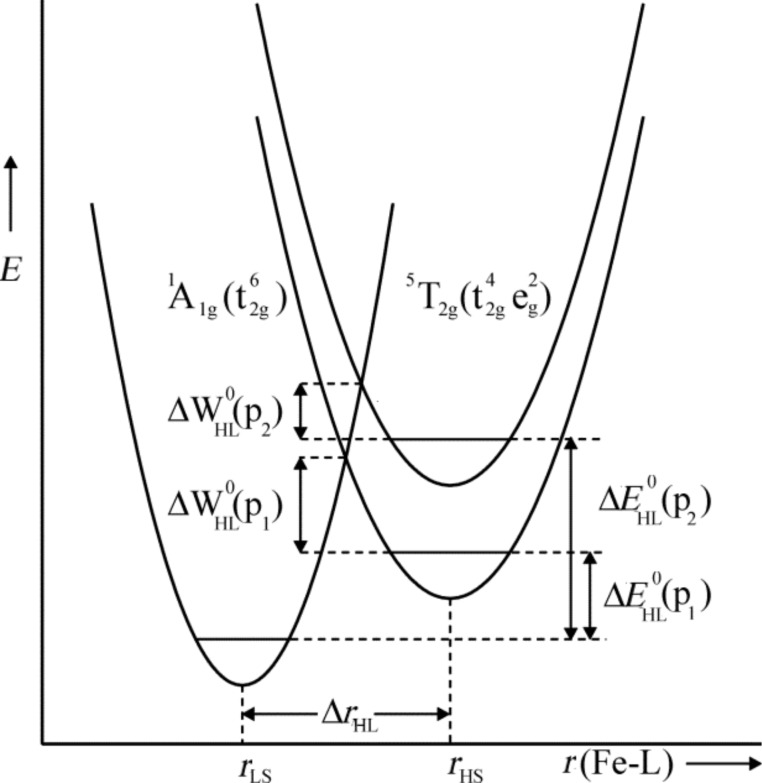
Schematic representation of the pressure influence (p_2_ > p_1_) on the LS and HS potential wells of an Fe(II) spin crossover compound (Reproduced with permission from [159]. Copyright 2005 Elsevier B. V.).

Application of pressure shortens the metal–donor-atom distances of SCO complex molecules and increases the ligand field strength at the metal center. It is therefore expected that application of pressure favors the LS state. This was already experimentally confirmed by Ewald et al. in their studies of pressure effects on solutions of iron(III) complexes [158]. They found that the temperature dependent thermal equilibrium between ^2^T_2_**↔**^6^A_1_ was shifted under pressure in favor of the LS state ^2^T_2_*.* It is indeed the large difference in the metal–donor-atom bond lengths, Δ*r*_HL_ = *r*_HS_ – *r*_LS_ ≈0.1 and 0.2 Å, for Fe(III) and Fe(II) SCO molecules, respectively, that stabilizes the LS state under application pressure. Figure 7 schematizes the effect of pressure on the SCO behavior in the case of SCO complexes of iron(II).

Application of pressure (p_2_ > p_1_) increases the relative vertical displacement of the potential wells and thus stabilizes the LS state; the additional minor decrease of the relative horizontal displacement of the potential wells under pressure is neglected in this picture. As a consequence, the zero-point energy difference Δ*E*^0^_HL_ increases by the work term *p*Δ*V*^0^_HL_, which in turn decreases the activation energy Δ*W^0^*_HL_ and finally favors the LS state.

Extensive high-pressure experiments on SCO compounds by using diamond anvil cells have been reported by H. G. Drickamer [160] and later by other research groups [161–163]. One has realized that the SCO properties are very susceptible to the application of pressure and that it is more favorable and meaningful for studies of the mechanisms of thermal ST and LIESST state relaxation in solids, to perform such experiments with hydrostatic pressure cells working in relatively low pressure regions (up to ca. 1.5 GPa). Such experiments were first carried out by using hydrostatic gas pressure cells in conjunction with Mössbauer spectroscopy [97,164] and optical spectroscopy [165]. More recently, special hydrostatic pressure cells for magnetic and Mössbauer measurements have been developed, in which silicon oil is used as the pressure-transmitting medium [166–167].

The influence of pressure on SCO properties, for instance the critical ST temperature and shape and position of hysteresis loops (condition for bistability), has potential for practical applications, e.g., as a pressure sensor in remote locations. ST curves γ_HS_(*T*) or, more often used, *χ*_M_*T* (*χ*_M_ = molar magnetic susceptibility) are recorded as a function of the applied pressure. From a series of such curves a calibration plot *γ*_HS_(*p*)_T_ or (*χ*_M_*T*)(*p*)_T_ can be constructed, preferentially at room temperature. The ST curves at different pressures may be recorded with a convenient method, e.g., magnetic, optical or Mössbauer measurements. Although the primary focus here is on studies of pressure effects on SCO coordination compounds, we should like to point out that similar studies have been carried out by several research groups on oxidic phases of transition elements in natural materials, e.g., in the earth’s mantle, as well as in synthetic materials, and it has been found that indeed SCO phenomena may occur in such compounds under the influence of pressure [168–172]. Of the many studies of pressure effects on SCO compounds carried out so far [23,160,168–175], only a few selected examples will now be discussed.

**Figure 8:**
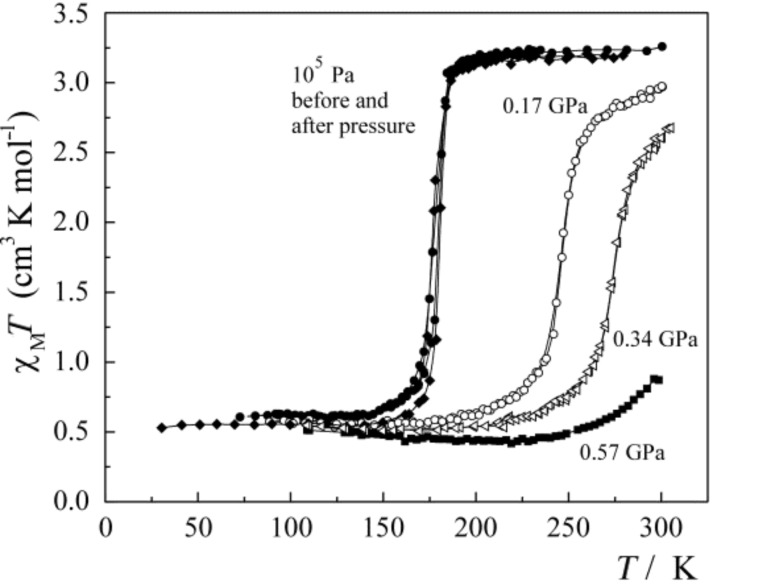
*χ*_M_*T* versus *T* curves at different pressures for [Fe(phen)_2_(NCS)_2_], polymorph II. (Reproduced with permission from [159]. Copyright 2005 Elsevier B. V.).

In Figure 8 are plotted the *χ*_M_*T* versus *T* curves of [Fe(phen)_2_(NCS)_2_] (Polymorph II) measured at different pressures [176]. Thermal ST at ambient pressure occurs near 175 K. With increasing pressure the transition curves are shifted to higher temperatures, due to stabilization of the LS state, and become slightly more gradual. At the highest pressure applied in this study, viz. 0.57 GPa, the paramagnetic HS state is nearly suppressed in the room-temperature region. On release of pressure, the ST behavior is practically the same as before under ambient pressure.

**Figure 9:**
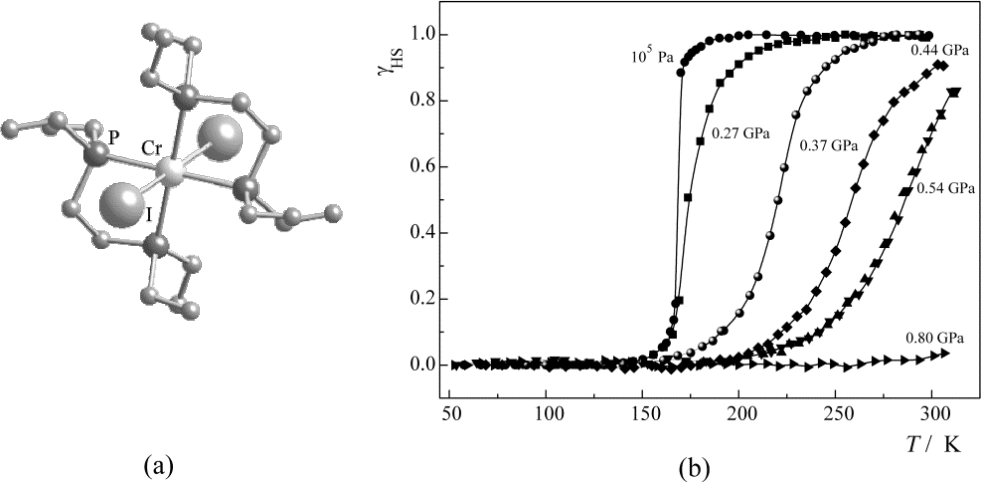
Molecular structure (a) and γ_HS_(*T*) curves at different pressures for [CrI_2_(depe)_2_] (b) (Reproduced with permission from [159]. Copyright 2005 Elsevier B. V.).

Very similar results were obtained from a pressure-effect study on the SCO compound [Cr(II)I_2_(depe)_2_] (depe = diethylphosphino-ethane), which undergoes at ambient pressure a sharp ^3^T_1g_ (S = 1)↔^5^E_g_ (S = 2) thermal ST at 169 K [176]. In this case, the ST curves become considerably more gradual with increasing pressure than in the former case but are also shifted to higher temperatures until, at the highest pressure applied, the HS (S = 2) state has entirely been suppressed in the room-temperature region (Figure 9).

**Figure 10:**
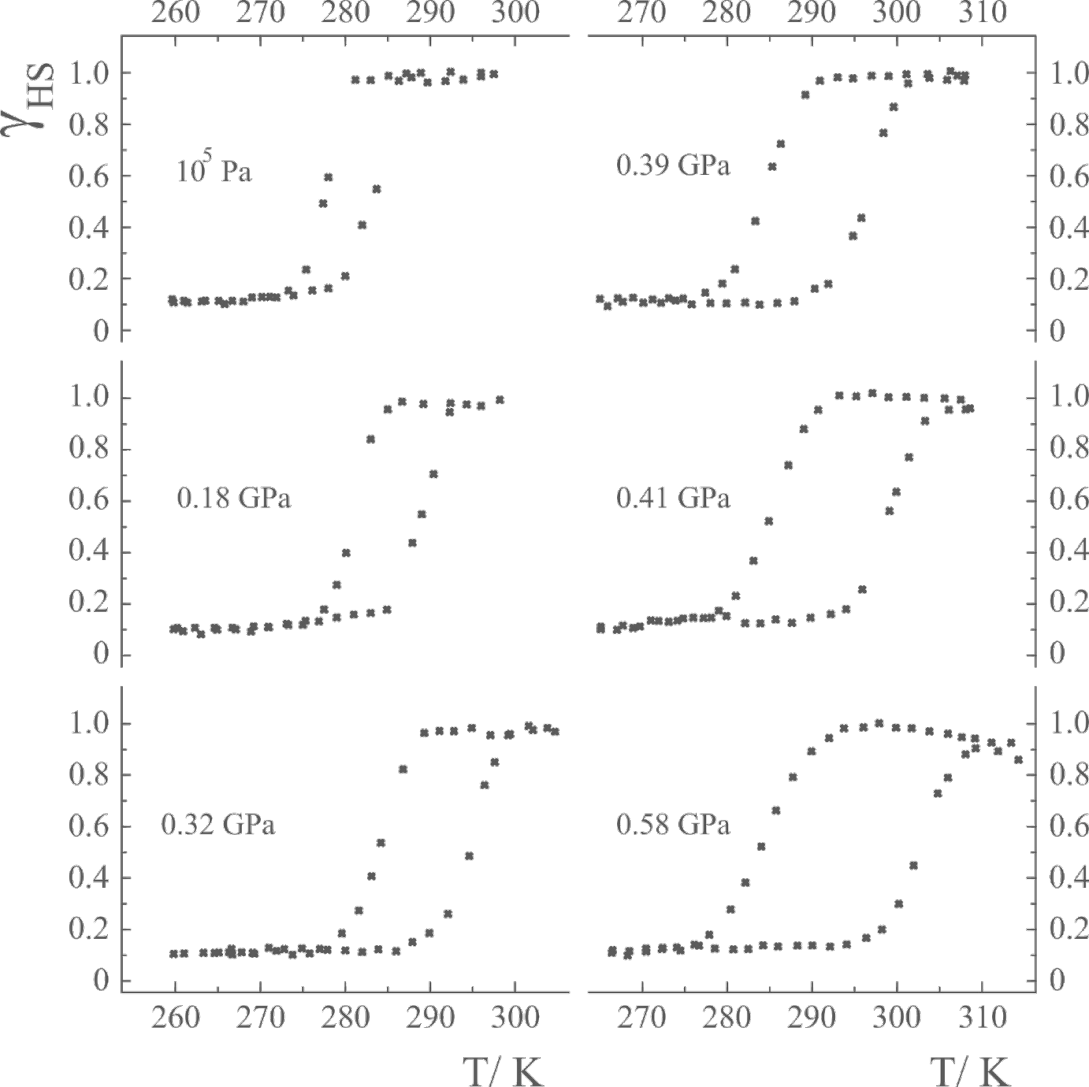
HS molar fraction *γ*_HS_ versus*T* at different pressures for [Fe(phy)_2_](BF_4_)_2_. The hysteresis loop broadens with increasing pressure. (Reproduced with permission from [167]. Copyright 1999 Elsevier B. V.).

Figure 10 shows the results of a pressure-effect study on [Fe(phy)_2_](BF_4_)_2_ (phy = 1, 10-phenanthroline-2-carbaldehydephenylhydrazone) [167]. This system exhibits hysteresis during the thermal ST. The occurrence of hysteresis is known to be a prerequisite for bistability, i.e., within the hysteresis loop the compound shows two different *γ*_HS_(*T*) values at the very same temperature, one each on temperature decrease and increase. It is clearly seen that the hysteresis loop is sensitive to the application of pressure.

**Figure 11:**
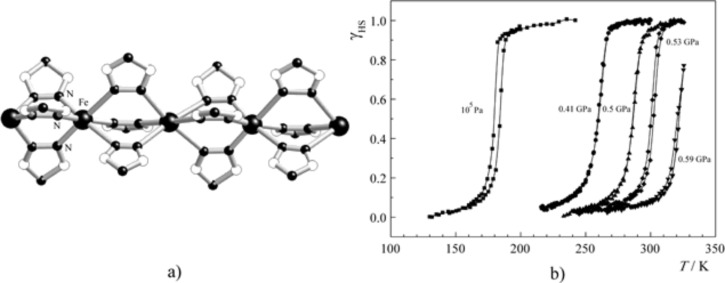
Proposed structure of the polymeric [Fe(4R-1,2,4-triazole)_3_]^2+^ spin crossover cation (a) and plot of *γ*_HS_ versus *T* at different pressures for [Fe(hyptrz)_3_](4-chlorophenylsulfonate)_2_·H_2_O (b). (Reproduced with permission from [159]. Copyright 2005 Elsevier B. V.).

In addition, polymeric SCO compounds of iron(II) have been comprehensively studied [177–180]. An example is given by the system [Fe(hyptrz)_3_](4-chlorophenylsulfonate)_2_·H_2_O, in which hyptrz = 4-(3’-hydroxpropyl)-1,2,4-triazole, which displays a very steep and complete ST around 180 K with thermal hysteresis of 6 K in width as supported by magnetic susceptibility measurements at ambient pressure (Figure 11b) [178]. Application of pressure causes a shift of the transition curve to room temperature and even higher but retains the original steepness and shape. The sharp HS→LS transition is accompanied by an easily detectable color change from white (HS) to deep purple (LS). This material holds the potential for application as a pressure sensor [181].

### Selected spin crossover compounds of iron(II)

As stated above, SCO research began around 1930 with the first observations of thermally induced spin state conversion in iron(III) compounds [1–6]. Exploration of this field was intensified ca. thirty years later with the first documentations of thermal SCO phenomena in iron(II) compounds [7–15]. With the advent of Mössbauer spectroscopy in the early sixties an excellent microscopic tool became available for the characterization, predominantly of iron compounds, regarding their valence electron structure and the resulting physical properties. In many cases this hyperfine interaction technique has enabled one to gain insight into details of SCO phenomena that are not accessible with other techniques.

The early observations of thermal SCO phenomena were all made on mononuclear compounds of iron(III) [1–6] and iron(II) [7–13]. One had soon realized that the SCO behavior, i.e., the shape of the ST function *γ*_HS_*(T)*, as sketched in Figure 2, may change considerably from one compound to another and that this may be largely caused by cooperative interactions between the spin state changing complex molecules. From then on up to the present stage, cooperative interactions have been one of the most important aspects in SCO research. Questions concerning the creation, nature, strength and mechanism of cooperative interactions have been the main objectives of nearly all experimental and theoretical SCO projects. Work has been performed on a large variety of SCO materials, from simple mononuclear and oligonuclear compounds with zero-dimensional lattices, to polymeric systems with 1D, 2D and 3D lattices. In the case of mononuclear SCO compounds various influences on the SCO behavior have been studied in detail. Examples will be described in the next section.

#### Influences on SCO behavior in mononuclear SCO compounds of iron(II)

Mononuclear SCO compounds with zero-dimensional lattices, such as the classical SCO compound [Fe(II)(phen)_2_(NCS)_2_], the picolylamine complexes [Fe(II)(2-pic)_3_]X_2_·Sol (X = Cl, Br, I; Sol = EtOH, MeOH, H_2_O, 2H_2_O) and the alkyltetrazole complexes of iron(II), e.g., [Fe(II)(ptz)_6_](BF_4_)_2_, have been extensively studied regarding chemical and physical influences on the SCO behavior ([23] and references therein).

***Replacement of ligand****:* An example for the effect of replacement of a ligand in the complex molecule is shown in Figure 5. The compound [Fe(phen)_3_]X_2_ is in the LS state, because the ligand field strength is too strong to meet the critical SCO condition. Replacement of one bifunctional phen ligand by two monofunctional NCS^−^ ligands weakens the ligand field strength sufficiently to induce a thermal spin transition.

***Intraligand substitution****:* A similar effect may be observed when the ligand field strength is modulated by substitution inside the ligand molecule. This has been illustrated with the group of [Fe(Y-phen)_3_]X_2_ complexes [182]. Exchange of H for CH_3_ in either the 2- or 9-position of the three phen ligands weakens the ligand field strength due to steric hindrance (whereby the metal–donor-atom distance is elongated) and the LS behavior of [Fe(phen)_3_]X_2_ turns to SCO behavior of the tris(2-CH_3_-phen) complex. If both the 2- and 9-positions of the three phen ligands are substituted by CH_3_ the steric hindrance is even stronger and weakens the ligand field strength further, yielding HS behavior of the tris[2,9-(CH_3_)_2_] complex down to very low temperatures. It was found that a combination of steric hindrance due to bulkiness and an electronic influence of the substituent on the basicity of the coordinating N-atom is responsible for the influence on the SCO behavior. The paramagnetic property of the complex (given by the molar fraction of HS molecules, *γ_HS_*, at a given temperature), increases in the order Y = H < CH_3_O < CH_3_ < Cl. One has also found that a change of the substituents at positions not adjacent to the coordinating N-atom in the phen ligand does not influence the spin state in comparison to the unsubstituted [Fe(phen)_3_]X_2_ complex.

**Figure 12:**
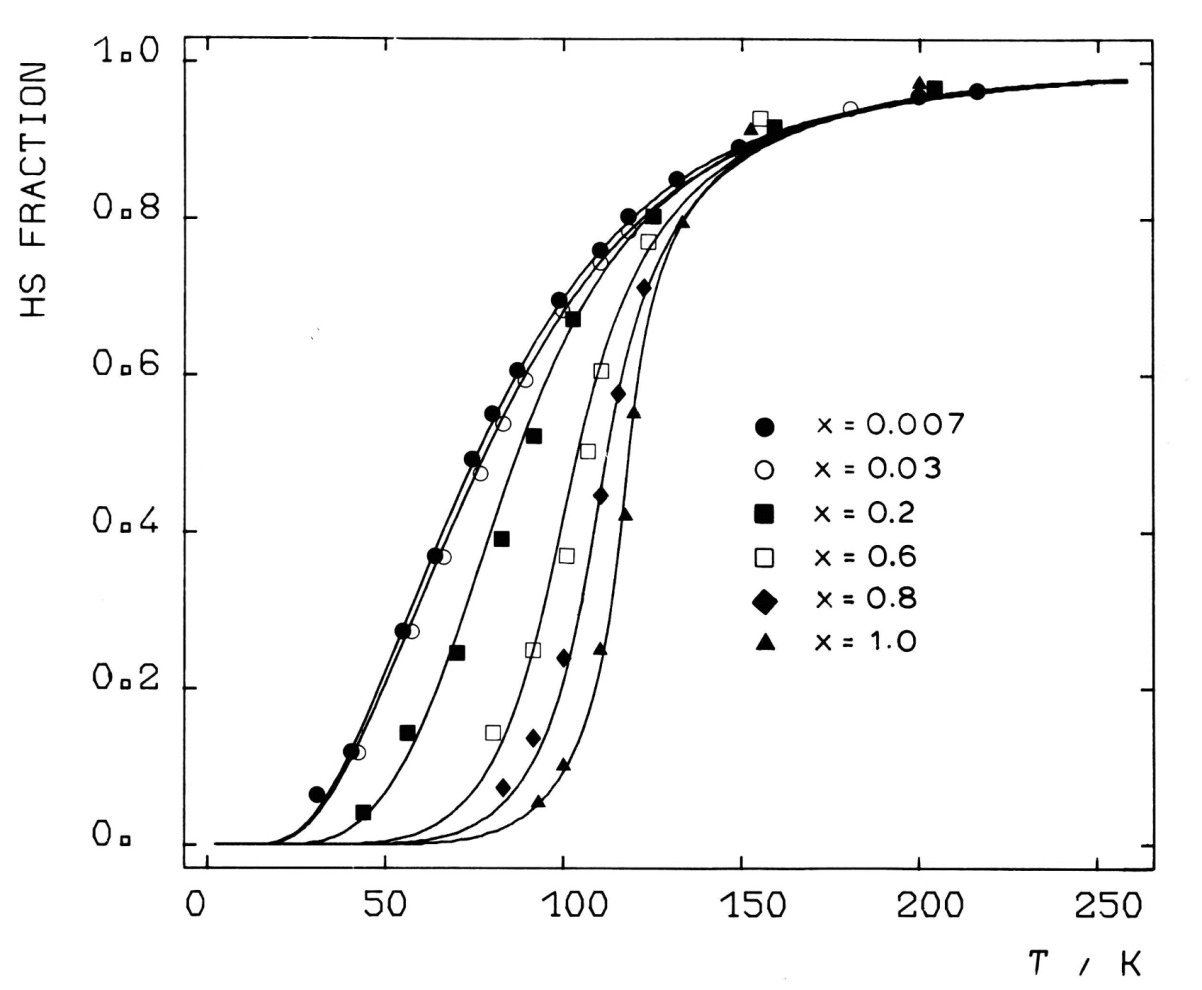
Temperature dependence of the HS fraction *γ*_HS_(*T*), determined from Mössbauer spectra of [Fe(II)_x_Zn_1-x_(2-pic)_3_]Cl_2_·EtOH for different iron concentrations x. The ST curves become more gradual with increasing x due to weakening of the strength of the cooperative interactions. (Reproduced with permission from [27]. Copyright 2003 Wiley-VCH Verlag GmbH & Co. KGaA).

***Influence of metal dilution****:* The occurrence of thermal SCO in the picolylamine complexes [Fe(II)(2-pic)_3_]X_2_·Sol (X = Cl^−^, Br^−^, I^−^; Sol = EtOH, MeOH, H_2_O, 2H_2_O) was first reported by Renovitch and Baker [183]. Determination of the crystal structure of [Fe(II)(2-pic)_3_]Cl_2_·EtOH [96–97] above and below the ST temperature pointed to the existence of hydrogen bonding, which was believed to cause, through cooperative interactions, the marked differences in the ST curves *γ*_HS_(*T*) of the halides [183] and, as found later with Mössbauer spectroscopy [184], also in the various solvates. A detailed study of the ST behavior in the mixed-crystal series [Fe(II)_x_Zn_1-x_(2-pic)_3_]Cl_2_·EtOH using Mössbauer spectroscopy showed that the ST curve was strongly influenced by substitution of Fe by Zn (Figure 12) [185]. Zinc was chosen in this “metal dilution” study because it has the same crystal structure as the analogous iron(II) complex. Most important is the result that, with decreasing iron concentration, the ST curves become more gradual and are shifted to lower temperatures, until at very high dilution the ST curve resembles that of a SCO phenomenon occurring in liquid solution, where practically no cooperative interactions exist. Clearly, the existence of cooperative interactions in solid SCO compounds has been proven in this study. The effect of metal dilution was later studied in other SCO complexes [23] and supported the earlier results. These observations were most important for the development of the so-called *Mainz Model* [23,101–102], a theoretical approach based on a pure mechanical “communication” mode between the spin state changing complex molecules featuring elasticity and lattice expansion.

**Figure 13:**
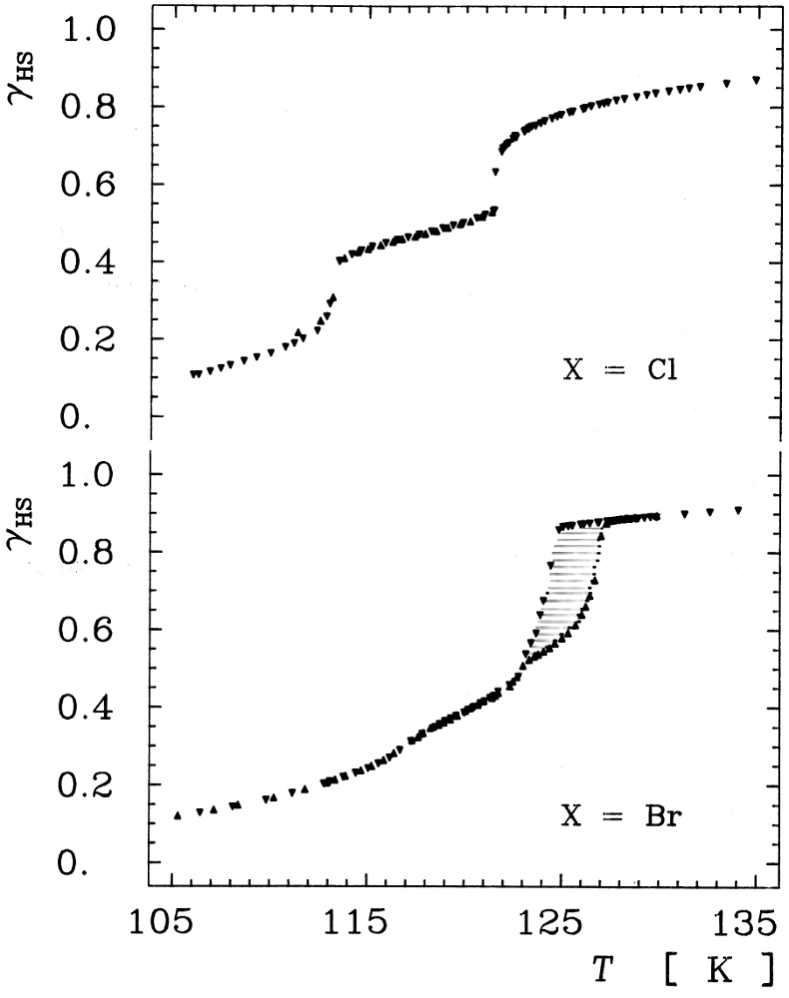
Influence of the noncoordinated anion on the spin transition curve *γ*_HS_(*T*) near the transition temperature *T_1/2_* in [Fe(II)(2-pic)_3_]X_2_·EtOH (X = Cl, Br). (Reproduced with permission from [27]. Copyright 2003 Wiley-VCH Verlag GmbH & Co. KGaA).

***Influence of noncoordinated anions****:* In the case of the SCO compound [Fe(II)(phen)_2_(NCS)_2_] the NCS^−^ anions are coordinated directly to the Fe^2+^ center, i.e., the NCS^−^ anion is in this case a direct codeterminant of the ligand field strength at the metal center. In ionic lattices with cationic SCO complex molecules and uncoordinated counterions in lattice positions remote from the metal center, the anion can nevertheless exercise a strong influence on the SCO behavior through cooperative interactions. Examples are the halides of the series [Fe(II)(2-pic)_3_]X_2_·EtOH for which the ST characteristics was found to depend strongly on the nature of the anion X = Cl^−^, Br^−^, I^−^ (Figure 13) [186–187].

**Figure 14:**
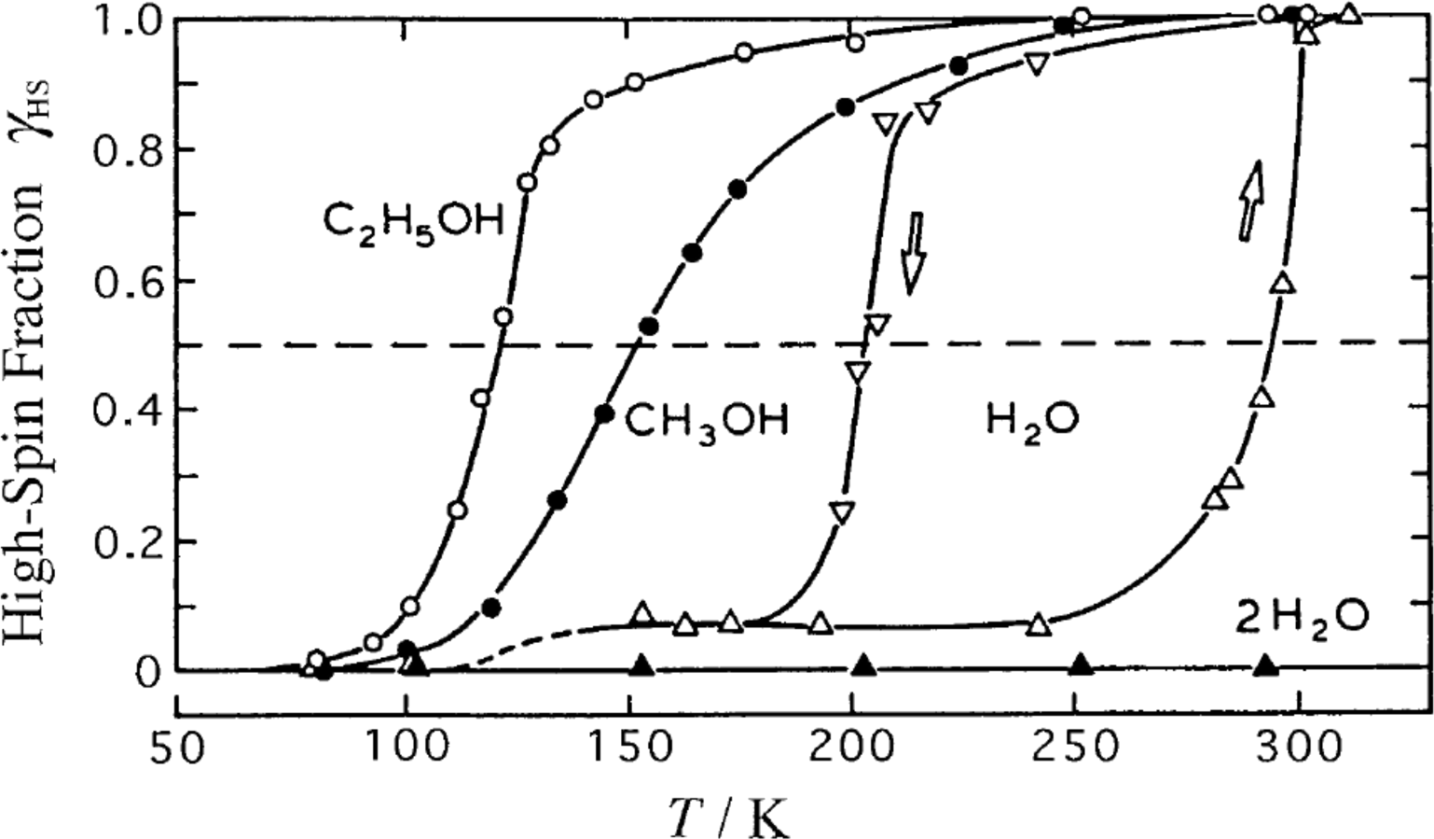
Spin transition curves *γ*_HS_(*T*) for different solvates of the SCO complexes. [Fe(II)(2-pic)_3_]Cl_2_·Solv (Solv = EtOH, MeOH, H_2_O, 2H_2_O) [184]. Differences in the hydrogen bonding network are responsible for the different spin crossover behaviours in these solvates. (Reproduced with permission from [23]. Copyright 1994 Wiley-VCH Verlag GmbH & Co. KGaA).

***Influence of noncoordinated solvents molecules****:* The various solvates of the SCO complexes [Fe(II)(2-pic)_3_]Cl_2_·Solv with Solv = EtOH, MeOH, H_2_O, 2H_2_O were studied with Mössbauer spectroscopy to explore the influence of the noncoordinated solvent molecules on the SCO behavior (Figure 14) [184]. The ethanolate shows a rather steep transition near 115 K; the methanolate shows a more gradual ST near 150 K; the monohydrate exhibits a very broad hysteresis loop with transition temperatures *T_1/2_^↓^* near 200 K and *T_1/2_^↑^* near 290 K; and the dehydrated sample exhibits no ST at all but remains in the LS state. This is clear evidence for the existence of hydrogen bonding as the communication pathways for the cooperative interactions propagating through the lattice.

**Figure 15:**
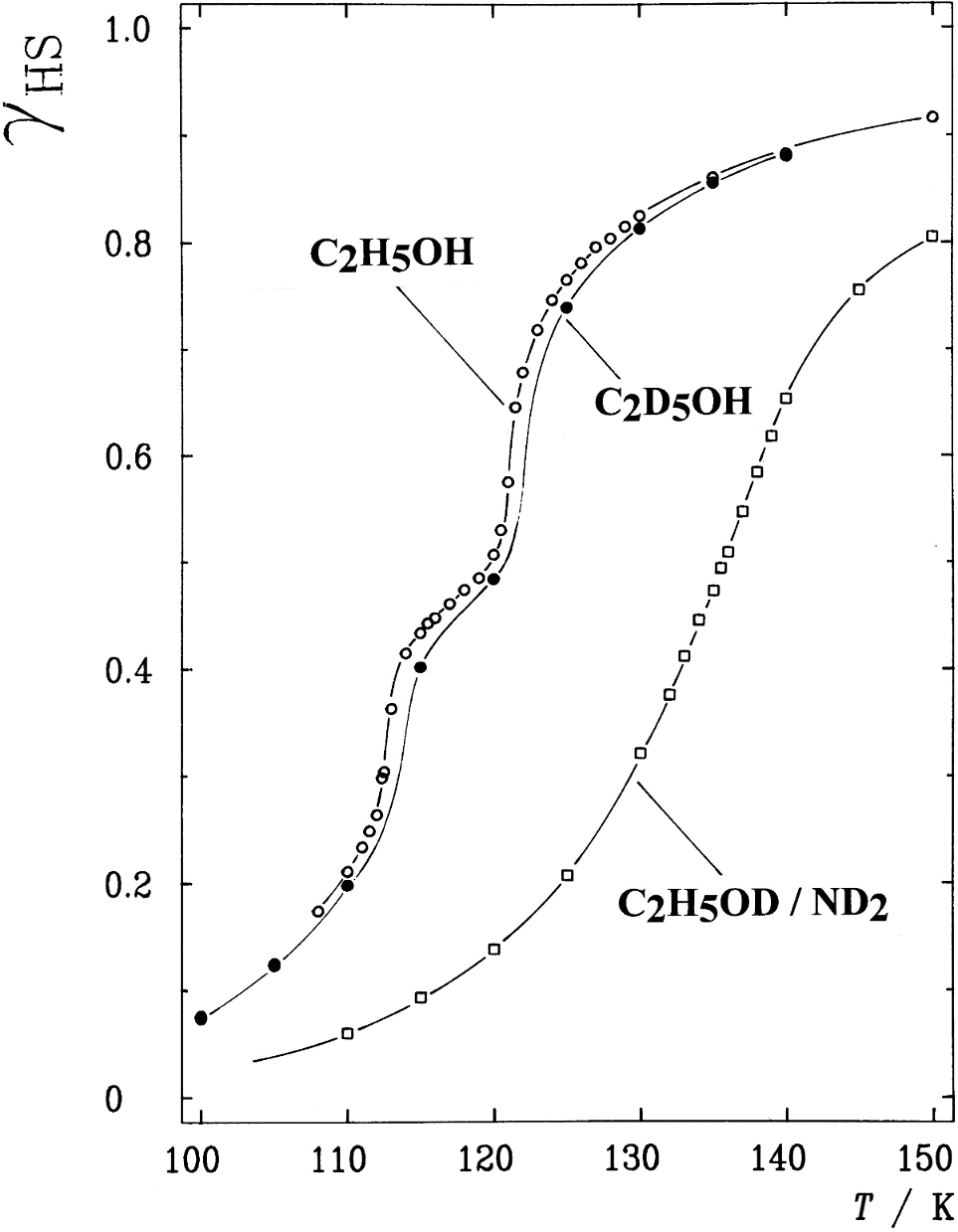
ST curves *γ*_HS_(*T*) of the deuterated solvates of [Fe(II)(2-pic)_3_]Cl_2_·Solv with Solv = C_2_D_5_OH and C_2_H_5_OD/ND_2_ compared with that of the nondeuterated solvate [Fe(II)(2-pic)_3_]Cl_2_·C_2_H_5_OH derived from Mössbauer spectra [103,191]. Deuteration affects the ST behavior in those cases where isotope exchange occurs in positions that are actively involved in the hydrogen-bonding network. (Reproduced with permission from [23]. Copyright 1994 Wiley-VCH Verlag GmbH & Co. KGaA).

***Stepwise spin transition****:* In the early stage of SCO research the observed ST curves have always been of the more or less gradual, continuous type (Figure 2). In the case of the SCO system [Fe(II)(2-pic)_3_]Cl_2_·EtOH it was observed for the first time that thermal ST may also proceed in steps [187]. Earlier studies of this compound with magnetic and Mössbauer measurements always showed a continuous trend of the ST curve *γ*_HS_*(T)* curve in the region of 110–125 K (ca. 50% spin state conversion). A detailed Mössbauer effect study of this region by Köppen et al. revealed that ST in this system occurs in two steps, as is clearly seen in Figure 13 and Figure 15. It was found later by low-temperature crystal structure determination that the stepwise ST in this SCO system is due to an order–disorder phase transition with formation of an intermediate phase [188]. Such stepwise spin transitions have later also been reported for a number of other SCO systems (e.g., [189–190]).

***Isotope effects****:* A Mössbauer spectroscopy study was performed to investigate the influence of deuteration, in various positions of the solvent molecules, on the SCO behavior of [Fe(II)(2-pic)_3_]Cl_2_·Solv (Solv = EtOH, MeOH). Temperature-dependent Mössbauer spectra were recorded of the solvates with Sol = C_2_D_5_OH and C_2_H_5_OD/ND_2_ and compared with that of the nondeuterated ethanolate [Fe(II)(2-pic)_3_]Cl_2_·C_2_H_5_OH. The ST curves for the three solvates are displayed in Figure 15 [97].

The ST curve of the nondeuterated ethanolate shows the “two-step” spin transition first observed in this system [103,191]. The solvate with C_2_D_5_OH is only slightly affected by the deuteration. The reason is that the H/D isotope exchange takes place in a position that is not a constituent of the hydrogen bonding as can be seen from the crystal structure [95–96]. In the case of the system denoted as C_2_H_5_OD/ND_2_, however, deuteration occurs at the OH group, which is a building element of the hydrogen bonding. Simultaneously deuteration occurs also at the acidic NH_2_ group, which is also directly involved in the hydrogen-bonding chain [95–96]. As a consequence, deuteration affects this system much more strongly than in the former case. This is reflected in Figure 15 by a drastic shift of the ST curve to higher temperature and the disappearance of the step. These results clearly underline the importance of hydrogen bonding for cooperative interactions between spin state changing complex molecules. It also evidences that intra- and intermolecular vibrations play an important role in the communication of spin state changes through the lattice. Similar observations have been made for the deuterated methanolate (Solv = CH_3_OH) [103] and also for [Fe(II)(2-pic)_3_]Cl_2_·C_2_H_5_OH with H/D exchange in the CD_2_NH_2_ substituent and at the 3,4,5-positions of the pyridine ring [191]. An isotope effect study was also performed with ^14^N/^15^N exchange at the amino group [191]. Despited the fact that the coordinating N atom is an important constituent of the hydrogen-bonding chain, the ST curve maintained the step in this case and was shifted only slighty to higher temperature, very similar to the effect observed on the C_2_D_5_OH system. A minor influence on the SCO curve is expected in the case of ^14^N/^15^N exchange because of the relatively small change of reduced mass as compared to that of H/D exchange.

***Influence of a magnetic field****:* Since different spin states with different magnetic properties are involved in ST phenomena, one should expect that the SCO behavior responds to an applied magnetic field and indicates a change of the ST curve *γ*_HS_*(T)*. An estimate using a simple formula [192] shows that the effect on the ST temperature *T*_1/2_ is expected to be very small. An experiment with a sample of [Fe(phen)_2_(NCS)_2_] placed in a magnetic field of 5 Tesla showed indeed a small effect; *T*_1/2_ was shifted by −0.11 K [192]. Later, similar experiments were carried out with samples placed in high magnetic fields [89–90,193]. The results were in agreement with those of the earlier study [192], though correspondingly larger because of the six-times higher magnetic field used in the latter case.

***Influence of sample preparation****:* It was found that the way of treating an SCO compound may influence strongly the SCO behavior. For instance, ball milling or crushing crystals in a mortar often increases the residual HS fraction in the low-temperature region and tends to make the ST curve more gradual [194–195]. The explanation is still rather speculative. It has been proposed that these effects are mainly caused by crystal defects introduced by mechanical treatment (ball milling, crushing in mortar) or by rapid precipitation. The particle size has also been reported to play a role [196]. In any case, the sample preparation for physical characterization of a SCO compound is very crucial and should be done with great care.

#### Dinuclear systems

The possibility of combining two properties such as magnetic coupling and SCO in the same molecule was the original motivation for undertaking the study of such Fe(II) dinuclear molecules [197], as well as the possibility of investigating new cooperative behavior through covalent bonding of active sites compared to mononuclear complexes dealt with in the previous section.

**Figure 16:**
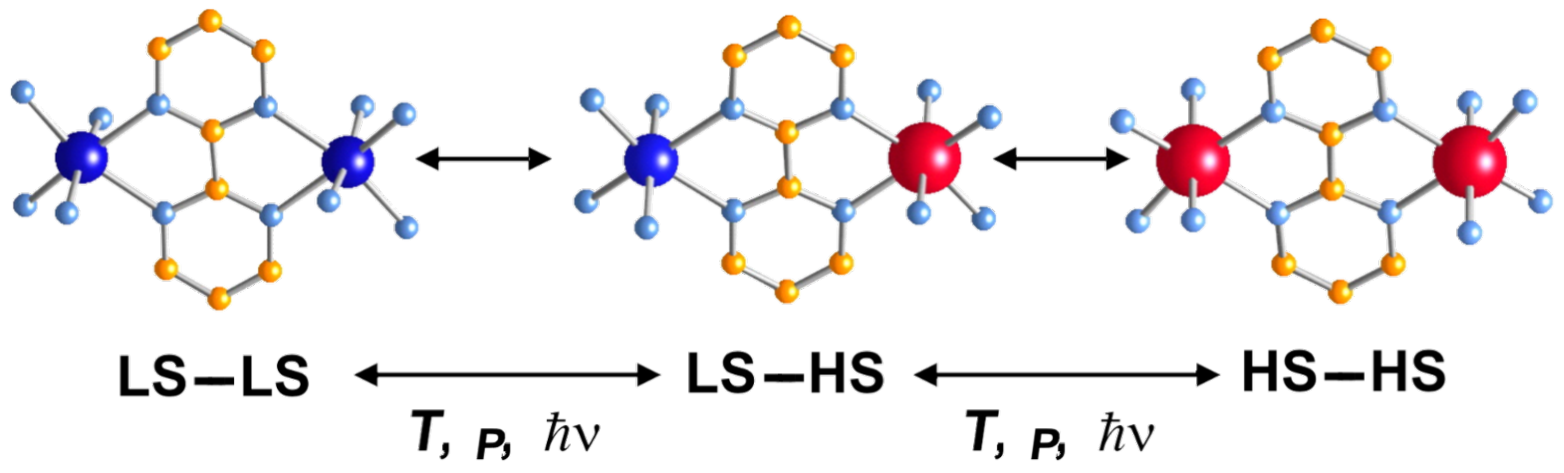
Sketch of the two-step spin transition; [LS–LS] pair is diamagnetic, [LS–HS] is paramagnetic and the [HS–HS] pair is antiferromagnetically coupled.

Dinuclear SCO molecules can adopt three different spin-pair states: A fully diamagnetic state, [LS–LS], with both iron(II) atoms in the LS state; a paramagnetic mixed spin-pair state [LS–HS]; and an antiferromagnetically coupled [HS–HS] state. Stabilization of the [LS–HS] state depends on a subtle balance between intra- and intermolecular interactions in the solid state [198]. Consequently, the thermal dependence of the physical and structural properties can present one-step or two-step spin transitions. The former case involves the [LS–LS]↔[HS–HS] transformation, while in the latter case the intermediate stage responsible for the plateau, at 50% conversion between the two steps, is observed. This may be due to the formation of a 50% mixture of [HS–HS] and [LS–LS] or to the existence of 100% [LS–HS] species. In some cases, switching between the three spin-pair states has been observed upon the action of temperature, pressure or light, which implies competition between magnetic coupling and SCO phenomena (Figure 16).

**Figure 17:**
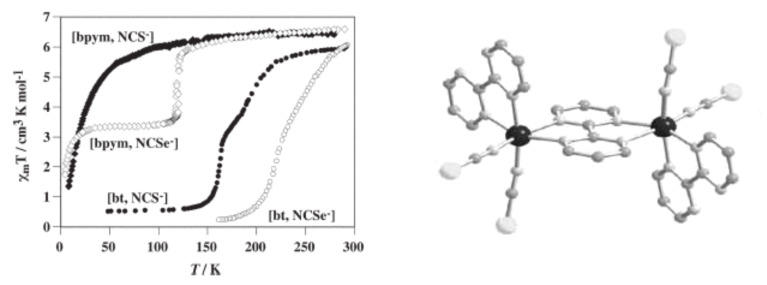
(left) Temperature dependence of χ_M_*T* for {[Fe(L)(NCX)_2_]_2_bpym}(L = bpym or bt and X = S or Se). (right) Crystal structure of {[Fe(bpym)(NCS)_2_]_2_bpym}.

{[Fe(bpym)(NCS)_2_]_2_bpym}(bpym = 2,2’-bipyrimidine) [199] was the first example of a series of compounds designed to this end. Its magnetic behavior is that of two iron(II) atoms in the HS state displaying intramolecular antiferromagnetic (AF) coupling (*J* = −4.1 cm^−1^). In the search for new dinuclear SCO complexes the ligand field strength was fine-tuned by varying the peripheral ligands. Thus, the formal substitution of the peripheral bpym ligands with organic ligands such as bromazepam (bzp) [199], 2,2’-bithiazoline (bt) [200–201], dipyridylamine (dpa) [202] and 6-methyl-2,2-bipyridine (Mebipy) [203] and phen [204] afforded the first evidence of spin crossover. The ligand field strength could also be tuned by changing the coordinated pseudohalide, namely NCS^−^ and NCSe^−^. The crystal structures of the centrosymmetric {[Fe(bpym)(NCS)_2_]_2_bpym}, {[Fe(bt)(NCS)_2_]_2_bpym} and {[Fe(Mebipy)(NCS)_2_]_2_bpym} derivatives are known [197].

bzp and dpa derivatives undergo smooth spin conversions, being rather incomplete in the case of the bzp derivative, while the Mebipy derivative is HS. The most interesting observations have been made with the compounds {[Fe(L)(NCX)_2_]_2_bpym}, where L = bpym or bt and X = S or Se, which have been extensively investigated. The magnetic behavior of these four compounds is depicted in Figure 17. The derivative with bpym and NCS^−^ as peripheral ligands (denoted [bpym, NCS^−^]) seems to fulfill the necessary conditions to observe SCO, however, as mentioned above, this derivative remains in the [HS–HS] state and displays intramolecular AF coupling but not SCO. The substitution of the NCS^−^ with an NCSe^−^ group increases the ligand field strength around the iron atoms and the resulting dinuclear species [bpym, NCSe^−^] undergo a sharp ST around 120 K, which involves 50% of the iron(II) atoms. The [bt, NCS^−^] compound, formally derived from the replacement of bpym with the stronger ligand bt, displays two spin transitions separated by a plateau at 50% conversion and centered at 175 K. The first and second steps take place at ca. *T*_1/2_^1^ = 163 K and *T*_1/2_^2^ = 197 K, respectively. Finally, when the NCS^−^ group is replaced with the NCSe^−^ group in the latter compound, the ligand field increases and the resulting [bt, NCSe^−^] derivative shows a similar two-step ST at higher temperatures, *T*_1/2_^1^ = 223 K and *T*_1/2_^2^ = 265 K.

**Figure 18:**
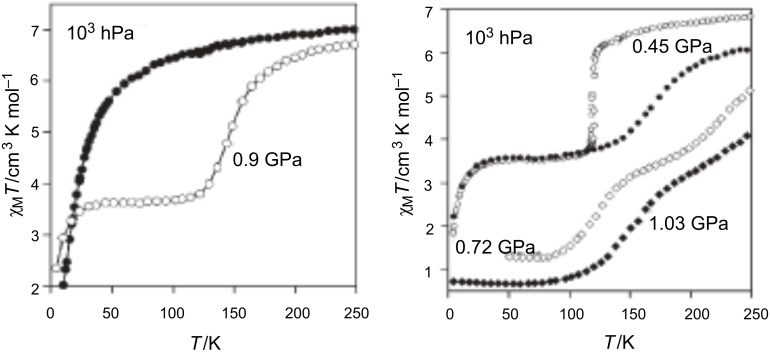
Temperature dependence of χ_M_*T* for [bpym, NCS^−^] (left) and [bpym, NCSe^−^] (right) at different pressures (Reproduced with permission from [206]. Copyright 2001 American Chemical Society).

Because the SCO ion is larger in the HS state than in the LS state, an alternative way to induce SCO is to pressurize the sample at constant or variable temperature. Applying external pressure is also an efficient way to tune the ligand field strength in HS complexes that are not far from the crossing point of the HS and LS states. Figure 18 displays the thermal variation of the magnetic susceptibility, recorded at constant hydrostatic pressure at different increasing/decreasing pressures, for the dinuclear systems [bpym, NCS^−^] and [bpym, NCSe^−^]. Between ambient pressure (10^3^ hPa) and ca. 0.4 GPa the compound [bpym, NCS^−^] does not undergo SCO [205]. However, for pressures higher than 0.6 GPa this compound shows the onset of a very incomplete SCO, which coexists with the magnetic exchange. At 0.9 GPa the compound undergoes a ST involving 50% of the iron(II) atoms with *T*_1/2_ = 150 K. A further increase of pressure up to 1.1 GPa does not alter the spin transition; it is similar to that observed for [bpym, NCSe^−^] at ambient pressure [206]. In addition, the latter dinuclear species experiences a two-step ST for pressures higher than 0.45 GPa (Figure 18 right). This behavior resembles that observed for [bt, NCS^−^] and [bt, NCSe^−^] at ambient pressure.

**Figure 19:**
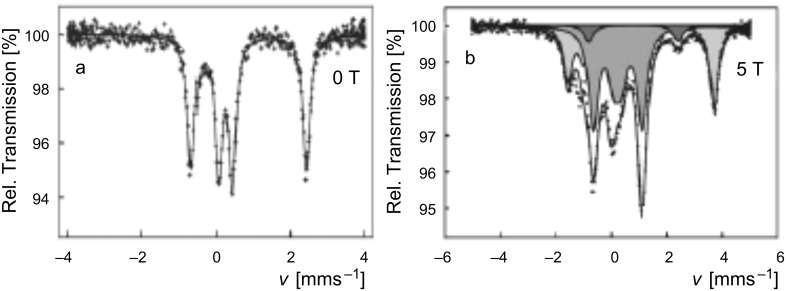
^57^Fe Mössbauer spectra of [bpym, NCSe^−^] measured at 4.2 K at zero field (a) and at 5 T (b) (see text) [207–208] .

The existence of [HS–HS], [LS–HS] and [LS–LS] spin-pair states and the nature of plateaus in ST curves was the subject of conjecture for a long time. However, their unambiguous identification was achieved by using Mössbauer spectroscopy. Standard zero-field Mössbauer spectroscopy reflects the total amount of LS and HS species but cannot distinguish between the spin pairs involved. As an example, Figure 19a shows the zero-field Mössbauer spectrum of [bpym, NCSe^−^] measured at 4.2 K. From the area fractions of the quadrupole doublet of HS-Fe(II) (outer two lines) and that of the LS-Fe(II) (inner two lines) one arrives at an estimate of 50% each in the HS and LS state. No distinction can be made between the presence of a mixture of [HS–HS] and [LS–LS] spin-pair states on the one hand, and 100% of [LS–HS] on the other hand. However, it was realized that at sufficiently low temperature, Mössbauer spectroscopy could identify them by placing the sample in an intense magnetic field [207–208]. The Mössbauer spectrum of [bpym, NCSe^−^] measured at 4.2 K in a magnetic field of 5 T (50 kOe) is shown in Figure 19b. The analysis of the spectrum [207] yields the presence of three components. One corresponds to LS-Fe(II) (52%, grey component) assignable to [LS–LS] and [LS–HS] pairs. The second component [dark grey] is minor, ca. 4%, and arises from [HS–HS] pairs. The third component (light grey), 44%, corresponds to the magnetically split Mössbauer sextet spectrum of an uncoupled iron(II) atom in the HS state. Clearly, the two major components represent the uncoupled paramagnetic [LS–HS] spin pair state (88%) while the remainder corresponds to 8% and 4% of [LS–LS] and [HS–HS] spin pairs, respectively.

Observation of the LIESST effect in dinuclear compounds has been limited for a long time to the [bpym, NCSe^−^] [205,209–210], [bt, NCS^−^] [201,209] and [bt, NCSe^−^] [211] derivatives. These experiments showed, for the first time, the interplay between SCO and antiferromagnetic coupling in the same molecule. The combination of magnetic-field Mössbauer with the LIESST effect has given deeper insight into the dynamics of conversion between the different spin-pair states and at the same time has given support to the hypothesis of the occurrence of three spin-pair states and their transformation by means of temperature, pressure or light [212–213].

Once the existence of the [LS–LS], [LS–HS] and [HS–HS] spin pairs had been proven, the next important step in this research concerned the possibility of photo-switching between them selectively. Initial evidence arose from photomagnetic experiments performed on the [bt, NCS^−^] compound by using different wavelengths [214]. Raman studies have confirmed that the nature of the plateau in the two-step transition of [bt, NCS^−^] is not a 1:1 mixture of [LS–LS] and [HS–HS] species but corresponds to the mixed-spin species [214]. Furthermore, the [LS–LS] state can be selectively switched at low temperatures to the [HS–HS] state and to the [LS–HS] state by using red light (647.1 nm) or infra-red light (1342 nm) [212–213].

The series of new dinuclear iron(II) SCO compounds reported recently by the groups of Real, Gütlich, Murray, Brooker, Kaizaki, Matouzenko, and Garcia, among others, have added new interesting results to this topic [215–235].

**Figure 20:**
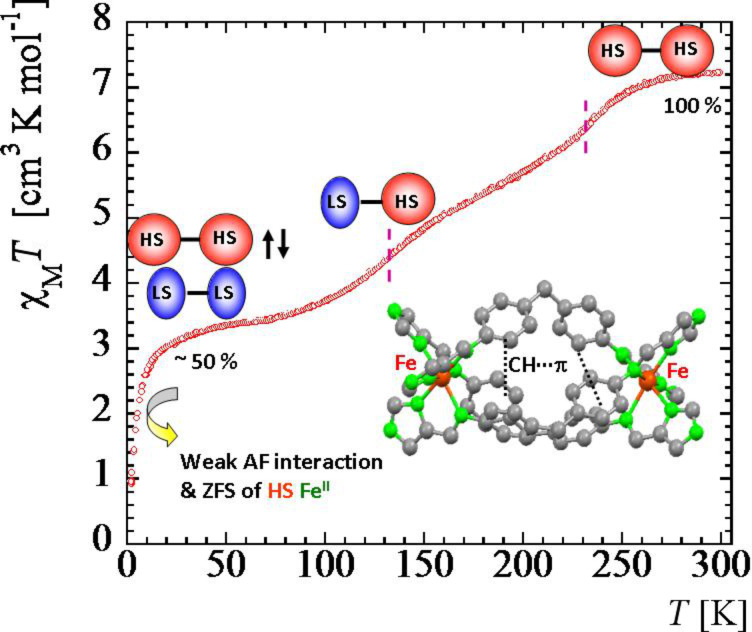
Temperature dependence of χ_M_*T* for [Fe_2_(L)_3_](ClO_4_)_4_**·**2H_2_O showing a complete two-step spin conversion from 100% [HS–HS] pairs to 50/50% [HS–HS] and [LS–LS] pairs as discussed in the text. The triple helicate dinuclear unit with intramolecular CH···π interactions is shown in insert [230].

**Figure 21:**
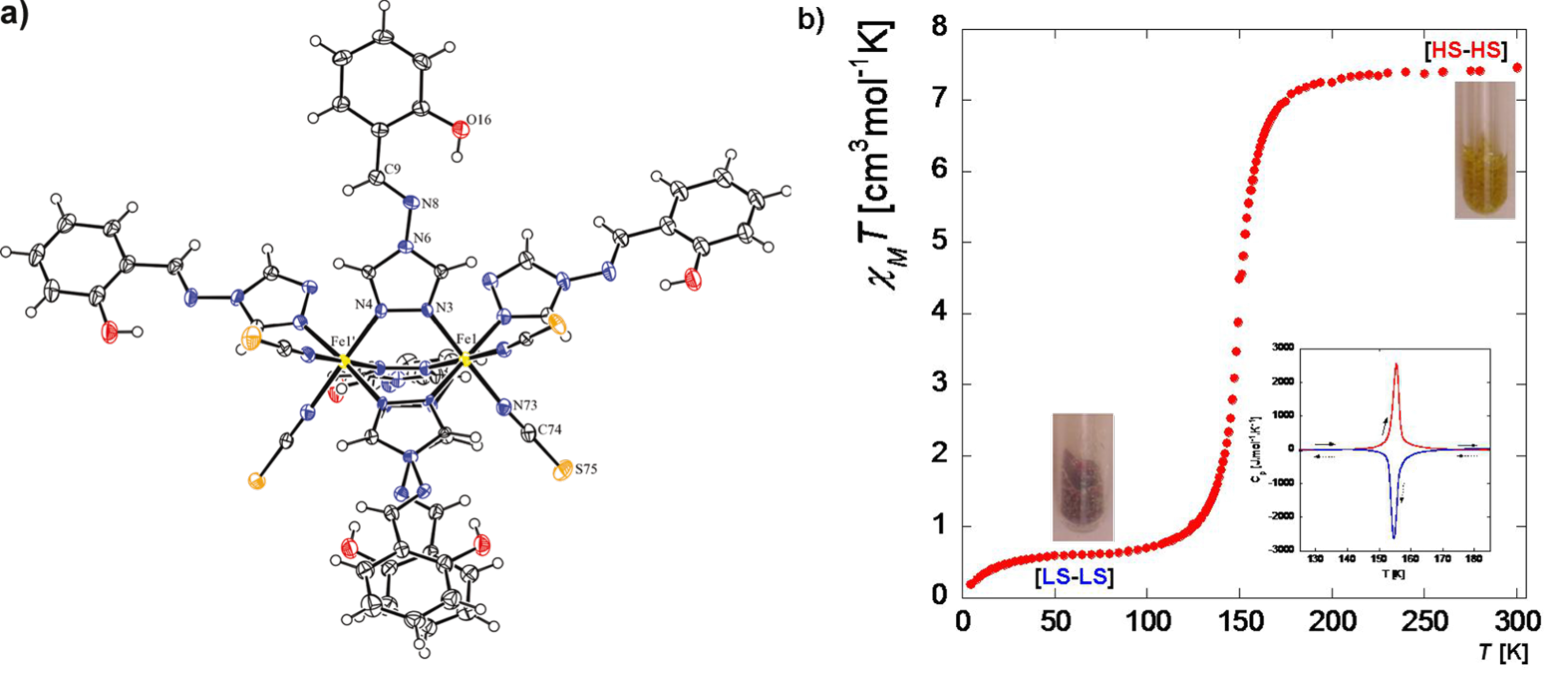
(a) View of the dinuclear unit in the crystal structure of [Fe_2_(Hsaltrz)_5_(NCS)_4_]·4MeOH. (b) Temperature dependence of *χ*_M_*T* versus *T* revealing a steep spin transition from [HS–HS] to [LS–LS] pairs. The tubes show the color change from yellow at room temperature to burgundy at liquid nitrogen temperature. The DSC response, shown in the inset, confirms that a first-order phase transition takes place on cooling and warming [235].

The triple helicate dinuclear iron(II) complex, [Fe_2_(L)_3_](ClO_4_)_4_**·**2H_2_O [217], presents an unusual two-step spin conversion on cooling, proceeding very smoothly at *T*_1/2_^(1)^ ~ 240 K and *T*_1/2_^(2)^ ~ 120 K (Figure 20). This thermochromic material does not switch its spin state on cooling further below 20 K, as demonstrated by zero-field Mössbauer spectroscopy despite the steep decrease of χ_M_*T*, which is due to both weak AF coupling and zero-field splitting of HS Fe(II) ions [230]. As revealed by applied-field ^57^Fe Mössbauer spectroscopy carried out at 4.2 K, the thermal spin conversion for one half of the Fe(II) active sites is complete. The existence of a plateau tracked in the spin conversion curve at ~200 K and identified through the variation of hyperfine parameters of an uncoupled iron(II) site indicates the presence of an [LS–HS] intermediate spin state [230]. Interestingly, spin pairs can also be revealed without the need for an external magnetic field [221]. Very recently, the first crystal structures in the HS and LS states of a dinuclear iron(II) complex with three *N*1,*N*2-1,2,4-triazole bridges, which is considered as a structural model of 1,2,4-triazole iron(II) 1D chains, was reported. The abrupt change in the magnetic properties of [Fe_2_(Hsaltrz)_5_(NCS)_4_]·4MeOH (Hsaltrz = *N*-salicylidene-4-amino-1,2,4-triazole), at *T_1/2_* ~ 150(1) K, from [HS–HS] to [LS–LS] pairs results from the cooperative manifestation of the ST process thanks to intra- and intermolecular interactions mediated by 1,2,4-triazole and supramolecular interactions, respectively (Figure 21) [235]. A similar cooperative situation was found within the pentanuclear assembly [Fe_2_(totrz)_5_(NCS)_4_]_2_[Fe(totrz)_2_(NCS)_2_(H_2_O)_2_]·*n*H_2_O with totrz = 4-(*p*-tolyl)-1,2,4-triazole, which also includes similar binuclear iron(II) entities that are linked to a non-SCO mononuclear unit by hydrogen bonding [236]. As a result, a very sharp ST is observed. However, the cooperativity is not sufficient to induce a hysteretic effect.

Such investigations have pointed out two different SCO processes occurring in dinuclear iron(II) units, namely stepwise [LS–LS]↔[LS–HS]↔[HS–HS] and direct [LS–LS]↔[HS–HS] transformations. Moreover, it has been evidenced that the plateau observed between two separate spin transitions can be associated with the existence of the mixed spin state [LS–HS], or with a 1:1 mixture of the [HS–HS] and [LS–LS] states [197,213]. In addition, the mixed spin state [LS–HS] has been structurally characterized for dinuclear entities of different chemical coordination sphere. Considerable progress has been made in understanding both SCO processes, although the factors that determine the preference of each SCO mechanism have not yet been elucidated.

#### Systems with higher nuclearity and cages

**Figure 22:**
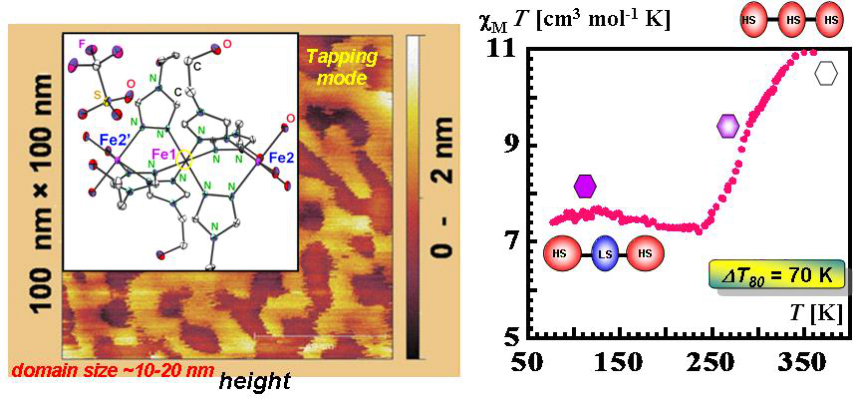
(left) AFM pattern recorded in tapping mode at room temperature on hexagonal single crystals of [Fe_3_(hyetrz)_6_(H_2_O)_6_](CF_3_SO_3_)_6_. A domain size of ~10–20 nm was identified [239]. The insert shows the crystal structure of the trinuclear unit as well as the noncoordinated anion [98]. (right) *χ_M_T* versus *T* showing a gradual spin conversion from [HS–LS–HS] trimers below ~250 K to [HS–HS–HS] trimers above ~350 K. The color of the crystals is shown as well as Δ*T*_80_, which represents the temperature interval for which 80% of ions have switched [98].

Ranging between dinuclear units and coordination polymers, a few systems of higher nuclearity have been reported. The series of iron(II) linear trinuclear complexes of formula [Fe_3_(4-R-1,2,4-triazole)_12−y_(H_2_O)_y_](anion)_6_·*n*H_2_O have revealed gradual spin conversions, as expected with the switch of a single iron atom isolated from its two counterparts [98,237–246]. Indeed, their crystal structures reveal a central FeN_6_ SCO site, which is bridged by three 1,2,4-triazole ligands to peripheral HS iron sites, which are coordinated by water molecules, as shown, for instance, for [Fe_3_(hyetrz)_6_(H_2_O)_6_](CF_3_SO_3_)_6_ (hyetrz = 4-(2’-hydroxyethyl)-1,2,4-triazole) (Figure 22) [98]. Interestingly, this complex presents a 50% LS/HS spin population at room temperature (*T*_1/2_ = 290 K), with pale mauve, thermochromic, hexagonal crystals. This material was suggested for the purpose of imaging spin domains at the nanoscale using a coating layer by AFM [239].

More recently, the first triangular SCO trinuclear complex, [Fe_3_L_2_(NCS)_4_(H_2_O)] with L = 1,3-bis[(2-pyridylmethyl)imino]propan-2-ol, was reported [247]. The magnetic behavior is similar to the 1,2,4-triazoles trinuclear complexes discussed above with two external HS iron(II) ions and one central SCO center, but with a different donor set (N_4_O_2_) [247]. While an iron(II) trinuclear complex with three SCO sites is still awaited, several tetranuclear iron(II) complexes have been communicated.

**Figure 23:**
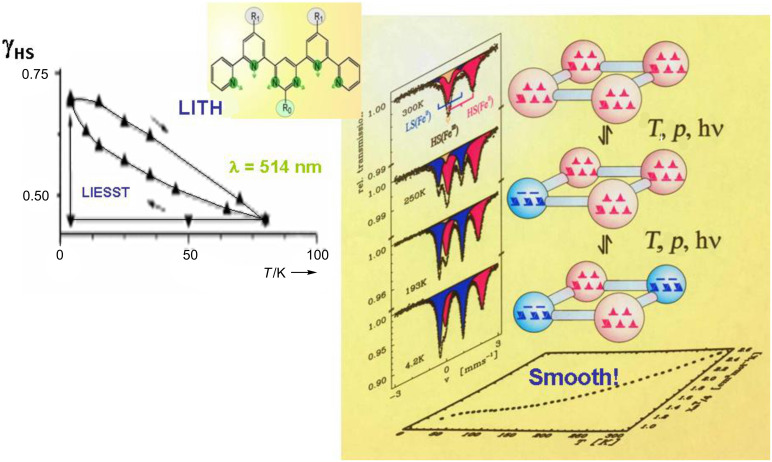
(right) Stepwise SCO in an Fe_4_ [2 × 2] grid, which reveals a smooth magnetic profile under ambient and applied pressure. ^57^Fe Mössbauer spectra are also shown. (left) This material is also switchable by light (LIESST). A LITH loop is observed during continuous irradiation with green light. The ligand system used for the self assembly process is also depicted [249].

Compartmental polypyridyl ligands are considered as key components to induce the formation of grid-type complexes by self assembly [248]. The first iron(II) tetranuclear complexes, [Fe_4_L_4_]A_4_ (A = BF_4_^−^, ClO_4_^−^, PF_6_^−^) with pyrimidine-derived compartmental ligand strands were described by Lehn et al. [249–251]. [Fe_4_L_4_](BF_4_)_4_ exhibits a continuous and incomplete SCO between three magnetic levels [249]. Interestingly, intramolecular cooperative effects, which were expected from the tetranuclear arrangement of spin carriers, are not evident from temperature-dependent magnetic measurements, which revealed a very smooth spin conversion (Figure 23, right). However, Mössbauer measurements under permanent green-light irradiation revealed hidden cooperative effects thanks to the detection of a light-induced thermal hysteresis (LITH) loop (Figure 23, left).

Supramolecular [2 × 2] grid-type complexes constructed from the same ligand system and arranged either in a 1D columnar superstructure or in a wall-like 2D layer, also exhibit a gradual spin conversion [252], which was also found for another grid based on a pyrazolate binucleating bpy ligand [248].

Cyanide bridged ferrous tetranuclear squares exhibiting smooth spin conversion have attracted recent interest. The spin state switching of [Fe_4_(μ-CN)_4_(L)_4_(tpa)_2_](PF_6_)_4_ (tpa = tris(2-pyridylmethyl)amine) occurs in two steps at ~160 K and ~380 K for the L = bpy derivative [253–254] and around room temperature for the L = phen complex [255]. Above room temperature, spin state conversion is reached for the tetranuclear [Fe_4_(μ-CN)_4_(bpy)_4_(bpym)_4_](PF_6_)_4_·6CH_3_OH·4H_2_O [256]. Other tetranuclear square iron(II) complexes constructed on tpa and dca [257] or with oxo bridged ligands were also reported [258–259]. DFT studies of these systems, which all display gradual spin state conversion, have just started [260–261].

**Figure 24:**
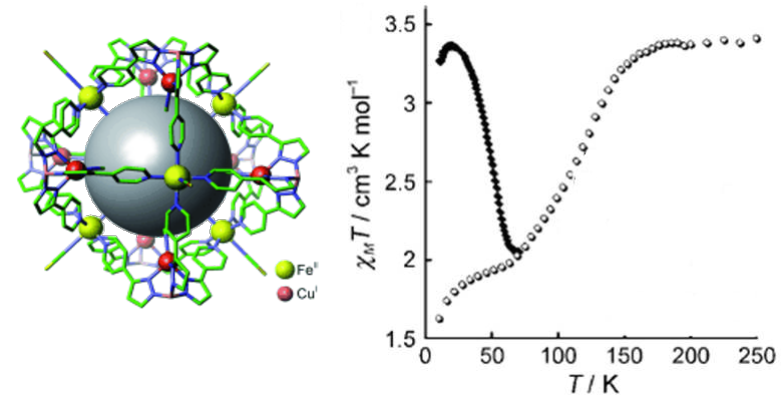
(left) View of the discrete nanoball made of Fe(II) SCO units as well as Cu(I) building blocks. (right) *χ_M_T* versus *T* for the nanoball cluster showing both a thermally induced spin state conversion with *T*_1/2_ ~125 K as well a thermal HS to LS relaxation after LIESST [263]. (Reproduced with permission from [263]. Copyright 2009 Wiley-VCH Verlag GmbH & Co. KGaA).

SCO complexes with higher nuclearity have not yet been reported except for a heptanuclear mixed valence iron(III) complex [262] and a discrete porous iron(II) nanoball made on Cu(I) building blocks presenting a gradual spin state conversion, which can also be switched by light irradiation at liquid He temperatures (Figure 24) [263–264].

#### Polymeric 1D, 2D and 3D systems

The number of polymeric iron(II) SCO compounds has rapidly increased in the context of the booming interest on coordination polymers (CPs) [265] and metal organic frameworks (MOFs) [266]. Most of these materials incorporate multidentate *N*-donor heterocyclic bridging ligands, such as 1,2,4- or 1,2,3-triazole, 1-R or 2-R-tetrazole, and polypyridine-like derivatives, as well as tetra- and dicyanometallate, polynitrile anions, and tetradentate Schiff bases [13,267–271]. These coordination complexes usually exhibit thermally induced spin conversions of a gradual nature. In certain cases, abrupt spin transitions with hysteresis effects, whose width is strongly dependent on the type of molecular bridge between iron(II) sites as well as on crystal packing considerations, are observed. A few examples of iron(III) and cobalt(II) SCO CPs have also been described [272–275]. In this section, we review prototype materials as well as highlight recent examples of CPs and MOFs constructed from ligands based on organic molecules.

**Figure 25:**
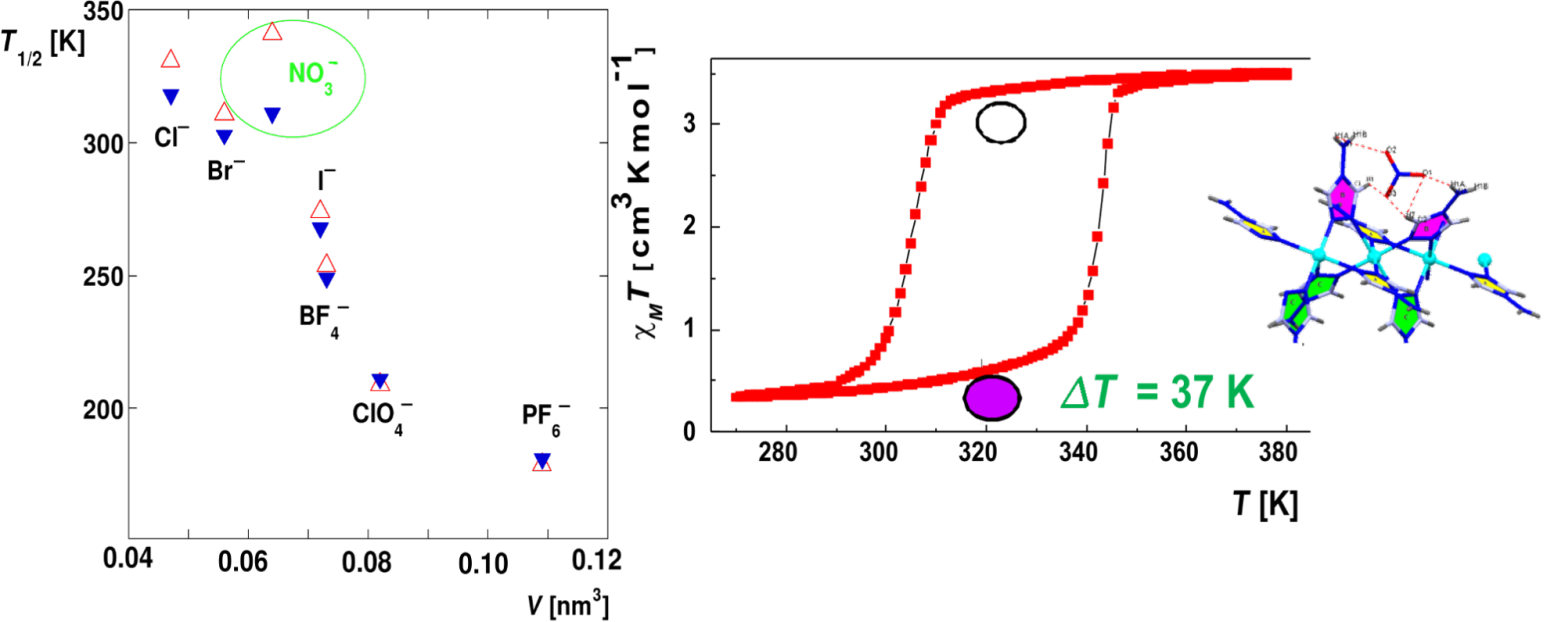
(left) Linear dependency between *T*_1/2_ in the heating (Δ) and cooling (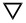) modes versus the anion volume for the series [Fe(NH_2_trz)_3_]A_2_. The circle highlights the case of the nitrate derivative that displays a larger hysteresis width. (right) *χ_M_T* versus *T* of [Fe(NH_2_trz)_3_](NO_3_)_2_ showing a hysteretic ST slightly above the room-temperature region. The nitrate anion is hydrogen-bonded to NH_2_trz (in pink) coordinated to two metal centers, thus acting as a supramolecular link in addition to the bridge made by triple *N*1,*N*2-1,2,4-triazoles [293].

**1D chain compounds**: 4-R-1,2,4-triazole based iron(II) chain compounds have been widely studied, due to their tremendous potential for practical applications based on their thermochromic effect (memory devices, displays, sensors) [276–280], and are currently the topic of intense miniaturization efforts [281–287]. [Fe(4-R-1,2,4-triazole)_3_]A_2_·Solv are composed of linear chains in which the iron(II) ions are connected by three *N*1,*N*2-1,2,4-triazole ligands (Figure 11a). Noncoordinated species, such as counteranions (A) and solvent molecules (very often water) are confined between the chains. The coordination linker in these polymeric compounds is rigid enough to allow an efficient transmission of elastic cooperative effects leading to a hysteresis loop of width ranging from ~2–20 K [268]. Inspired by the systematic parallel shift of ST curves under hydrostatic pressure that is discussed above [177–178,180], several synthetic approaches have been successfully developed to reach the room temperature region by making use of internal pressure provided by anion (Figure 25), solvent and ligand substitution in the crystal lattice [159,268,288]. In a few cases, the hysteresis width could be increased up to ~35–50 K, when noncoordinated anions were connected to 1D chains by hydrogen bonds [139,180,289–293]. Consequently, abrupt spin transitions with broad thermal hysteresis loops along with a color change from pink/purple (LS) to white (HS) have been observed (Figure 25, right) [276–280]. Remarkably, a few examples of this family of materials can also be switched by light [181,294–297].

**Figure 26:**
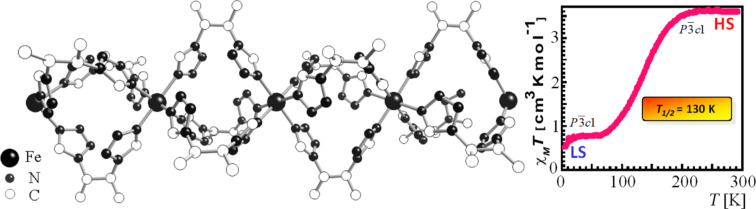
(left) View of the linear chain structure of [Fe(1,2-bis(tetrazol-1-yl)propane)_3_]^2+^ along the *a* axis. (right) *χ_M_T* versus *T* showing a gradual spin conversion for the perchlorate derivative, with *T*_1/2_ = 130 K [298].

[Fe(1,2-bis(tetrazol-1-yl)propane)_3_](ClO_4_)_2_ represents the first SCO chain whose crystal structure has been determined by X-ray diffraction, both in the HS and LS states (Figure 26). Despite its polymeric nature, the spin conversion is very gradual. The virtual absence of cooperativity, also noted in the isostructural tetrafluoroborate derivative, has been attributed to the flexibility of the bridging network, as well as the absence of intermolecular contacts between 1D chains that cannot transmit efficiently the structural changes associated with the spin change in the crystal lattice. This compound exhibits, in addition, the LIESST effect, which was observed for this substance class for the first time [298]. A similar gradual SCO behavior was revealed for other 1D iron(II) chains with triply [299–300] and doubly bridging bis-tetrazole ligands [301]. The situation differs for [Fe(μ-btzmp)_2_(btzmp)_2_](ClO_4_)_2_ (btzmp = 1,2-bis(tetrazol-1-yl)-2-methylpropane), which displays a steep and hysteretic spin transition, partly attributed to an anion order/disorder phenomenon [302]. Other 1D iron(II) chains made of two bridges that cushion elastic interactions [303] were also reported [304–307].

Noticeably, numerous examples of 1D chains made of flexible single bridge spacers systematically reveal a gradual spin conversion as noted for 1,2-bis(4-pyridyl)etha(e)ne [308], 4,4’-bpy [309–310], substituted triazine/pyrimidine [307,311–313], pyrazine [314], dca [315] and tcpd anions [316]. Some other very interesting 1D chains were recently prepared by Weber et al. using tetradentate Schiff bases allowing them to set up a N_4_O_2_ core and to observe a variety of magnetic behavior including gradual, hysteretic, complete, incomplete and even double-step spin conversions [317–324]. We anticipate that further 1D SCO chains will be reported with both rigid linkers as well as with predesigned modules suitable for supramolecular contacts to enhance communication between spin centers throughout the crystal lattice.

**2D and 3D networks**: A crystal-engineering strategy towards obtaining polymeric systems of higher dimensionality makes use of multidentate organic ligands (that are suitable for SCO) connected by different spacers, which boosts the possibilities to increase the dimensionality. The first ones can potentially coordinate several metals whereas the second ones can increase the degrees of liberty/flexibility of the coordinating groups in order to promote coordination in several directions [27].

**Figure 27:**
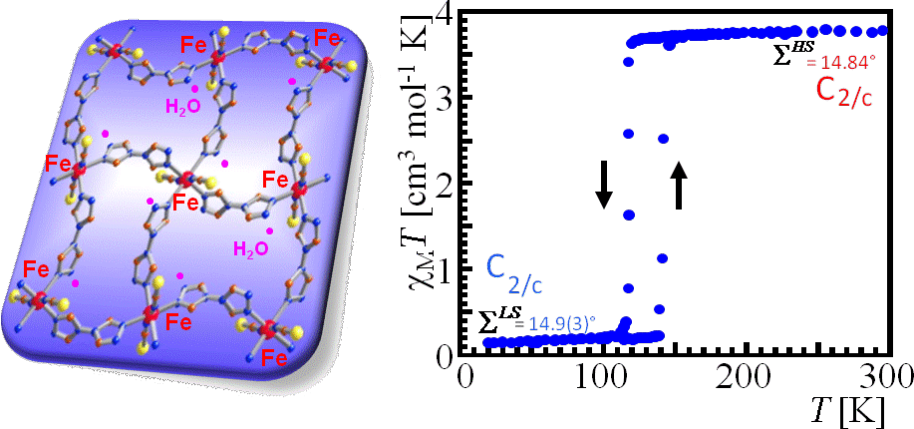
(left) View of the 2D layered structure of [Fe(btr)_2_(NCS)_2_]·H_2_O (at 293 K). The water molecules (in violet) are located between the layers and H-bonded to triazole ligands [325]. (right) *χ*_M_*T* versus *T* showing a square-shaped hysteresis loop on cooling. The structural distortion parameter Σ remains the same in the HS and LS states revealing that the local symmetry is not modified by the spin state change [330].

[Fe(btr)_2_(NCX)_2_]·H_2_O (X = S, Se) represent the first 2D ST compounds [325–326]. The sulfur derivative displays on cooling a square-shape hysteresis loop of width 21 K, associated with an extremely abrupt and completely thermally induced ST, free of any structural phase transition (Figure 27). Its crystal structure consists of iron(II) ions linked by btr in two orthogonal directions establishing a grid. The isothiocyanate anions, coordinated in the *trans* position, prevent the formation of a 3D lattice. The layers are connected by means of van der Waals forces and weak hydrogen bond bridges involving the water molecules, which are also H-bonded to peripheral nitrogen atoms of the triazole. In fact, the water molecule is crucial to maintain spin state switching [325]. The Se derivative displays, as expected with the increase of ligand field strength, a ST at higher temperature but with a narrower hysteresis (~6 K) [326]. A similar 2D ST grid was found for [Fe(baztrz)(pyz)(NCS)_2_]·4H_2_O (baztrz = *trans*-4,4′-azo-1,2,4-triazole) with the replacement of one btr by pyrazine [327]. Using only one bridging bis-tetrazole afforded another 2D SCO grid but with a less cooperative behavior [328–329].

**Figure 28:**
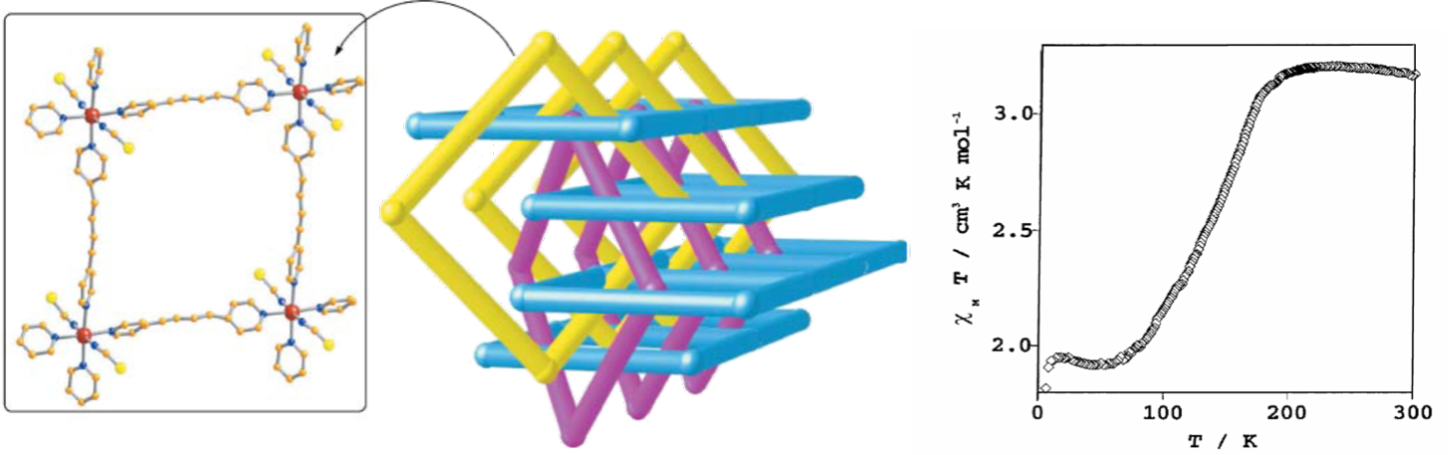
(left) Three interpenetrated square networks for [Fe(bpb)_2_(NCS)_2_]·MeOH. (right) *χ_M_T* versus *T* plot shows a gradual and incomplete spin conversion [334].

Several complexes of formula [FeL_2_(NCS)_2_]·Solv show a similar layered, stacked structure with bis-monodentate pyridine-like ligands instead of btr [267–270]. This includes bispyridylethylene (bpe, Solv = MeOH) [331], *trans*-4,4’-azopyridine (azpy, Solv = MeOH, EtOH and PrOH) [332–333], and 1,4-bis(4-pyridylbutadiyne) (bpb, Solv = 0.5 MeOH) [334]. The larger size of bpe and azpy allow interpenetration of two equivalent sets of layers, whereas for the bpb a framework made up of three different arrays of mutually perpendicular, interlocked 2D networks was obtained (Figure 28). A gradual and incomplete SCO behavior is observed due to the flexible link between the iron(II) ions within a single layer, despite the interlocked character of the material.

The first ST grid with a 1-R-tetrazole was discovered for [Fe{N(entz)_3_}_2_](BF_4_)_2_ with a tripodal ligand, N(entz)_3_ = tris[2-(tetrazol-1-yl)ethyl]amine by Rudolf et al. [335–336].

**Figure 29:**
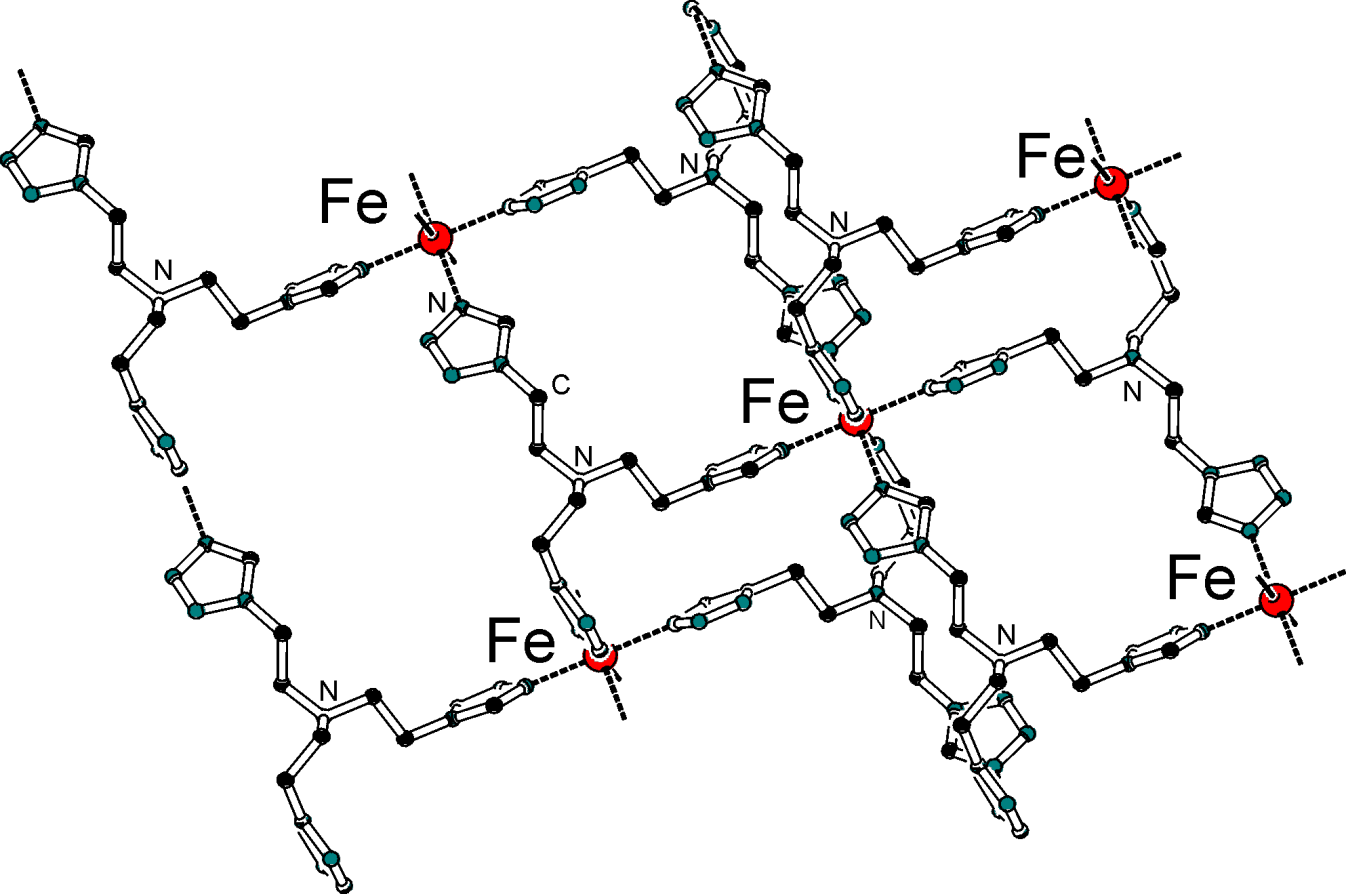
Part of the crystal structure of [Fe{N(entz)_3_}](BF_4_)_2_ (*T* = 293 K) [335–336]. (Reproduced with permission from [23]. Copyright 1994 Wiley-VCH Verlag GmbH & Co. KGaA).

The coordination occurs through the *N*4 nitrogen atoms of the tetrazole rings that are brought by N(entz) (Figure 29) [335–336]. An extremely abrupt ST with thermal hysteresis was observed (*T*_1/2_^↑^ = 176 K and *T*_1/2_^↓^ = 167 K). Recently, a honeycomb-like pattern with cages occupied by disordered anions was discovered for [Fe{C(mtz)_3_}_2_]A_2_, (A = ClO_4_^−^, BF_4_^−^) built from another spider-like ligand, C(mtz)_3_ = tris(tetrazol-1-ylmethyl)methane. These 2D materials both display a sharp ST at *T*_1/2_ = 193 K (BF_4_^−^) and *T_1/2_* = 176 K (ClO_4_^−^) [337]. [Fe(trptrz)_2_](BF_4_)_2_·5H_2_O with trptrz = tris-3-[1,2,4]triazol-4-ylpropyl phloroglucinol, obtained thanks to a Mitsonubu coupling, is another example of 2D SCO CP based on a tripodal ligand. It reveals a gradual spin conversion due to the flexible propyl group attached to the triazole ligand, which cushions elastic interactions [338]. [Fe(bbtr)_3_]A_2_, where A = ClO_4_^−^ [339–340] or BF_4_^−^ [340–341] (bbtr = 1,4-di(1,2,3-triazol-1-yl)butane), are other 2D CPs with a (3,6) network topology presenting a hexagonal sheet structure. While the perchlorate derivative displays an abrupt hysteretic ST around 100 K, the tetrafluoroborate stays in the HS state. Both materials are currently the topic of extensive optical investigations [342–345].

Very recently, highly robust 2D neutral Fe(II) MOFs have been reported for [FeL_2_] with L = 3-(2-pyridyl)-5-(3-pyridyl)-1,2,4-triazole or 3-(3-methyl-2-pyridyl)-5-(3-pyridyl)-1,2,4-triazole. These materials display two-step SCO at remarkably high temperatures (the highest step is above 500 K) [346], which would make them good candidates for CVD deposition of thin films.

**Figure 30:**
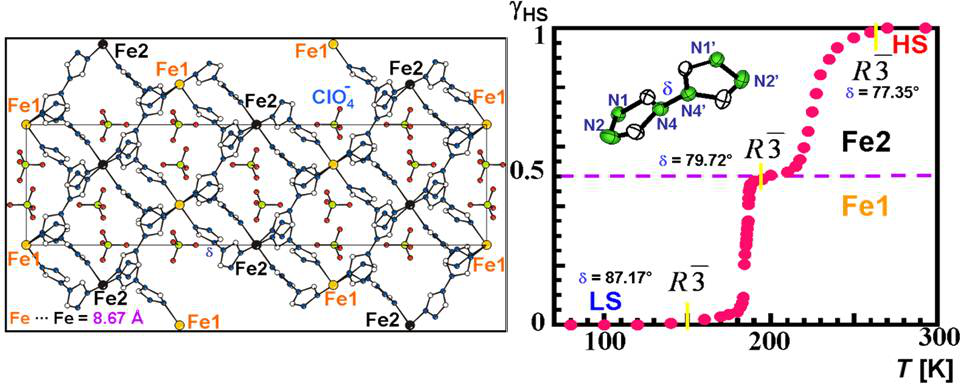
(left) Projection of the crystal structure of [Fe(btr)_3_](ClO_4_)_2_ along the *c* axis revealing a 3D structure with Fe1 and Fe2 sites. The perchlorate anions occupy the voids. (right) HS molar fraction determined by ^57^Fe Mössbauer spectroscopy. Note the variation of the dihedral angle δ between triazole moieties accompanying the spin conversion, which is greater in the lower branch that shows the steeper spin conversion. The space group is also given at three temperatures [347].

[Fe(btr)_3_](ClO_4_)_2_ is the first 3D ST compound [347]. Its crystal structure (Figure 30) is made of iron(II) ions connected by btr ligands at the nitrogen atoms that occupy the 1 and 1’ positions. The noncoordinated perchlorate anions are to be seen in the voids of the 3D architecture. A spin conversion proceeding in two steps was detected by magnetic susceptibility, Mössbauer spectroscopy, and differential scanning calorimetry upon cooling. Temperature-dependent ^57^Fe Mössbauer spectroscopy and a detailed single-crystal X-ray analysis have together demonstrated that the formation of a plateau around ~200 K was due to a consecutive spin conversion occuring at two crystallographically inequivalent iron sites [347]. The narrow hysteresis that can be seen in the lower branch of the spin conversion curve by magnetic susceptibility measurements (not shown in Figure 30), is free of any crystallographic phase transition, and is associated with a higher variation of the dihedral angle δ between the triazole moieties, compared to the higher branch of the SCO curve, which is smoother (Figure 30). A similar 3D network was observed for [Fe(1,3-bis(tetrazol-2-yl)propane)_3_](ClO_4_)_2_·2EtOH that revealed a smooth spin conversion again caused by the flexibility of the spacer between tetrazole molecules [348].

A 3D coordination network constructed from two interlocked cube units was observed for the complexes [Fe(baztrz)_3_]A_2_·2H_2_O (A = ClO_4_^−^, BF_4_^−^), which both display an abrupt transition around room temperature [328]. Such an interlocked architecture was first reported for the 3D CPs [Fe(btzb)_3_]A_2_·Solv, (A = ClO_4_^−^, PF_6_^−^; Solv = MeOH, H_2_O) (btzb = 1,2-bis(tetrazole-1-yl)butane) [349–351]. An abrupt SCO behavior is observed in contrast to the 3D bis-tetrazolate SCO polymer [Fe_2_(H_0.67_bdt)_3_]·13H_2_O with H_2_bdt = 5,5′-(1,4-phenylene)bis(1*H*-tetrazole) [352–353]. Another bidentate ligand based on a pyridine conjugated Schiff base, afforded a CP with a diamond-like 3D network made of FeN_4_O_2_ sites and displaying a gradual spin conversion [354].

An alternative strategy to bis-organic ligands makes use of cyanometallates [270], and to a minor extent cyanide [267], which afforded a large series of fascinating single or interpenetrated 2D and 3D lattices of various topologies and which are covered in review articles from Real et al. [267–270]. Porous materials based on this category as well as Hofmann-like cyanide compounds and other systems are reviewed below. At this stage, many more examples of bis-triazole iron(II) SCO CPs are anticipated after the communication of a revised one-step synthesis of btr and derivatives thanks to a simplified transamination method reducing the reaction time, increasing the yield and avoiding tedious chromatographic separations [355] and which has already afforded numerous CPs and MOFs.

### SCO in nanomaterials

**Figure 31:**
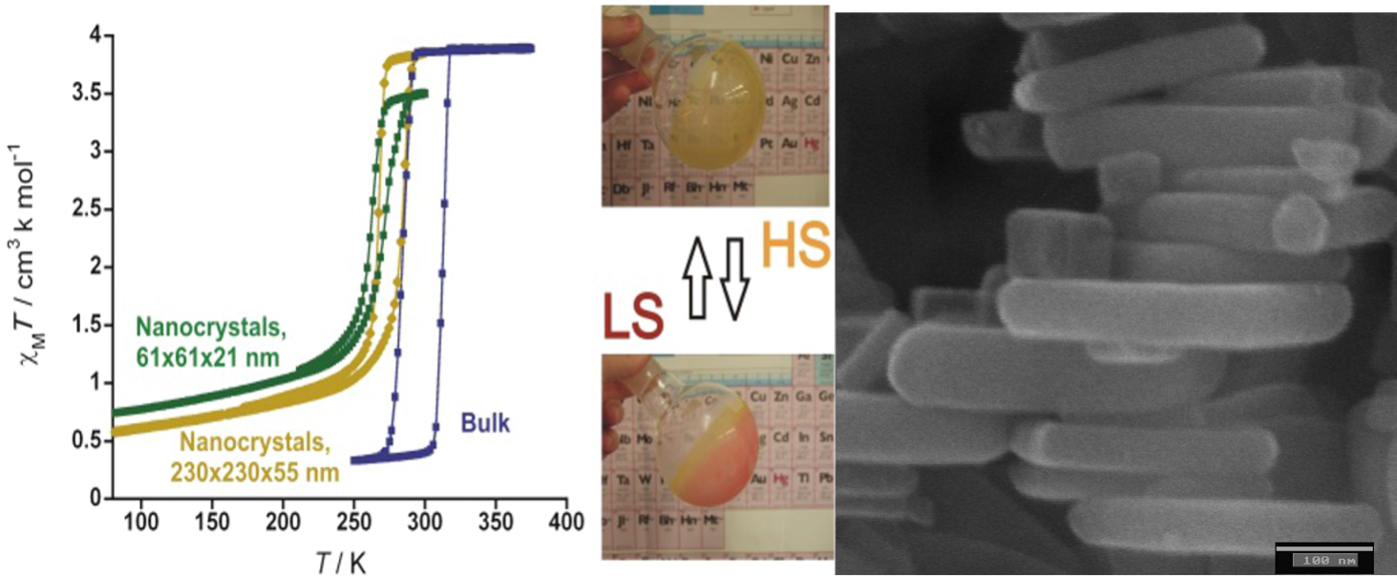
Size-dependent SCO properties in [Fe(pz)Pt(CN)_4_] (left), change of color upon spin state transition (middle) and image of the nanocrystals (right) [359].

A rational control of the growth of SCO materials at the nanometric scale and the study of the size-dependent SCO properties is crucial for their successful integration in functional devices [356–358]. In fact, determination of the critical particle size or film thickness that preserves a complete ST with hysteretic behavior is a vital factor in the nanominiaturization of a SCO material. Recent studies have involved the preparation of nanocrystals [359–360] and thin films [361–362] of the 3D coordination polymer [Fe(pz)Pt(CN)_4_] (pz = pyrazine) [363] exhibiting size-dependent ST characteristics. Bulk microcrystalline samples undergo very cooperative ST with *T*_1/2_^↓^ = 285 K and *T*_1/2_^↑^ = 309 K. The corresponding nanocrystals of average dimensions 200 × 200 × 60 nm and 60 × 60 × 30 nm display practically complete spin transitions, demonstrating a decrease of the critical temperatures and hysteresis widths with decreasing size of the crystallites (Figure 31) [359]. This effect is even more pronounced for 10–20 nm sized nanoparticles, where only around 1/3 of the Fe(II) ions undergo ST characterized by a very narrow thermal hysteresis loop [360]. A similar situation was found for [Fe(pz)Ni(CN)_4_] nanoparticles of about 4 nm coated with Chitosan polymer [364].

The 2D coordination polymers [Fe(3-Fpy)_2_M(CN)_4_] (M(II) = Ni, Pd, Pt), and 3-Fpy = 3-fluoropyridine) [365] have also been the subject of study. Surfactant-free nanocrystals of average dimensions 400 × 400 × 30 nm were synthesized from water-in-oil microemulsions (w/o) and nanoparticles of 200 × 100, 100 × 60, and 70 × 30 nm were prepared by using the coating polymer poly(vinylpyrrolidone) (PVP). The nanocrystals exhibit virtually complete first-order spin transitions centered in the interval 200–225 K, while PVP-coated nanoparticles undergo a continuous second-order ST at much lower temperatures (ca. 160 K) [366].

The dependence of the hysteresis width on particle size in these polymers corresponds well to the behavior predicted by Monte Carlo simulations for cubic or spherical SCO nanoparticles [367]. However, these simulations predict a much weaker (almost negligible) dependence of the transition temperature on particle size than that observed. This discrepancy may reflect the oversimplified nature of the model employed, in which only short-range elastic interactions [368] have been taken into account. Indeed, a significant influence of long-range interactions on the cooperativity of the SCO process was inferred from infrared and Raman studies of the [Fe(pz)M(CN)_4_] and [Fe(pyridine)M(CN)_4_] series [369–370].

A different behavior was observed for surfactant-coated nanoparticles of the triazole-based 1D SCO polymers [Fe(Htrz)_2_(trz)]BF_4_ (Htrz = 1,2,4-4*H*-triazole) and [Fe(4-NH_2_trz)_3_]Br_2_. Twenty-nanometer size nanoparticles of the former display a strong cooperative ST with a large hysteresis loop just like the one observed for the bulk material [281], while in the latter, the abruptness of the ST and the hysteresis width diminish as the particle size decreases with minimal displacement of *T*_1/2_^↑^ [371–373].

The puzzling results reported for 1D triazole based SCO nanoparticles makes difficult any comparison with those results found for 2D and 3D Hofmann Clathrate analogue SCO nanocrystals/nanoparticles.

**Figure 32:**
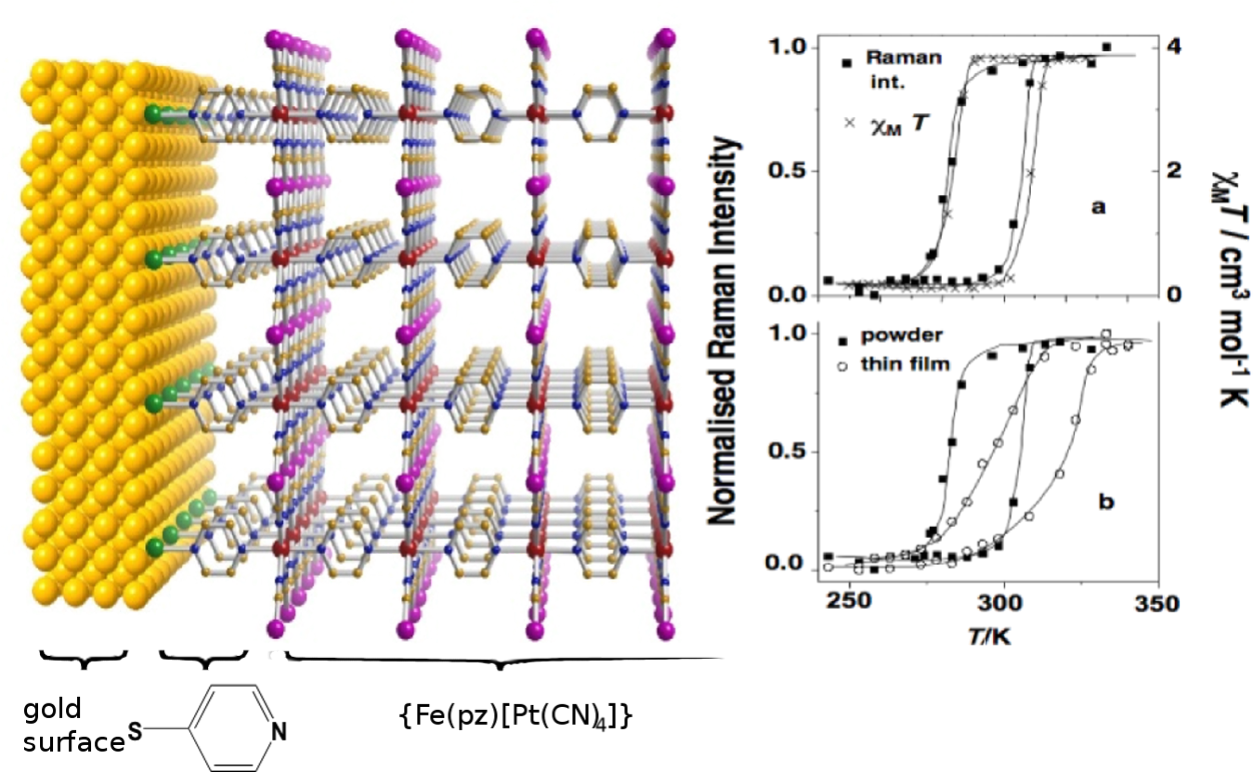
Schematic showing the epitaxial growth of polymer {Fe(pz)[Pt(CN)_4_]} and the spin transition properties of thin films studied by Raman spectroscopy [361].

Thin films of SCO materials were first obtained by using the Langmuir–Blodgett [374] technique or by evaporation of solutions on substrates [375–377]. More recently deposition by sublimation under high vacuum [378–381] or layer-by-layer epitaxial growth techniques were employed (Figure 32) [361–362,382–383]. The ST dependence on film thickness was investigated as well as the LIESST effect in several 3D Hofmann-like polymers and mononuclear compounds.

**Figure 33:**
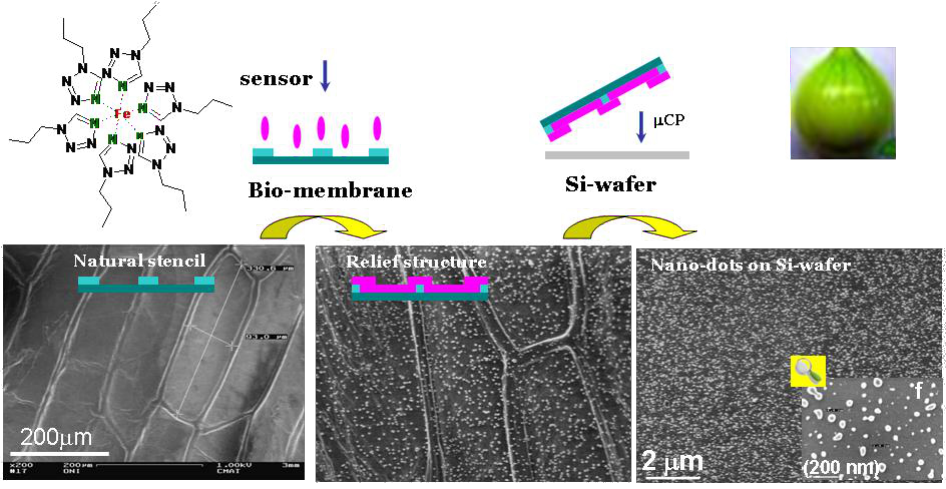
Microcontact printing (μCP) of nanodots on Si-wafer of [Fe(ptz)_6_](BF_4_)_2_ after deposition of crystals on *Allium cepa* membrane [384–385].

A bio-inspired approach was recently introduced to produce [Fe(ptz)_6_](BF_4_)_2_ thin films on a *Alium cepa* membrane [384]. The size of the deposited crystals ranges from nano- to micrometers, and can be controlled depending on nucleation and growth rate, as shown by scanning electron microscopy, which also revealed morphogenesis. The thin films show a thermochromic SCO, which was detected by both UV–vis and ^57^Fe Mössbauer measurements [384–385]. Most interestingly, printed membranes could be used as a natural stencil in soft lithography to produce nanodots of the SCO complex of 30–55 nm size on a Si-wafer and glass supports (Figure 33). This transfer method opens perspectives to study nanoparticles awayfrom their natural confinement and directly on selected supports [384].

### Polyfunctional SCO materials (SCO combined with other functions, e.g., LC, gel and porous properties, electrical conductivity and fluorescence)

In recent years research on solid-state SCO materials has also been directed towards the combination of SCO with other functions, e.g., liquid-crystalline (LC) behaviour, gel and porous properties, electrical conductivity, fluorescence, etc. The idea is to make use of spin state switching in combination with another physical or chemical property that eventually opens pathways to a new generation of multifunctional materials.

#### Spin crossover in Fe(II) metallomesogens

Metallomesogens are metal-containing-liquid crystals [386]. A number of advantages in practical applications may be realized through the combination of SCO and liquid-crystalline behavior, for example, switching and sensing in different temperature regimes, the achievement of thermochromism or photochromism in liquid crystals, external electric- and possibly magnetic-field-based modulation of the SCO properties, and the facile processibility of materials into thin films. In the field of liquid crystals, color change is certainly a phenomenon of interest due to the necessity of color change in a number of applications of liquid crystals, such as laser-addressed devices, passive blocking filters, polarizers based on dichroic effects, or the use of thermochromism.

An initial step aimed at achieving a material combining SCO and LC properties has led to an iron(III) metallomesogen in which both properties are not simultaneously apparent but are seen in different temperature intervals [387]. Subsequently, Co(II) and Fe(II) metallomesogens were synthesized displaying similar properties [388–390]. Recently, the synchronization of spin state and liquid-crystal transitions in Fe(II) metallomesogens was shown to be achievable by selecting a suitable parent SCO system and attaching it to the liquid-crystal moiety, with the aim of reaching the LS state or SCO properties at the temperature where the solid–liquid-crystal transition is expected (275–400 K) [391].

Although the number of examples of Fe(II) metallomesogens reported so far is small, the interplay/synergy between ST and liquid-crystal phase transitions has been seen to be very rich and the behaviors may be summarized as follows [358]:

1. systems with coupled phase transitions, subdivided into three groups: (a) where the structural changes associated with the Cr↔LC phase change drive the spin transition; (b) where the structural changes influence the spin state of the metallic centers but are not the driving force for the spin state transition; (c) where the vitrification of the material inhibits the SCO properties [375,392–394];
2. systems where both transitions coexist in the same temperature region but are not coupled [375,394–395];
3. systems with uncoupled phase transitions [358,394].

Presented below are some of the most representative examples of the Fe(II) metallomesogens where the Cr↔LC phase change drives the spin state transition (type 1) [394] and those metallomesogens for which the preparation of thin films was possible [375].

**Figure 34:**
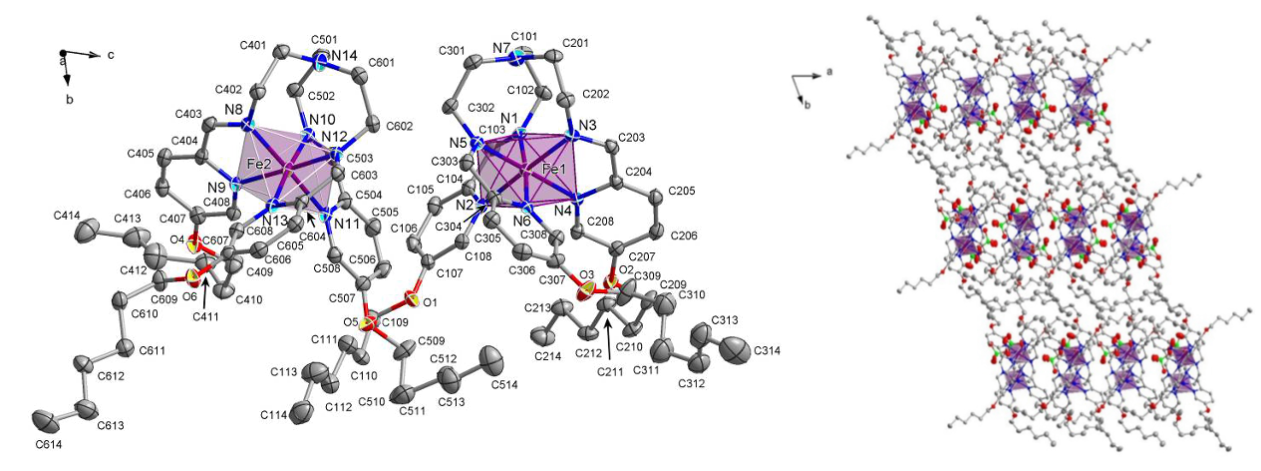
(left) Projection of the two independent cations of [Fe(C_6_–trenH)]^2^*^+^* with atom numbering scheme (150 K). (right) Projection of the molecular packing of the same complex along the *c* axis. Displacement ellipsoids are shown at 50% probability level. Hydrogen atoms and perchlorate anions are omitted for clarity. (Reproduced with permission from [394]. Copyright 2008 American Chemical Society).

The ligand tris[3-aza-4-((5-C*_n_*)(6-H)(2-pyridyl))but-3-enyl]amine (C*_n_*-trenH) has been reacted with FeCl_2_·sH_2_O salts to afforded a family of complexes with the general formula [Fe(C*_n_*-trenH)]Cl_2_·0.5H_2_O where *n* = 16, 18 and 20 [394]. A similar structure to that found for the derivative [Fe(C_6_-trenH)](ClO_4_)_2_ was obtained from analysis of the XPRD patterns at 293 K. Figure 34 (left) illustrates the molecular structure of this system. A pseudo-octahedral symmetry is adopted by the iron atoms, and they are surrounded by six nitrogen atoms belonging to imino groups and pyridines of the trifurcated ligand C*_n_*-trenH. Self-assembly to a bilayered composite results due to the amphiphilic nature of the alkylated molecules, with one layer being made up of polar head groups together with perchlorate anions (Figure 34 right). The nonpolar chains from oppositely oriented molecules meet together forming a hydrocarbon layer. The almost fully stretched alkyl chains are only distorted by the *gauche* conformation of some of the methylene groups and are tilted towards the *ac* plane, but they do not intertwine with chains of adjacent layers.

**Figure 35:**
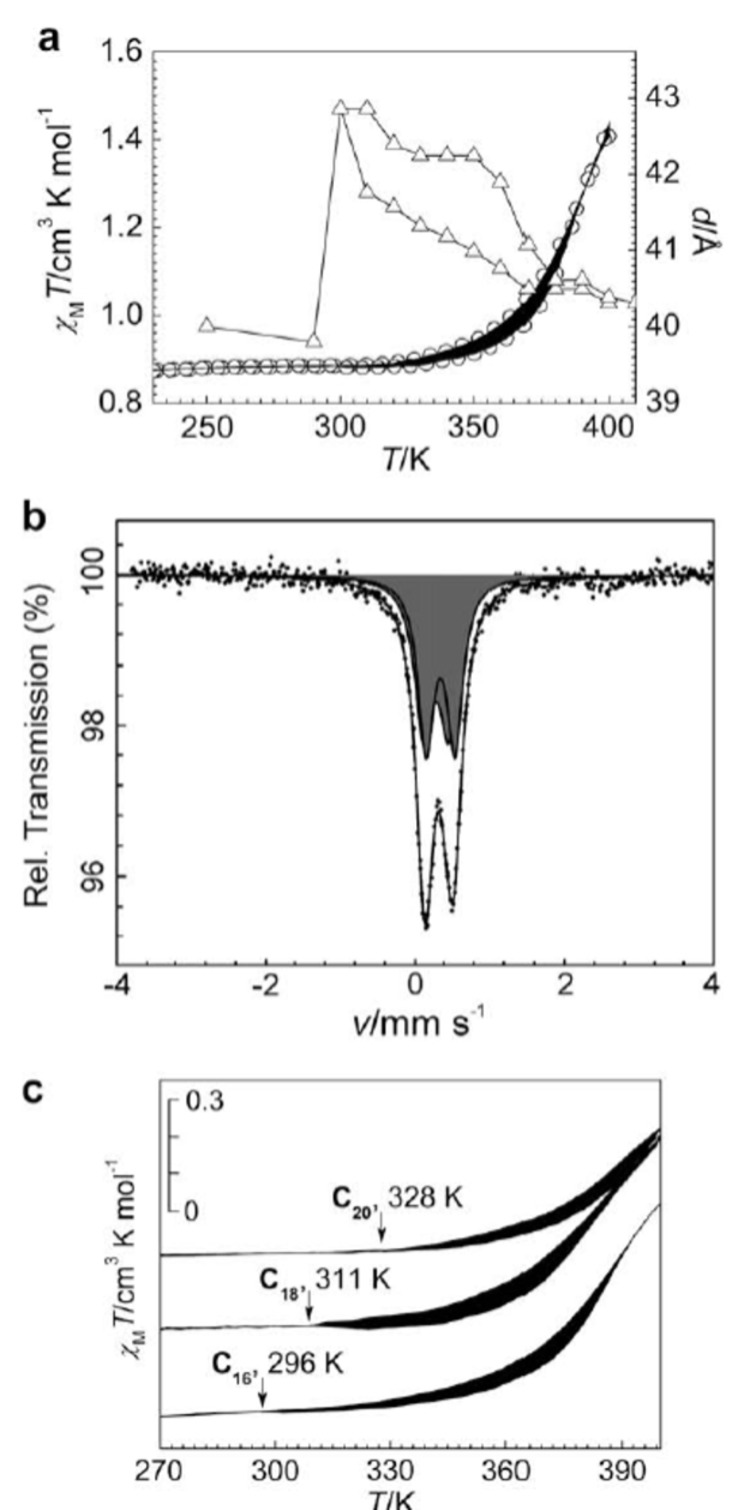
(a) *χ*_M_*T* versus *T* for [Fe(C_16_-trenH)]Cl_2_·0.5H_2_O and variation of the distance *d* with temperature (*T*) deduced from XPRD profiles after following the sequence 310–410–230–410 K. (b) Mössbauer spectra of [Fe(C_16_-trenH)]Cl_2_·0.5H_2_O measured at 80 K (HS doublet: light grey; LS doublet: dark grey). (c) *χ*_M_*T* versus *T* for [Fe(C*_n_*-trenH)]Cl_2_·0.5H_2_O (*n* = 16, 18 and 20), arrows indicate the temperatures at which the heating and cooling curves diverge. The black areas indicate the hysteresis loops. (Reprinted with permission from [394]. Copyright 2008 American Chemical Society).

As a consequence of the melting between 340 and 350 K for [Fe(C_16_-trenH)]Cl_2_·0.5H_2_O the temperature variation of the interlayer distances *d* presents an abrupt increase (Figure 35a). For derivatives with *n* = 16, 18 and 20 the melting was found at 296, 311 and 328 K, respectively. DSC and optical polarizing microscopy (OPM) further confirmed the temperature of melting. A smectic mesophase S_A_ has been identified based on these findings, with layered structures similar to [Fe(C_6_-trenH)](ClO_4_)_2_ [394].

Figure 35c displays the *χ*_M_*T* versus *T* plots for [Fe(C*_n_*-trenH)]Cl_2_·0.5H_2_O (*n* = 16, 18 and 20). Also shown is the Mössbauer spectrum measured for the derivative with *n* = 16 at 80 K (Figure 35b). The compounds are in the LS state in the temperature range of 4.2–290 K in which the Mössbauer spectra highlight the presence of a small residual HS fraction. For each of these derivatives a variation accompanied by a narrow hysteresis is seen in the *χ*_M_*T* versus *T* between 400 and 296 K (Figure 35c). Below 296 K, *χ*_M_*T* stays almost constant up to 10 K at which point it sharply decreases due to the zero-field splitting of the Fe(II) ions that remain in the HS state [394]. The thermally induced ST in [Fe(C*_n_*-trenH)]Cl_2_·0.5H_2_O begins immediately after the onset of the first-order phase transition (Cr↔S_A_). One can conclude that, since the ST is blocked below the temperature at which the compounds solidify, the spin transition is driven by the melting process in these metallomesogens. The hysteresis accompanying the ST in the S_A_ mesophase is due to the reorganization of the metallomesogen structure upon Cr↔S_A_ transition. It is worthy of mention that the ST temperature is dependent on the melting temperature, which is higher for the derivatives with longer alkyl chains (Figure 35c). In the LS state these compounds are dark purple, becoming light purple-brown in the HS state.

**Figure 36:**
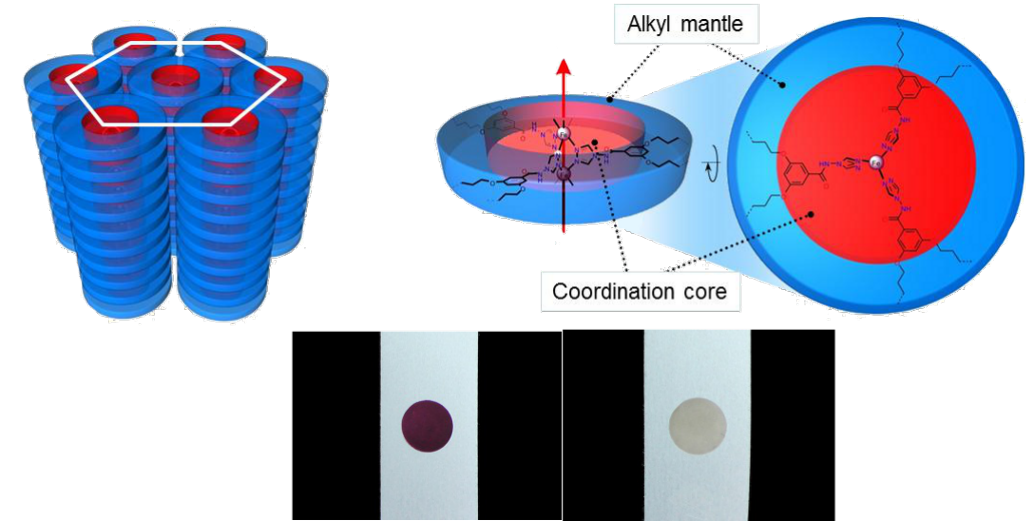
Schematic illustration of the structure of compounds [Fe(C_n_-tba)_3_]X_2_ adopting a columnar mesophase (top). Change of color in the SCO LC films around 60 °C (bottom). (Reprinted with permission from [375]. Copyright 2006 American Chemical Society).

An interesting and important feature is the possibility to obtain these materials in the form of thin films by exploiting their fluid nature. Thin films of a few micrometers thick have been formed by solvent evaporation for metallomesogens that exhibit Colh mesophase at room temperature. Examples are [Fe(C*_n_*-tba)_3_]A_2_ [C*_n_*-tba = 3,5-bis(alkoxy)-N-(4*H*-1,2,4-triazol-4-yl)benzamide, A = CF_3_SO_3_^−^, 4-MeC_6_H_4_SO_3_^−^ and *n* = 8, 10, 12] [375,392]. Reversible color change of the films by heating or cooling around 300 K, between violet (LS state) and white (HS state), was observed without fatigue (Figure 36).

#### SCO in gels

**Figure 37:**
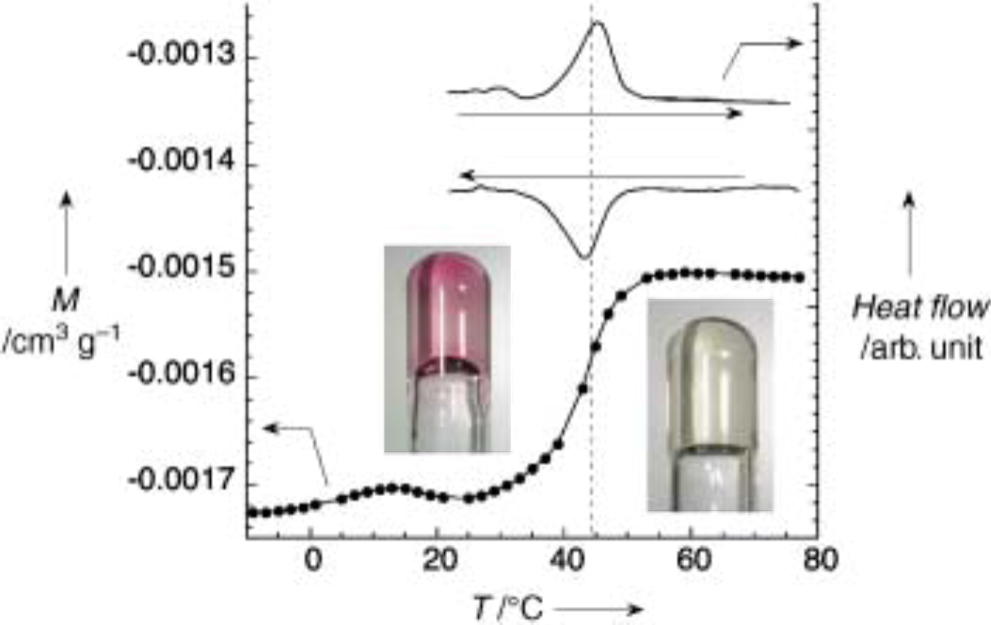
Temperature dependence of the magnetic moment (M) at 1000 Oe and DSC profiles (inset; 5 °C/min) of a 5 wt % of [Fe(II)(4-octadecyl-1,2,4-triazole)_3_](tosylate)_2_·2H_2_O in decane gel. The dotted line corresponds to the *T*_1/2_ = 44 °C deduced from DSC and magnetic measurements. The anomalies observed on the magnetic moment curve below 25 °C correspond to the solid–liquid first-order transition in pure decane [279]. (Reproduced with permission from [396]. Copyright 2004 Wiley-VCH Verlag GmbH & Co. KGaA).

The transfer of SCO properties into a soft-matter phase, such as a gel, has been accomplished by functionalizing the polymeric Fe(II)/4-R-1,2,4-triazole system with long alkyl chains on the 4-substituent [396–399]. By dissolution of these SCO materials in organic solvents, hybrid gels have been obtained, and their thermo-reversible magnetic, optical and rheological properties could be studied. It was shown that *T*_melt_ and *T*_1/2_ can be tuned by using mixtures of solvents and by changing the counter-anions, respectively (Figure 37) [396].

Compared to references dealing with nongelling aromatic solvents, a quick thermal response is characteristic of SCO physical gels, due to a rapid restoration of the H-bonding network, possibly a result of dynamic structural ordering through an enhanced lipophilic interaction of the self-assembling components in hydrocarbon solvents [397].

#### Spin crossover in porous coordination polymers

Porous coordination polymers (PCPs), also known as MOFs, represent a new class of functional molecular materials [400–407], which are able to mimic and even improve the functions of zeolites [408], i.e., storage [409–410], separation [411–412], sensing [413] and catalysis [414–416]. The vast majority of their applications are related to pore sizes, shapes, flexibility and structures/environments. Particularly interesting are those PCPs where the solid-state properties of the framework (magnetism [417], luminescence [418], conductivity [419–420], charge transport [421], etc.) change upon sorption/desorption of guest molecules. This new generation of PCPs acts in a sensory way expressing the host–guest interactions.

Among PCPs incorporating both porous and magnetic properties, it is noteworthy that these porous polymers are made up of bi-stable Fe(II) building units. The guest inclusion in the SCO framework can provoke the stabilization of the Fe(II) ions in the HS or LS state or has no effect at all. The resulting effect relies on the chemical nature and size of the guest molecules that occupy the pores. Detection of the sorption/desorption of guest molecules in the framework is performed by analyzing magnetic and optical outputs (the temperature dependence of the magnetic susceptibility and/or by the change of color at a given temperature). In principal, a fingerprint in the form of a magnetic response pattern may be attainable for distinct analytes.

Examples of Fe(II) SCO-PCPs reported up to date are still scarce. The first report on a microporous coordination polymer was based on the ligand dpe (1,2-di(4-pyridyl)ethylene)(*trans* isomer) [331] and dates back to 1995. Almost a decade later, a detailed investigation was published of the guest influence on the SCO properties in analogous frameworks with composition [Fe(L)(NCS)_2_]·G (L = azpy and bped (1,2-bis(4-pyridyl)-1,2-ethanediol)) [227]. In these SCO-PCPs the adsorption of guest molecules induces some stabilization of the framework in the LS state. Typically they show gradual SCO in the temperature region 10–200 K.

**Figure 38:**
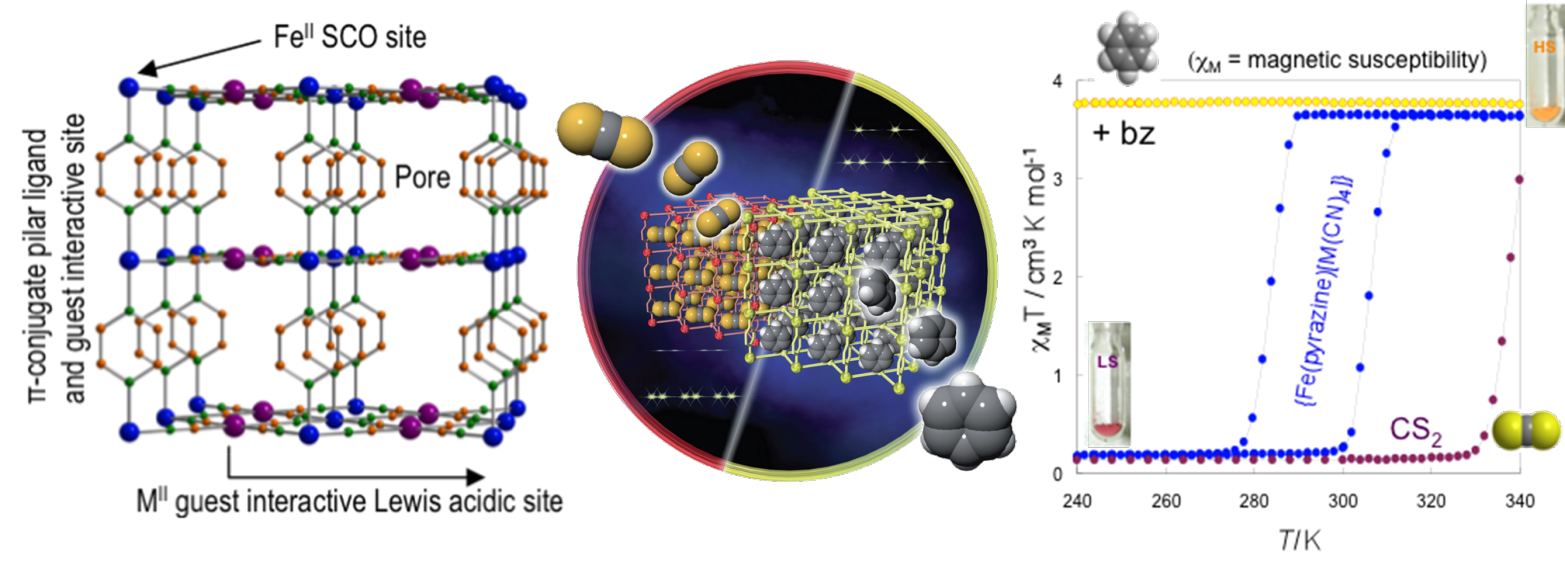
Porous structure of the SCO-PMOFs {Fe(pz)[M(II)(CN)_4_]} (left), representation of the host–guest interaction (middle) and change of the spin state upon guest adsorption (right) [paramagnetic, yellow (C_6_H_6_); diamagnetic, red CS_2_]. (Reproduced with permission from [422]. Copyright 2009 Wiley-VCH Verlag GmbH & Co. KGaA).

More recently a drastic influence of the guest inclusion on *T*_1/2_ and cooperativity has been reported for the 3D Hofmann-like SCO-PCPs {Fe(L)[M(CN)_4_]}·G [L = pz [363,422–425]; azpy [382]; bpac (bis(4-pyridyl)acetylene) [426]; dpe (*trans* isomer) [300] M(II) = Ni, Pd, Pt)]. This type of framework provides two guest interactive sites, one between the organic bridges (site A) and another between the four-coordinate M centers (site B) (Figure 38).

Particularly interesting are the results obtained with the SCO-PCP {Fe(pz)[M(CN)_4_]}. In fact, this represents a new generation of functional materials responding to their environment since the framework can allow reversible control of the magnetic and optical outputs through the chemical response at room temperature [363,422–425]. The reversible spin state transition at the Fe(II) sites occurs concomitantly with the uptake of guest molecules. Hydroxy solvents, five- and six-membered aromatic molecules, stabilize the HS state (yellow color) while CS_2_ (for M = Pt) [422] or CH_3_CN (for M = Ni) [423] favor the LS state (red color). Desorption of guest molecules under vacuum produces the guest-free framework in the induced spin state. The initial HS or LS state is not recovered by the system after release of the guest molecules within the bistable temperature region. Information about adsorption of the guest molecules in the form of the spin state, magnetism, color, and structure are retained by this memory function; information that can be erased by using the appropriate operator (i.e., by applying either ∆T − ∆T or −∆T + ∆T, from room temperature) (Figure 38).

**Figure 39:**
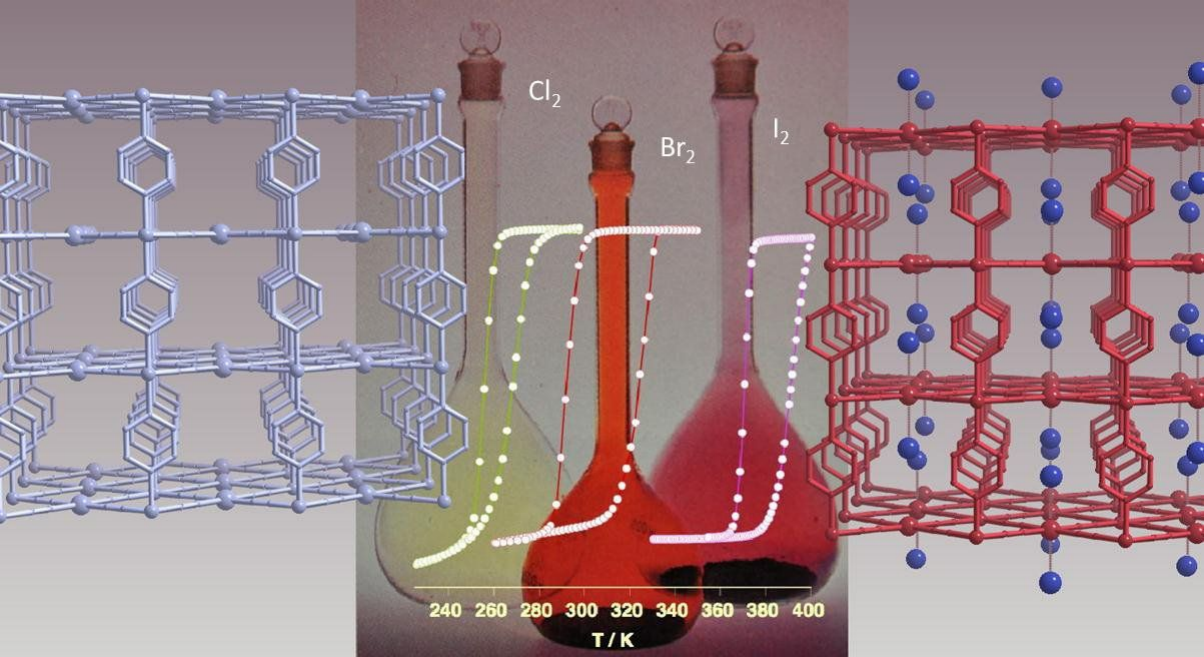
Porous structure of the guest-free SCO-PMOF’s {Fe(pz)[M(II)(CN)_4_]} (left), magnetic properties of the {Fe(pz)[Pt(CN)_4_(X)]} (X = Cl^−^, Br^−^ and I^−^) compounds (middle) and crystal structure of compound {Fe(pz)[Pt(CN)_4_(I)]}. (Reproduced with permission from [424]. Copyright 2009 Wiley-VCH Verlag GmbH & Co. KGaA).

Moreover, the occurrence of coordinative unsaturated metal centers M(II) provided enhanced adsorptive selectivity for dihalogen molecules. Indeed, an associative oxidation of Pt(II) to Pt(IV) and reduction of the dihalogen to the corresponding halide to give {Fe(pz)[Pt(CN)_4_(X)]} (X = Cl^−^, Br^−^ and I^−^) has been demonstrated (Figure 39) [424]. Particularly, successful control of the *T*_1/2_ value from 300 to 400 K while maintaining bistability (25 K wide thermal hysteresis) has been achieved for {Fe(pz)[Pt(CN)_4_(I)_n_]} (*n* = 0–1) [425].

**Figure 40:**
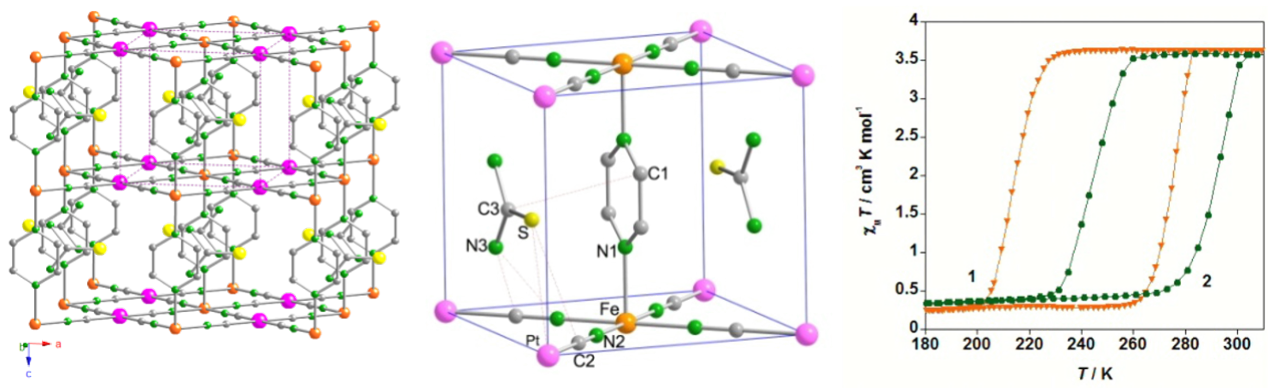
(left) The 3D porous structure of {Fe(pz)[Pt(CN)_4_]}·0.5(CS(NH_2_)_2_) (**1**) and {Fe(pz)[Pd(CN)_4_]}·1.5H_2_O·0.5(CS(NH_2_)_2_) (**2**) in a direction close to [101]. Atom code: Fe (orange), Pt (pink), N (green), and C (grey). (right) Magnetic properties of **1** and **2** in the form of *χ*_M_*T* versus *T*. (Reproduced with permission from [428]. Copyright 2012 Royal Society of Chemistry).

Inclusion of thiourea molecules capable of interacting with the organic pillar ligands and the unsaturated metal coordination sites through weak intermolecular interactions leads to the clarthrate compounds formulated as {Fe(pz)[Pt(CN)_4_]}·0.5(CS(NH_2_)_2_) and {Fe(pz)[Pd(CN)_4_]}·1.5H_2_O·0.5(CS(NH_2_)_2_) [428]. These 3D porous coordination polymers exhibit unprecedented ST accompanied by large thermal hysteresis cycles of ca. 60 K wide. Apparently, this strong cooperative ST arises from the fact that multiple significant intermolecular interactions have to be reorganized upon the SCO process, since the dimensions of the pores are different in the LS and HS states (Figure 40).

Current development of this topic points at increasing the pore size and inner pore functionality of the SCO-PCPs. The goals are twofold: (i) develop novel SCO-PCPs for sensing the uptake/release of aromatic guest molecules in solution or air; and (ii) to study selective adsorption and separation of aromatic molecules on SCO-PCPs. In comparison with gas storage investigations on PCPs, systematic studies of guest adsorption and separation on PCPs for which single-crystal information is available are scarce, particularly for aromatic molecules [425]. However, the separation and capture of aromatic molecules is very important in industry and for the development of green environments [415–416].

**Figure 41:**
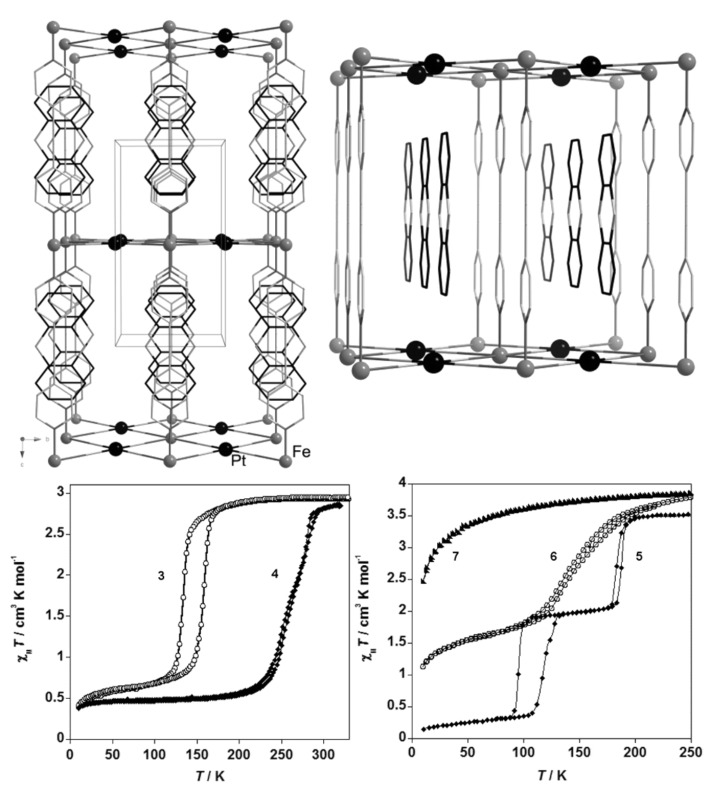
Top: The 3D porous structure of {Fe(dpe)[Pt(CN)_4_]}·phenazine in a direction close to [101] emphasizing the guest inclusion in the framework. Bottom: Magnetic properties of {Fe(dpe)[Pt(CN)_4_]}·0.5(dpe) (**3**), {[Fe(dpe)[Pt(CN)_4_]}·0.5(dpe)·H_2_O (**4**), and {Fe(dpe)[Pt(CN)_4_]}·nG (n = 1; G = phenazine (**5**), anthracene (**6**) and naphthalene (**7**)) in the form of χ_M_*T* versus *T*. (Reproduced with permission from [300]. Copyright 2008 Wiley-VCH Verlag GmbH & Co. KGaA).

In this respect, a novel Hoffman-like 3D CP {Fe(dpe)[Pt(CN)_4_]}·0.5(dpe) (**3**) based on the organic pillar ligand dpe (1,2-di(4-pyridyl)ethylene) (trans isomer) has been recently reported. This compound is commonly crystallized in its monohydrate form {[Fe(dpe)[Pt(CN)_4_]}·0.5(dpe)·H_2_O (**4**). The water molecule can be reversibly sorbed/desorbed while maintaining crystallinity. The guest-free framework undergoes strong cooperative ST located at *T*_1/2_^↓^ = 135 K and *T*_1/2_^↑^ = 150 K while the water-containing framework exhibits a two-step ST in the interval of 275–240 K. Interestingly, clathrates formulated as {Fe(dpe)[Pt(CN)_4_]}·*n*G (*n* = 1; G = phenazine (**5**), anthracene (**6**) and naphthalene (**7**)) are obtained when the guest molecules are present during the crystallization process (Figure 41) [427]. Detection of the phenazine, anthracene or naphthalene encapsulation in the framework can be done by recording the magnetic susceptibility at a given temperature since the magnetic properties of the clathrate compounds are very different. The fact that the clathrates present abrupt, incomplete or two-step ST or paramagnetic behavior is related to their size, shape, location and interaction with the framework. In general, it is observed that clathration of phenazine, anthracene or naphthalene leads to the stabilization of the frameworks in the HS state. In the case of **6** and **7** the guest molecules would be most probably located between the dpe pillar ligands in analogy with **5**. However, the particular stabilization of the HS state seems to be regarded with a possible tilt of the guest molecules with respect to the pillar ligands dpe, as observed for the clathrate {Fe(pz)[M(CN)_4_]}·pz [422]. This particular location of guest molecules prevents the intrinsic contraction–expansion of the framework associated with the spin transition.

#### Combination of SCO and electrical conductivity

In molecular science, the fundamental property of electrical conductivity is currently being investigated. Spurred on by finding of electrical conductivity in perylene bromide, researchers have been working hard to produce high *T*_c_ superconductors based on organic and organometallic charge-transfer complexes and radical-ion salts [429]. Currently, the field of molecular conductors tends towards the design of hybrid materials in which a second interesting physical property is incorporated.

Two different synthetic approaches can be outlined with regard to the combination of SCO and electrical conductivity properties. First, a recently proposed strategy is the assembly of switchable molecular SCO building blocks with radical ionic salts, to tune (switch on/off) the conducting properties by acting on the bistable cation. A separate strategy involves the covalent linkage of paramagnetic metal ions to redox-active ligands. By associating redox-active moieties and heteroatom-based ligands capable of coordinating to the metallic center, a novel approach toward the modulation of the collective electronic properties of the resulting bifunctional material is realized.

**Figure 42:**
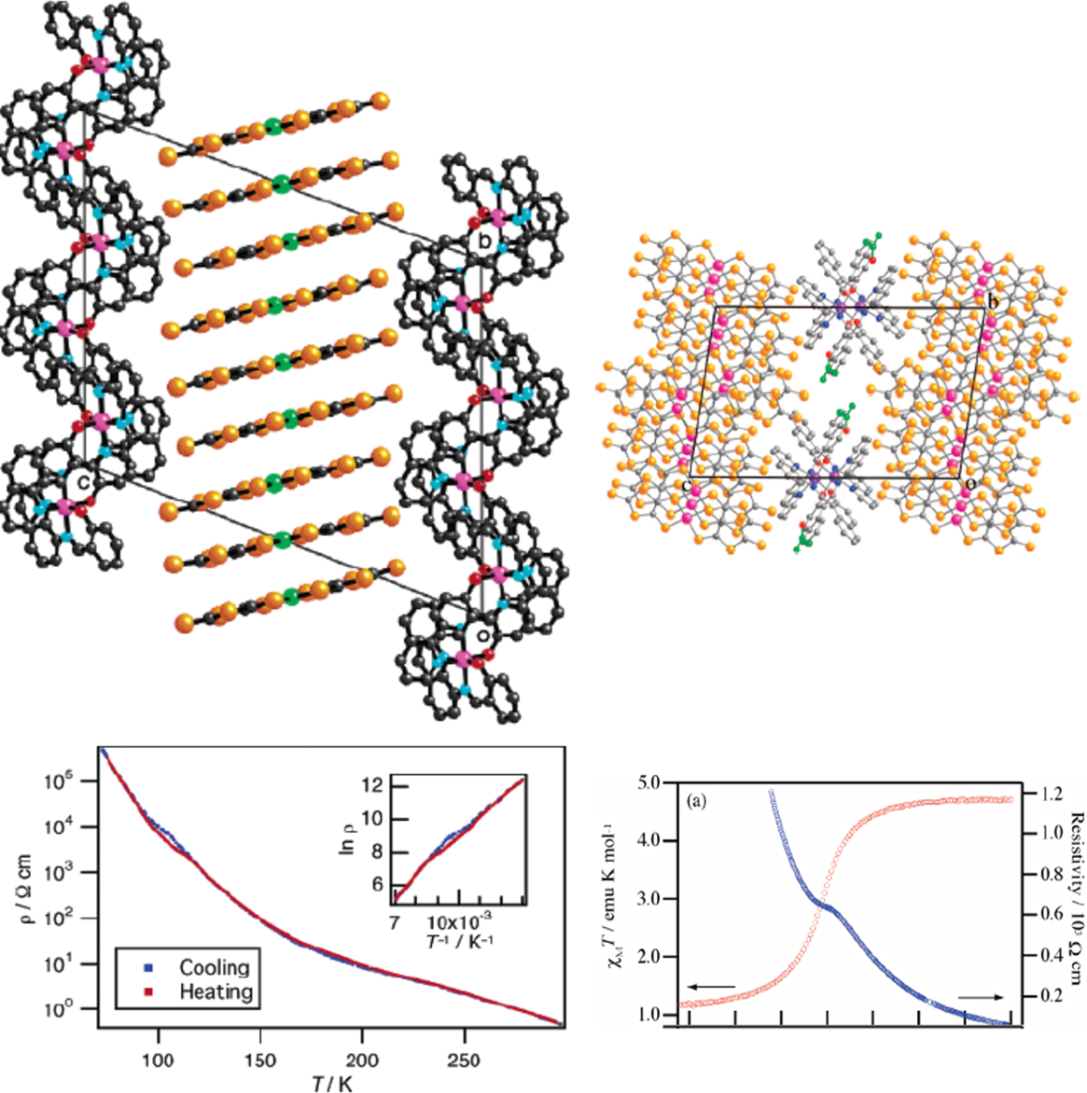
View of the segregated stacking of [Ni(dmit)_2_]^−^ and [Fe(sal_2_-trien)]^+^ in [Fe(qsal)_2_][Ni(dmit)_2_]_3_·CH_3_CN·H_2_O (left) and in [Fe(qnal)_2_][Pd(dmit)_2_]_5_·(CH_3_)_2_CO (right) together with magnetic and resistivity plots. [430]. (Reproduced with permission from [431] and [432]. Copyright 2006 & 2008 American Chemical Society).

The former strategy has led to the compounds {[Fe(sal_2_-trien)][Ni(dmit)_2_]_3_} [430], [Fe(qsal)_2_][Ni(dmit)_2_]_3_·CH_3_CN·H_2_O [431] and [Fe(qnal)_2_][Pd(dmit)_2_]_5_·(CH_3_)_2_CO [432]. These materials exhibit a structural arrangement typical of [M(dmit)_2_]^x^ (M = Ni, Pd) fractional-oxidation-state compounds, where the [Ni(dmit)_2_]^−^ or [Pd(dmit)_2_]^x^ units interact through S···S short contacts, defining layers, which alternate with layers of [Fe(L)]^+^ (Figure 42). The cationic units undergo cooperative SCO behavior and the layers of [M(dmit)_2_]^x^ enables the occurrence of electronic transport. At room temperature the electrical conductivity of these hybrid complexes is in the range of 0.1–2.0 S cm^−1^. Interplay between SCO and electrical conductivity has been observed for [Fe(qnal)_2_][Pd(dmit)_2_]_5_·(CH_3_)_2_CO [432]. The cooperative SCO phenomenon is considered to induce a chemical uniaxial strain effect on the conducting layer. Such a cooperativity based on supramolecular interactions is crucial for controlling or switching the electronic properties of a molecular solid by external stimuli.

**Figure 43:**
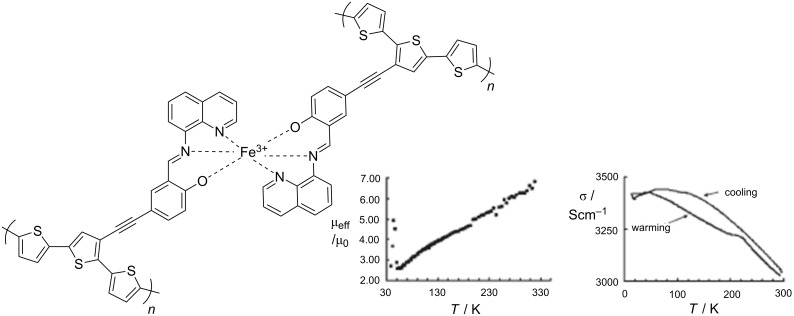
Thin films based on Fe(III) compounds coordinated to Terthienyl-substituted QsalH ligands [434] together with magnetic and resistivity plots. (Reproduced with permission from [434]. Copyright 2009 American Chemical Society).

The second approach has as yet been less developed due to the difficulties implicit in the organic synthesis of ligands containing redox-active moieties and heteroatom-based ligands. Figure 43 illustrates two examples of Fe(II) coordination compounds derived from this approach [433–434]. In both cases, the magnetic and electrical resistivity measurements suggested an interaction of ST and electrical conductivity, since the electrical conductivity shows an anomaly around the critical ST temperature.

#### Combination of SCO and fluorescence properties

Combining SCO and fluorescence in a single material dates back to the first report on a Ni(II) tetraaza-macrocyclic complex [435] followed by iron(II) SCO complexes studied as thin films [436]. In general, fluorescence quenching was observed in one spin state, as found for the heterodinuclear triple helicate iron(II) complex including a luminescent ion (Eu) [437] or for the 1D-1,2,4-triazole iron(II) chain including pyrene [438]. When this later fluorophore was decorated on a ligand of an Fe(II) mononuclear complex [439], temperature-dependent studies of the photophysical properties did not reveal any obvious correlation between the fluorescence of the pyrene group and the spin state of the Fe(II) ion.

The celebrated 1D polymeric iron(II) chain system provided hybrid materials showing fluorescent properties tuned by the spin state transition. Hybrid nanoparticles containing SiO_2_ and [Fe(Htrz)_2_(trz)]BF_4_ were coated with the fluorophore 3-(dansylamido)propyltrimethoxysilane (dansyl) [286]. The hybrid nanoparticles preserve the magnetic bistability and, as a new property resulting from the hybrid material, the luminescence is coupled with the spin state transition showing bistability as well. Similar synergy between spin state transition and luminescence has been observed in solution in nanoparticles of [Fe(NH_2_trz)_3_](tos)_2_ coated with rhodamine-110 chloride (9-(2-carboxyphenyl)-3,6-diamino-3*H*-xanthylium chloride). This multiproperty material has been proposed as a prototype for fluorescent thermometry [440].

**Figure 44:**
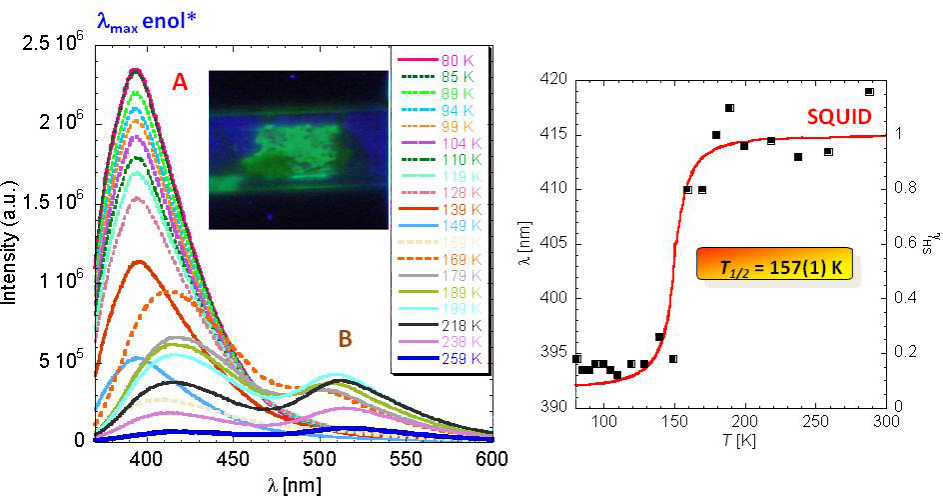
Left: Temperature-dependent emission spectra for [Fe_2_(Hsaltrz)_5_(NCS)_4_]·4MeOH at λ^ex^ = 350 nm over the range 80–259 K. The inset shows the fluorescence at 293 K produced by continuous irradiation at 254 nm. Right: Temperature dependence of λ_max_ enol* in the emission spectrum (■), which follows *γ_HS_* derived from SQUID data (solid line) [235].

The first observation of the tracking of a ST by fluorescence in the crystalline state has recently been made for the dinuclear Fe(II) complex [Fe_2_(Hsaltrz)_5_(NCS)_4_]·4MeOH [235]. In this complex, a fluorophore tag whose fluorescence is due to different prototropic forms of a *N*-salicylidene aniline (enol* and *cis*-keto* forms) [441] was directly grafted on to the 1,2,4-triazole ligand (Figure 21). The wavelength emission maximum of the enol* band (λ_max_ enol*) could be used as a marker to track the SCO event by fluorescence spectroscopy. As shown in Figure 44, a drastic jump of 20 nm was observed over the temperature range of 135–179 K, from ~395 nm below 150 K to 415 nm above 200 K. Remarkably, such a dramatic increase in the wavelength precisely follows the temperature dependence of *γ*_HS_ derived by SQUID measurements, and the transition temperature, *T*_1/2_ = 157(1) K, is in good agreement with the one obtained by DSC [*T^max^* = 155(1) K] [235].

## Conclusion

In this article we have described a special class of molecular switches based on a dynamic electronic-structure phenomenon known as *spin crossover*. The principle of the molecular switching is a change of the spin state of a transition metal ion that is located in the center of complex molecules and coordinated to ligand atoms or molecules. Depending on the strength of the ligand field, i.e., the electrostatic field exerted at the central metal ion, the valence electron configuration of the metal can switch between two stable electron arrangements, one with maximum spin multiplicity, known as the high spin (HS) state, and one with minimum spin multiplicity, the low spin (LS) state. This switching can be stimulated by variation of temperature, application of pressure, light irradiation, and other external stimuli. The two phases involved have drastically different magnetic and optical properties, which provide the means for detection of the spin state phases. The spin crossover phenomenon occurs in numerous classes of coordination compounds; by far the majority of them are coordination compounds containing iron(II), iron(III) or cobalt(II). We have presented a selection of spin crossover compounds of iron(II) classified as mono-, di- and oligonuclear systems as well as 1D, 2D and 3D coordination polymers. As the latest developments in this area, preparation and studies of materials on the nanoscale have been illustrated. Likewise, systems combining spin crossover and other physical or chemical properties, such as liquid-crystalline and gel behavior, electric conductivity, fluorescence and porous properties, have been covered. In nearly all these classes of spin crossover compounds it is important and necessary to go beyond the borderline of classical coordination chemistry and make use of strategies for the synthesis of new organic ligands and of organic synthesis techniques that would provide novel systems and functionalities.

Spin crossover compounds bear the potential for practical applications. Molecular electronics, data storage, display devices and sensors are objectives currently under consideration.
